# WNK1 mediates M-CSF-induced macropinocytosis to enforce macrophage lineage fidelity

**DOI:** 10.1038/s41467-025-59901-0

**Published:** 2025-05-28

**Authors:** Alissa J. Trzeciak, Zong-Lin Liu, Mohamed Gatie, Adam S. Krebs, Waleska Saitz Rojas, Anya J. O’Neal, Ann K. Baako, Zhaoquan Wang, Justin Nelson, Isabella C. Miranda, Jazib Uddin, Allie Lipshutz, Jian Xie, Chou-Long Huang, Pedro H. V. Saavedra, Anna-Katerina Hadjantonakis, Michael Overholtzer, Michael S. Glickman, Arohan R. Subramanya, Thomas Vierbuchen, Jon Iker Etchegaray, Christopher D. Lucas, Christopher N. Parkhurst, Justin S. A. Perry

**Affiliations:** 1 Immunology Program, Memorial Sloan Kettering Cancer Center, New York, NY USA; 2 Louis V. Gerstner Jr. Graduate School of Biomedical Sciences, Memorial Sloan Kettering Cancer Center, New York, NY USA; 3 Developmental Biology Program, Sloan Kettering Institute for Cancer Research, New York, NY USA; 4 Department of Immunology and Microbial Pathogenesis, Weill Cornell Medicine, New York, NY USA; 5 Division of Pulmonary and Critical Care Medicine, Weill Cornell Medicine, New York, NY USA; 6 Jill Roberts Institute for Research in Inflammatory Bowel Disease, Weill Cornell Medicine, New York, NY USA; 7 Department of Medicine, Division of Nephrology, Carver College of Medicine, University of Iowa, Iowa City, IA USA; 8 Department of Biology, Northeastern University, Boston, MA USA; 9 Cell Biology Program, Sloan Kettering Institute for Cancer Research, New York, NY USA; 10 Division of Infectious Diseases, Memorial Sloan Kettering Cancer Center, New York, NY USA; 11 Dept of Medicine, Renal-Electrolyte Division, University of Pittsburgh School of Medicine, Pittsburgh, PA USA; 12 Aging and Metabolism Research Program, Oklahoma Medical Research Foundation, Oklahoma City, OK USA; 13 University of Edinburgh Centre for Inflammation Research, Queen’s Medical Research Institute, Edinburgh, BioQuarter UK; 14 Institute for Regeneration and Repair, Edinburgh, BioQuarter UK

**Keywords:** Myelopoiesis, Gene regulation in immune cells, Endocytosis, Monocytes and macrophages

## Abstract

Tissue-resident macrophages (TRM) are critical for mammalian organismal development and homeostasis. Here we report that with-no-lysine 1 (WNK1) controls myeloid progenitor fate, with *Csf1r*^iCre^-mediated *Wnk1* deletion in mice (WNK1-deficient mice) resulting in loss of TRMs and causing perinatal mortality. Mechanistically, absence of WNK1 or inhibition of WNK kinase activity disrupts macrophage colony-stimulating factor (M-CSF)-stimulated macropinocytosis, thereby blocking mouse and human progenitor and monocyte differentiation into macrophages and skewing progenitor differentiation into neutrophils. Treatment with PMA rescues macropinocytosis but not macrophage differentiation of WNK-inhibited progenitors, implicating that M-CSF-stimulated, macropinocytosis-induced activation of WNK1 is required for macrophage differentiation. Finally, M-CSF-stimulated macropinocytosis triggers WNK1 nuclear translocation and concomitant increased protein expression of interferon regulatory factor (IRF)8, whereas inhibition of macropinocytosis or WNK kinase activity suppresses IRF8 expression. Our results thus suggest that WNK1 and downstream IRF8-regulated genes are important for M-CSF/macropinocytosis-mediated regulation of myeloid cell lineage commitment during TRM development and homeostasis.

## Abstract (web summary)

Tissue-resident macrophages (TRM) are important mediators of local immunity. Here the authors show that the deficiency or inhibition of a kinase, WNK1, unlinks macrophage colony-stimulating factor signaling and resulted macropinocytosis with the downstream, potentially IRF8-mediated genetic program to bias progenitor differentiation to neutrophil instead of TRM.

## Introduction

Macrophages are innate immune cells that play an indispensable role as the body’s professional phagocyte that facilitate multicellular organismal development and maintain tissue homeostasis throughout life^1–3^. As long-lived sentinels present in all tissues and organs, tissue-resident macrophages (TRMs) are often the first line of defense, both combating invading pathogens and recruiting effector cells to help resolve infection.^4–7^ Moreover, TRMs are responsible for the homeostatic clearance of apoptotic cells, termed efferocytosis, a process that is essential during embryonic development and throughout life^8–11^. Recently, we reported that internalization of apoptotic cells induces unique transcriptional programs within macrophages, including anti-inflammatory and reparative programs, despite the drastic increase in levels of potentially inflammation-inducing solutes^12^. As part of this work, we defined a “rapid-response circuit” (reviewed in ref. ^11^) that regulates intracellular Cl^–^ flux and macrophage volume during efferocytosis^11,12^. This circuit involves with-no-lysine 1 (WNK1), a serine-threonine kinase that acts as a sensor of intracellular Cl^–^levels^13^. During a phagocytic or endocytic event, Cl^–^ flux to the cytosol increases and then is exported back to the extracellular space^14^. Cl^–^ efflux from the cytosol activates WNK1, which then phosphorylates the kinases SPAK and/or OSR1, which in turn phosphorylate the solute carrier transporters (SLC)12A2 and SLC12A4, allowing for rebalancing of cytosolic ion levels and inactivation of the WNK1/SPAK/OSR1 pathway. Thus, to prevent potentially catastrophic consequences, macrophages utilize solute-sensing and -transporting pathways to remain healthy, tolerogenic, and capable of subsequent apoptotic cell uptake.

Given our previous work demonstrating the importance of WNK1 for homeostatic efferocytosis in vitro, we sought to determine if TRM-specific genetic perturbation of WNK1 results in autoimmunity or inflammatory disease. Unexpectedly, mice with *Csf1r* promoter-driven Cre-mediated deletion of *Wnk1* (WNK1-deficient) exhibited failure to thrive and were unable to live beyond four weeks of age. Strikingly, WNK1-deficient mice broadly lacked TRMs across most organs assessed, displayed pathological tissue/organ development, and exhibited systemic neutrophil infiltration. Furthermore, neonatal myeloid progenitors, bone marrow progenitors, and Ly6C+ monocytes failed to develop into macrophages when lacking WNK1 or WNK activity is inhibited in vitro, and when progenitors or monocytes are transplanted into wild-type adult mice in vivo, with progenitors unexpectedly differentiating into neutrophils instead. Importantly, human induced pluripotent stem cells (iPSCs) and CD14+ monocyte cells also require WNK kinase activity for appropriate macrophage development, as small molecule inhibition of WNK kinase activity blocked macrophage differentiation, instead skewing differentiation into neutrophils. Interestingly, absence of TRMs and not neutrophilia appears to account for WNK1-deficient mouse death, as delivery of wild-type monocytes or bone marrow progenitors into perinatal day (P)2 mice fully rescues survival. Mechanistically, we show that both mouse and human progenitors and monocytes treated with M-CSF perform macropinocytosis, an actin-dependent, clathrin- and caveolin-independent endocytosis pathway used to sample substrates in the extracellular fluid^15^. Strikingly, WNK1-deficiency or inhibition of WNK kinase activity significantly decreased continuous M-CSF-stimulated macropinosome uptake by mouse CSF1R^high^ bone marrow progenitors, human iPSCs, and mouse and human monocytes. On the other hand, overexpression of WNK1 in progenitors significantly boosted M-CSF-stimulated macropinocytosis. Importantly, blocking M-CSF-stimulated macropinocytosis via selective inhibition abrogated differentiation of mouse and human progenitors and monocytes in vitro, instead shunting differentiation towards neutrophil development, whereas WNK1 overexpression in progenitors enhanced the rate of macrophage differentiation. Furthermore, treatment with the phosphoinositide 3-kinase (PI3K) activator phorbol-12-myristate-13-acetate (PMA) rescued macropinocytosis activity but not macrophage differentiation of WNK kinase activity-inhibited progenitors, suggesting that M-CSF-stimulated macropinocytosis, via direct WNK1 activity, is required for differentiation of macrophages. Finally, M-CSF-, but not PMA-, stimulated macropinocytosis triggers CSF1R internalization and concomitant WNK1 nuclear translocation, which in turn increases protein expression of the macrophage lineage-supporting transcription factor interferon regulatory factor (IRF)8. Importantly, inhibition of macropinocytosis or WNK kinase activity suppresses, whereas WNK1 overexpression enhances IRF8 expression. Collectively, we have elucidated a novel role of WNK1 for TRM development.

## Results

### *Csf1r*^iCre^-mediated deletion of WNK1 results in premature mortality

**Fig. 1:**
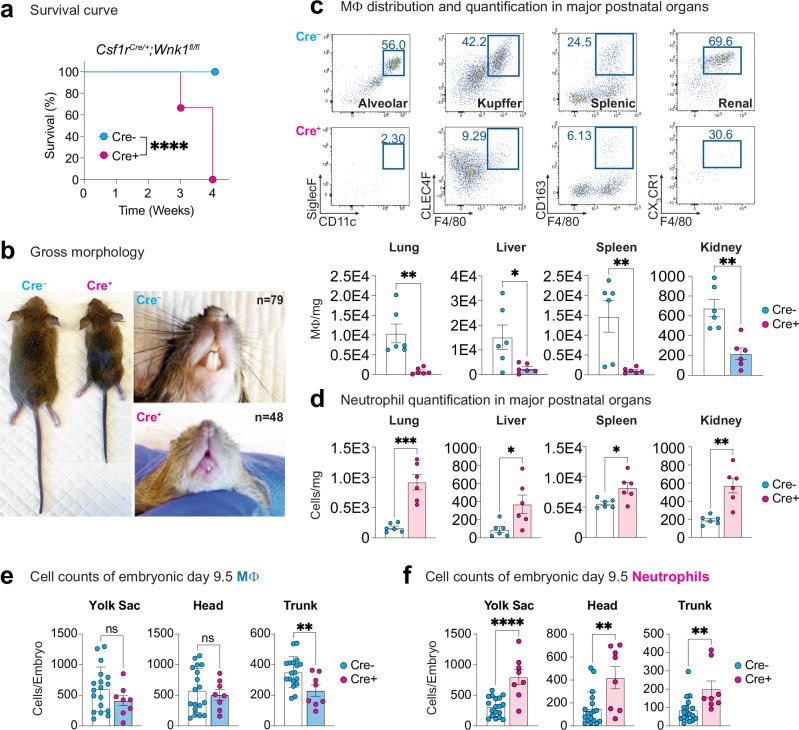
*Csf1r*^iCre^-mediated deletion of *Wnk1* results in rapid mortality, MΦ deficiency, and neutrophilia. **a** *Csf1r*^iCre^-mediated deletion of WNK1 results in early mortality. Survival curve of *Csf1r*^*Cre+*^*; Wnk1*^*fl/fl*^ (Cre + , magenta) vs. *Csf1*^*Cre–*^*; Wnk1*^*fl/fl*^ (Cre-, blue) mice. Litters were monitored from birth and sacrificed humanely when failure to thrive criteria were met. Data are from *n* = 74 Cre- mice and *n* = 48 Cre+ mice. Differences in survival were determined using the Mantel-Cox test. ****p < .0001. **b** Analysis of gross morphology reveals severe underdevelopment. Representative images of three- to four-week-old Cre- and Cre+ mice body (top) and teeth (bottom). Images are representative of *n* = 74 Cre− mice and *n* = 48 Cre+ mice. (Right) Representative images of organs from Cre− to Cre+ mice. Shown are (clockwise from top left) brain, spleen, colon, kidneys, bones, liver, heart, and lungs. Images are representative of organ analysis from six Cre− mice and six Cre+ mice. See Supplementary Fig. 1 for additional gross morphology and organ size analysis. **c** Flow cytometric analysis of macrophage subsets in major organs. Flow cytometry analysis of CD45+ tissue-resident macrophages in Cre+ (*n* = 6, magenta dots) and Cre− (*n* = 6, blue dots) 3-to-4-week-old mice. Subsets were first gated on live CD45+ CD11c− CD11b+ (except for lung macrophages; see Supplementary Fig. 2a for flow cytometry gating strategy). Tissue-resident macrophages were gated as follows: alveolar macrophage (CD11c^high^ CD11b^low^ SiglecF+), Kupffer cells (Ly6C− Ly6G− CLEC4F+ F4/80+), splenic red pulp macrophages (Ly6C− Ly6G− CD163+ F4/80+), and kidney macrophages (Ly6C− Ly6G− CX_3_CR1+ F4/80+). Images (top) and summary plots (bottom) of absolute numbers per milligram of tissue are from four independent experiments. Data are shown as mean ± SEM. Statistical significance was determined via two-tailed independent samples *t*-test. *p < .05, **p < .01. See also Supplementary Fig. 2b, c for analysis of additional tissues and Supplementary Fig. 3 for immunofluorescence (IF) analysis. **d** Flow cytometric analysis of neutrophilia in major organs. Flow cytometry analysis of neutrophils (Ly6G+) in major tissues from Cre+ (*n* = 6, magenta dots) and Cre− (*n* = 6, blue dots) 3-to-4-week-old mice (see Supplementary Fig. 2a for flow cytometry gating strategy). Shown are summary plots of absolute numbers per milligram of tissue from four independent experiments. Statistical significance was determined via two-tailed independent samples *t*-test. *p < .05, **p < .01, ***p < .001. See also Supplementary Fig. 2e–h for analysis of additional tissues, Supplementary Fig. 3 for IF analysis, and Supplementary Fig. 4 for morphological and functional analysis of Cre− and Cre+ neutrophils. **e**, **f** Flow cytometric analysis of embryonic macrophages and neutrophils in E9.5 embryos. Eight pregnant dams were euthanized at E9.5, and embryos were isolated, genotyped, and analyzed. Shown are summary plots of the absolute counts from flow cytometry analysis of CD11b^Low^ F4/80+ macrophages (**e**) and CD11b^High^ Ly6C+ Ly6G+ fetal neutrophils (**f**) in Cre− (*n* = 18, blue dots) and Cre+ (*n* = 8, magenta dots) E9.5 embryos. Yolk sac (left), head (middle), and trunk (right) were separated to delineate yolk sac-derived and brain-derived macrophages from tissue-resident macrophages in other organs. Pooled data are from eight independent experiments with between two and six biological replicates per experiment. Data are shown as mean ± SEM. Statistical significance was determined via two-tailed independent samples *t*-test. **p < .01, ****p < .0001, ns = not significant. See also Supplementary Figs. 2a and 6a, b for flow cytometry gating strategy and Supplementary Fig. 6c–h for analysis of E9.5 erythromyeloid progenitors and monocytes. Source data are provided in a Source Data file included with this manuscript.

To interrogate the importance of WNK1 for TRM function, we bred *Wnk1*^*fl/fl*^ mice^16^ to mice expressing Cre under the control of the colony-stimulating factor 1 receptor (*Csf1r*) promoter. The resultant conditional loss of *Wnk1* (*Csf1r*^*iCre+*^;*Wnk1*^*fl/fl*^; WNK1-deficient mice) led to significantly decreased fecundity and dramatic premature death of all Cre+ mice at 3–4 weeks of age (Fig. 1a and Supplementary Fig. 1a, b). Upon gross pathological examination, perinatal WNK1-deficient mice were significantly smaller than their *Csf1r*^*iCre–*^;*Wnk1*^*fl/fl*^ control littermates, exhibited smaller organ size, and lacked tooth eruption (Fig. 1b and Supplementary Fig. 1c–e). Further histopathological analysis revealed evidence of malformation and inflammation (Supplementary Fig. 1f). Upon observation of tissue disorganization and inflammatory infiltrates, we performed a complete pathology work-up to include liver enzymes, serum proteins, and circulating hematopoietic cell differentials, but found no significant differences in WNK1-deficient animals (Supplementary Fig. 1g and Supplementary Data 1), nor in red blood cell (RBC), platelet, monocyte, eosinophil, or basophil counts (Supplementary Fig. 1h and Supplementary Data 1). However, we did observe a significant increase in neutrophils and a decrease in lymphocytes (Supplementary Fig. 1g and Supplementary Data 1).

### Mice with *Csf1r*^iCre^-mediated deletion of WNK1 lack tissue-resident macrophages and develop neutrophilia

Given our findings that *Csf1r*^*iCre+*^;*Wnk1*^*fl/fl*^ (WNK1-deficient) mice exhibit decreased fecundity, smaller organ and body size, and premature death similar to *Csf1r*-deficient mice (i.e., 3–4 weeks old)^17^, we further queried whether WNK1-deficient mice present with differences in immune cell compartments. Flow cytometry and immunofluorescence (IF) analysis of immune cells within vital organs and tissues revealed TRM reduction or deficiency in all tissues except the brain (Fig. 1c and Supplementary Figs. 2a–c and 3a–c). The observed loss of TRMs in WNK1-deficient mice was accompanied by a dramatic increase in Ly6G+ neutrophil numbers across all tissues assessed (Fig. 1d and Supplementary Figs. 2d–h and 3a–c) on top of the observed increase in circulating neutrophils (Supplementary Fig. 1g; Supplementary Data 1). Detailed analysis of Ly6G+ neutrophils revealed that neutrophils from WNK1-deficient mice are morphologically and functionally similar to neutrophils from littermate controls (Supplementary Fig. 4a–c), suggesting that the increased neutrophilia in WNK1-deficient mice is not due to increased production of immature neutrophils. On the other hand, Ly6C+ monocyte numbers were either unaffected or modestly increased (spleen) across tissues analyzed via flow cytometry and in complete blood count analysis (Supplementary Figs. 1h, 2f–h; and Supplementary Data 1). Further analysis of monocyte subsets in WNK1-deficient mice revealed significantly decreased frequency of CX_3_CR1+ monocytes in the spleen, liver, and lung (Supplementary Fig. 2i, j), perhaps suggesting that WNK1 may be required for the development of monocytes via a specific precursor (e.g., via GMP, but not MDP^18^) which would account for the lack of significant differences in monocyte numbers.

Analysis of the lymphoid compartment revealed modest changes in some tissues (Supplementary Fig. 5a–c), which is consistent with our CBC analysis (Supplementary Data 1). The majority of CD4+ and CD8+ T cell numbers and frequencies remained similar across tissues analyzed, with only the spleen (increase in CD4+ T cells) and the lung (decrease in CD8+ T cells) showing significant changes (Supplementary Fig. 5b, c). We observed significantly decreased CD19+ B cell numbers and frequencies in nearly all tissues assessed (Supplementary Fig. 5a), consistent with the expression of CSF1R in the earliest fetal B cell progenitors which have both B cell and myeloid potential^19^, however this defect is unlikely to explain our observed phenotype because mice lacking B cells develop normally^20^. Taken together, our findings raise the hypothesis that mice bearing *Csf1r*^iCre^-mediated deletion of WNK1 suffer perinatally lethal defects in development due to failed TRM development.

### Mice with *Csf1r*^iCre^-mediated deletion of WNK1 present with evidence of disrupted embryonic myelopoiesis

One possible explanation for the absence of TRMs is that WNK1 supports their appropriate development and maturation initially from embryonic erythromyeloid progenitors (EMPs) and then from monocytes, as it was previously shown that *Csf1r* expression is observed in EMPs as early as embryonic day (E)7–8^21^ and in several hematopoietic populations, including monocytes^17,22^. To explore this possibility, we performed timed pregnancies and analyzed the yolk sac, heads, and trunks of E9.5 embryos of *Csf1r*^iCre/+^ x *Wnk1*^*fl/fl*^ (WNK1-deficient) and littermate control mice for the presence of EMPs, macrophages, neutrophils, and fetal monocytes by flow cytometry and in situ hybridization (Fig. 1e, f; Supplementary Figs. 6 and 7). We chose to analyze this time point because the TRMs arriving during this period derive from EMPs and are M-CSF/CSF1R-dependent^23^. As hypothesized, we observed significantly fewer CD11b^low^ F4/80+ TRMs within the trunk region (i.e., outside of the central nervous system) in E9.5 embryos of WNK1-deficient mice compared to littermate controls (Fig. 1e and Supplementary Fig. 6a–c), which persisted at E10.5 (Supplementary Fig. 7c–e) and E11.5 (Supplementary Fig. 7h, i). Consistent with our analysis of microglia in 4-week-old mice, we observed no significant differences in macrophage numbers between the yolk sac and head of WNK1-deficient mice and littermate controls (Fig. 1e and Supplementary Fig. 6a–c). On the other hand, we observed significantly increased numbers of CSF1R+ cKit+ AA4.1+ EMPs in the trunk and yolk sac, but not the head, of WNK1-deficient E9.5 embryos (Supplementary Fig. 6b–f). Interestingly, unlike macrophages, EMP numbers were similar at E10.5 (Supplementary Fig. 7c, f, g) and E11.5 (Supplementary Fig. 7j, k). Interestingly, all three regions of WNK1-deficient E9.5 embryos exhibited significantly more neutrophils than littermate controls (Fig. 1f and Supplementary Fig. 6d), similar to our observation of broad neutrophilia in WNK1-deficient neonates. Collectively, our data suggest that mice with *Csf1r*^iCre^-mediated deletion of WNK1 have defects in embryonic myeloid development.

The continued absence of TRMs and concomitant abundance of neutrophils in neonates suggests the possibility of disrupted myelopoiesis in the hematopoietic compartment. To address this, we characterized the hematopoietic stem cell and progenitor (HSCP) niche in the postnatal bone marrow of WNK1-deficient and littermate control mice (Supplementary Fig. 8). We observed no significant differences in the number of c-Kit^+^ Sca-1^+^ HSCs (Supplementary Fig. 8c). Upon analysis of HSPCs using CD48 and CD150 (SLAMF1) as classifiers of myelopoietic stage (Supplementary Fig. 8a), we observed a significant decrease in the number of long-term HSCs (LT; cKit^+^ Sca-1^+^ CD48^–^ CD150^+^), but not the number of short-term HSCs (ST; cKit^+^ Sca-1^+^ CD48^−^ CD150^–^) or multipotent progenitors (MPP; cKit^+^ Sca-1^+^ CD48^+^ CD150^–^; Supplementary Fig. 8e). We further analyzed MPP subsets first by delineating expression of Fms Related Receptor Tyrosine Kinase 3 (Flt3), CD150, and CD48 into early or late MPPs, and then into MPP2, MPP3, and MPP4 subsets, which give rise to erythrocyte/ megakaryocyte, granulocyte/ monocyte/ macrophage, and lymphoid lineages, respectively^24^ (Supplementary Fig. 8a). First, we found that WNK1-deficient neonates had normal numbers of Flt3^low^ (“early”) stage MPPs but significantly fewer Flt3^high^ (“late”) stage MPPs (Supplementary Fig. 8g). Second, we found significant differences in both MPP3 and MPP4 populations but not in MPP2s. Specifically, we observed significantly more MPP3s and fewer MPP4s in WNK1-deficient mice compared to littermate controls (Supplementary Fig. 8i). Our finding that WNK1-deficient neonates have more MPP3s, the subset that gives rise to the granulocyte-monocyte progenitor (GMP) under steady-state, is similar to our finding of increased EMP numbers in E9.5 WNK1-deficient embryos (Supplementary Fig. 6b–f). Nevertheless, our analysis of the neonatal hematopoietic compartment did not provide an obvious explanation for the continued absence of TRMs and neutrophil abundance in WNK1-deficient neonates.

### WNK1 absence or WNK kinase activity inhibition skews myelopoiesis towards neutropoiesis in response to M-CSF

**Fig. 2:**
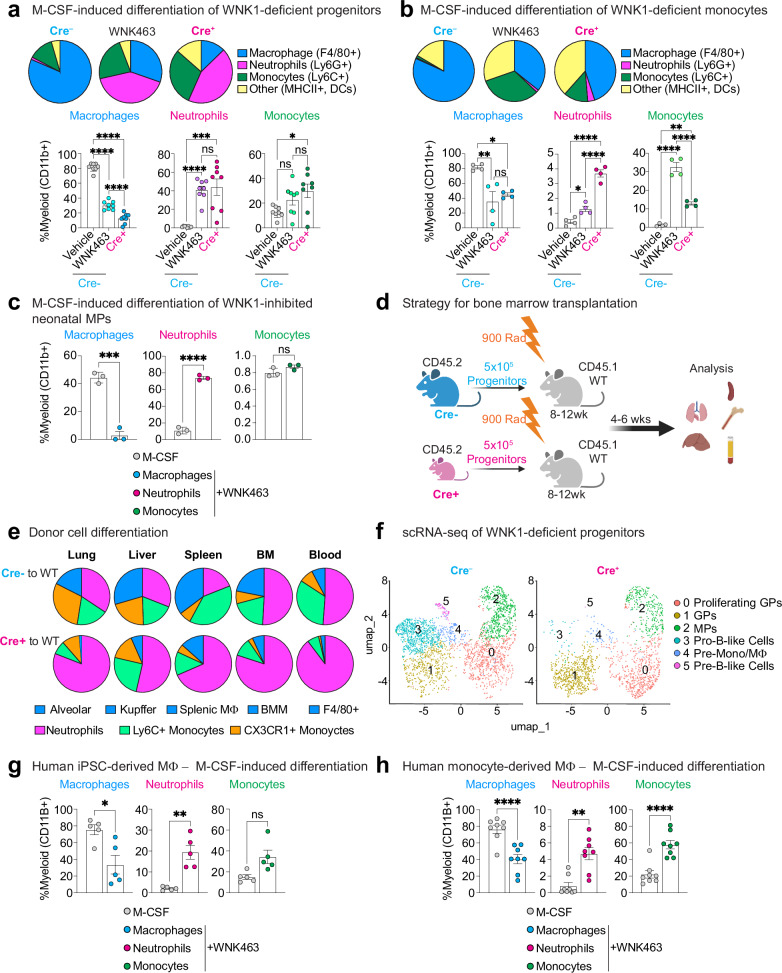
WNK1 links M-CSF signaling and macrophage differentiation. **a** Analysis of lineage frequency in M-CSF-treated mouse bone marrow myeloid progenitors. Bone marrow myeloid progenitors were isolated from *Csf1r*^*Cre+*^; *Wnk1*^*fl/fl*^ (KO, *n* = 8), *Csf1*^*Cre–*^*; Wnk1*^*fl/fl*^ treated with the WNK kinase activity inhibitor, WNK463 (+WNK463, *n* = 8), or *Csf1*^*Cre–*^*; Wnk1*^*fl/fl*^ (M-CSF, *n* = 8) mice and treated with M-CSF for 4 d. Cultures were subsequently analyzed for the presence of macrophages, monocytes, neutrophils, and dendritic cells (DCs). See Supplementary Fig. 2a for flow cytometry gating strategy. Live cells were first gated as CD45+ CD11b+. (Top) Shown are fraction of the whole pie charts of neutrophils (Ly6G+ Ly6C+ F4/80−; magenta), monocytes (Ly6G− F4/80− Ly6C+; green), macrophages (Ly6C− Ly6G− F4/80+; blue), and other/DCs (F4/80− MHC Class II+; yellow). (Bottom) Shown are summary graphs of the above-identified populations. Data are representative of three independent experiments. Summary plots are shown as mean ± SEM. Statistical significance was assessed via one-way ANOVA. *p < .05, ***p < .001, ****p < .0001, ns = not significant. **b** Analysis of lineage frequency in M-CSF-treated mouse bone marrow Ly6C+ monocytes. Bone marrow Ly6C+ monocytes were isolated from *Csf1r*^*Cre+*^*; Wnk1*^*fl/fl*^ (KO, *n* = 3), *Csf1*^*Cre–*^*; Wnk1*^*fl/fl*^ treated with WNK463 (+WNK463, *n* = 4), or *Csf1*^*Cre–*^*; Wnk1*^*fl/fl*^ (M-CSF, *n* = 4) mice and treated with M-CSF for 4 d. Cultures were subsequently analyzed for the presence of macrophages, monocytes, neutrophils, and dendritic cells (DCs). See Supplementary Fig. 2a for flow cytometry gating strategy. Live cells were first gated as CD45+ CD11b+. (Top) Shown are fraction of the whole pie charts of neutrophils (Ly6G+ Ly6C+ F4/80−; magenta), monocytes (Ly6G− F4/80− Ly6C+; green), macrophages (Ly6C- Ly6G− F4/80+; blue), and other/DCs (F4/80− MHC Class II+; yellow). (Bottom) Shown are summary graphs of the above-identified populations. Data are representative of three independent experiments. Summary plots are shown as mean ± SEM. Statistical significance was assessed via one-way ANOVA. *p < .05, **p < .01, ***p < .001, ****p < .0001. **c** Analysis of lineage frequency in M-CSF-treated mouse neonatal myeloid progenitors. Wildtype mouse neonatal myeloid progenitors (MPs) were treated with M-CSF in the presence of vehicle or WNK463 for 3 d. Cultures were subsequently analyzed for the presence of macrophages, monocytes, and neutrophils. See Supplementary Fig. 2a for flow cytometry gating strategy. Live cells were first gated as CD45+ CD11b+, then gated for neutrophils (Ly6G+ Ly6C+ F4/80−; magenta), monocytes (Ly6G− F4/80− Ly6C+; green), and macrophages (Ly6C− Ly6G− F4/80+; blue). Shown are summary graphs of the above-identified populations. Data are representative of four independent experiments. Summary plots are shown as mean ± SEM. Statistical significance was assessed via two-tailed independent samples *t*-test. ***p < .001, ****p < .0001, ns = not significant. **d**, **e** Transplantation of Cre- or Cre+ bone marrow myeloid progenitors into adult wildtype hosts. **d** Schematic of experimental strategy. Bone marrow myeloid progenitors from Cre− to Cre+ mice were transplanted into congenically labeled, lethally irradiated (900 rad) adult wildtype (WT) recipient mice, then analyzed 4–6 weeks post-transplant. **e** Shown is the frequency of macrophages (blue), neutrophils (magenta), Ly6C+ monocytes (green), and CX_3_CR1+ monocytes (orange) arising from Cre− (Cre− to WT) and Cre+ (Cre+ to WT) donor progenitors across tissues analyzed. See Supplementary Fig. 2a for flow cytometry gating strategy. Data are from two independent experiments with six (Cre+) or four (Cre−) mice per group. **f** Single cell RNA sequencing analysis of multipotent progenitor (MPP)3 cells. MPP3 cells (Lin− cKit+ Sca-1+ Flt3− CD150− CD48+) were flow cytometry sorted from *Csf1*^*Cre–*^*; Wnk1*^*fl/fl*^ (Cre-, *n* = 3) and *Csf1r*^*Cre+*^*; Wnk1*^*fl/fl*^ (Cre+, *n* = 4) neonatal bone marrow, labeled with barcoded MHC Class I-specific antibodies, and processed for scRNAseq. Shown is the dimension reduction analysis of MPP3 cells from Cre− mice using UMAP with overlayed analysis of Cre+ MPP3s. **g** Perturbation of WNK kinase activity prevents differentiation of human myeloid progenitors into mature macrophages. Shown are summary plots of myeloid subsets from experiments performed as detailed in Supplementary Fig. 11a using human pluripotent stem cell-derived myeloid progenitors. Pooled data are from four independent experiments with one-to-two biological replicates (unique donors; *n* = 5 per group) per experiment. Data are shown as mean ± SEM. Statistical significance was determined via two-tailed independent samples *t*-test. *p < .05, **p < .01, ns = not significant. See also Supplementary Fig. 12a for flow cytometry characterization of cell-surface proteins. **h** Perturbation of WNK kinase activity blocks differentiation of human monocytes into mature macrophages. Shown are summary plots of myeloid subsets from experiments performed as detailed in Supplementary Fig. 11g using human blood monocytes. Pooled data are from four independent experiments with two biological replicates (unique male and female donors) per experiment. Data are shown as mean ± SEM. Statistical significance was determined via two-tailed independent samples *t*-test. **p < .01, ***p < .001. See also Supplementary Fig. 12b for flow cytometry characterization of cell-surface proteins. Source data are provided in a Source Data file included with this manuscript. Created in BioRender. Perry, J. (2025) https://BioRender.com/mxpjzhx.

Macrophage colony-stimulating factor (M-CSF) is a cytokine critical for myeloid progenitor and precursor (i.e., monocyte) cell differentiation into macrophages instead of other immune cell lineages^25^. Because our finding that WNK1-deficient mice lack TRMs but have relatively undisturbed monocyte and hematopoietic progenitor compartments is reminiscent of CSF1R-deficient mice, we next tested if macrophage differentiation in response to M-CSF is affected in *Csf1r*^*iCre+*^*; Wnk1*^*fl/fl*^ (WNK1-deficient) mice. To this end, we cultured bone marrow lineage-negative (Lin-) progenitors or bone marrow monocytes in vitro in the presence of M-CSF (Supplementary Fig. 9a). Remarkably, progenitors and monocytes cultured from WNK1-deficient mice were almost completely devoid of conventional mature macrophages (CD45+ CD11b+ F4/80+; Fig. 2a, b and Supplementary Fig. 9b–e). Interestingly, WNK1-deficient progenitors treated with M-CSF exhibited a predominantly neutrophilic phenotype (CD45+ CD11b+ Ly6C+ Ly6G+; Fig. 2a). Furthermore, we observed significantly increased monocyte frequencies (CD45+ CD11b+ Ly6G− Ly6C+) in cultures using monocytes (Fig. 2b) but not progenitors (Fig. 2a). Surprisingly, there was a significant emergence of neutrophils from WNK1-deficient monocytes (Fig. 2b), albeit at a much lower rate than observed when using progenitors (Fig. 2a). Although we confirmed the absence of bone marrow neutrophils from monocyte isolations, we cannot rule out that this emergence is due to the presence of pre-monocytes that retain neutrophil differentiation potential.

We next tested whether macrophage differentiation in response to M-CSF could be blocked in otherwise healthy cells. Indeed, addition of the pan-WNK kinase activity inhibitor, WNK463^26–28^, to wildtype Lin- progenitors or monocytes cultured with M-CSF resulted in significantly decreased macrophage differentiation (Fig. 2a, b) and significantly increased neutrophil differentiation from progenitors (Fig. 2a). Furthermore, addition of WNK463 to mouse neonatal myeloid progenitors cultured with M-CSF completely blocked macrophage differentiation, instead inducing significant neutrophil differentiation (Fig. 2c). Thus, our in vitro data suggest that the striking in vivo phenotype of TRM absence and neutrophilia is explained by myeloid progenitor and monocyte dependence on WNK1 for appropriate lineage commitment in response to M-CSF.

Finally, we sought to directly test the hypothesis that WNK1-deficient Lin- progenitors primarily differentiate into neutrophils instead of macrophages in vivo. To this end, we transferred Lin-bone marrow progenitors from WNK1-deficient or littermate control mice into irradiated adult congenic CD45.1 C57BL/6J mice (Fig. 2d). Recipient mice tissues were subsequently analyzed for the presence of donor macrophages, neutrophils, and monocytes. Consistent with our in vitro findings, progenitors from WNK1-deficient mice failed to develop into TRMs, instead predominantly differentiating into neutrophils (Fig. 2e and Supplementary Fig. 10a–d). Our findings suggest that, in addition to the failed differentiation of progenitors and monocytes into macrophages, WNK1-deficient mice develop neutrophilia because of enhanced differentiation of myeloid progenitors into neutrophils. Consistent with this, single cell RNA sequencing of MPP3s from WNK1-deficient and littermate control mice revealed only a modest decrease in *Cd48*-expressing “monocyte precursor” cells (MPs)^29^ that preferentially give rise to monocytes (Cluster 2; Fig. 2f and Supplementary Data 2) but a significant increase in *Cd63*-expressing “granulocyte precursor” cells (GPs)^30^ that preferentially give rise to neutrophils (Cluster 0 and 1; Fig. 2f and Supplementary Data 2). Collectively, these data support that the pathological consequence of myeloid-specific WNK1 absence is due to cell-intrinsic TRM development failure and not to cell-extrinsic defects in the tissue microenvironment.

### WNK kinase activity is required for human macrophage differentiation

We next investigated whether the dependence on WNK1 for M-CSF-induced differentiation of mouse myeloid progenitors and monocytes into conventional macrophages is conserved in humans. To this end, we treated either human induced pluripotent stem cell (iPSC)-derived progenitors or human peripheral blood CD14^high^ circulating monocytes with M-CSF in the presence of vehicle or WNK463. First, we grew iPSC-derived embryoid bodies (EBs) in a defined media, which first differentiates EBs into myeloid precursors (via an M-CSF-dependent step), then terminally differentiates myeloid progenitors into mature myeloid or granulocyte subsets (Supplementary Fig. 11a), including mature macrophages^31^. Interestingly, we initially observed decreased M-CSF-dependent differentiation of EBs into myeloid precursors when treated with WNK463 (Supplementary Fig. 11c, d). As a complementary approach, we first progressed EBs to myeloid precursors prior to the addition of WNK463. Similar to our results using mouse bone marrow progenitors and mouse embryonic EMPs, we found that treatment of human myeloid precursors with WNK463 resulted in significantly fewer macrophages (Fig. 2g and Supplementary Fig. 11e, f, j) and reduced expression of conventional macrophage markers (Supplementary Fig. 12a) but increased neutrophil numbers (Fig. 2g and Supplementary Fig. 11j) in response to M-CSF.

Next, we found that peripheral blood CD14^high^ circulating monocytes cultured with M-CSF robustly (Supplementary Fig. 11g) produced morphologically and phenotypically conventional (CSF1R+) macrophages (Fig. 2h and Supplementary Figs. 11b and 12b). However, in the presence of WNK463, monocytes failed to differentiate into conventional macrophages (Fig. 2h and Supplementary Figs. 11h, i, and 12b). It is worth noting that CD206 was significantly decreased (Supplementary Fig. 12b). This finding is synonymous with our observation with mouse bone marrow progenitors (Supplementary Fig. 9a), suggesting that expression of CD206, a protein associated with canonical anti-inflammatory responses, may require WNK1^12^. Nevertheless, our findings demonstrate that the dependence on WNK kinase activity for M-CSF-induced progenitors and monocyte differentiation into mature macrophages is conserved between mice and humans.

### Adoptive transfer of bone marrow progenitors or monocytes rescues mortality in mice with *Csf1r*^iCre^-mediated deletion of WNK1

TRMs, to some extent or another, can be repopulated by monocytes either endogenously or via adoptive transfer into neonatal mice^32^. Despite having relatively normal monocyte numbers, tissues from *Csf1r*^*Cre+*^; *Wnk1*^*fl/fl*^ (WNK1-deficient) mice still lack TRMs. Although this suggests that WNK1 is functioning downstream of CSF1R signaling in a cell-autonomous manner, our findings may indicate disrupted tissue microenvironments that do not favor macrophage differentiation and maturation. To test this alternative hypothesis, we injected either wildtype progenitors or wildtype monocytes into WNK1-deficient mice and littermate controls beginning at postnatal day (P)2 (Supplementary Fig. 13a). Injection of congenically-labeled wildtype progenitors or monocytes rescued mortality of WNK1-deficient mice (Supplementary Figs. 13b and 14a). Consistent with these observations, flow cytometric analysis of key organs and tissues revealed significant reconstitution of the tissue macrophage pools by congenically-labeled wildtype progenitors (Supplementary Fig. 13c, d) and monocytes (Supplementary Fig. 14b). We next queried if progenitor or monocyte transfer also affected the neutrophilia observed in WNK1-deficient mice. Most tissues and organs analyzed still exhibited significant neutrophilia, including the lungs and liver (Supplementary Figs. 13e–g and 14c–e), suggesting that TRM absence, and not neutrophilia per se, is the primary contributor to the mortality observed in WNK1-deficient mice. These independent experiments demonstrate, at least in early perinates, that tissues in WNK1-deficient mice (e.g., the bone marrow niche) remain capable of supporting macrophage development. Taken together, our findings suggest that mice bearing CSF1R^Cre^-specific deletion of WNK1 suffer perinatal-lethal defects in development due to failed TRM development.

### M-CSF stimulates macropinocytosis in mouse and human progenitors in a WNK1-/WNK kinase signaling-dependent manner

**Fig. 3:**
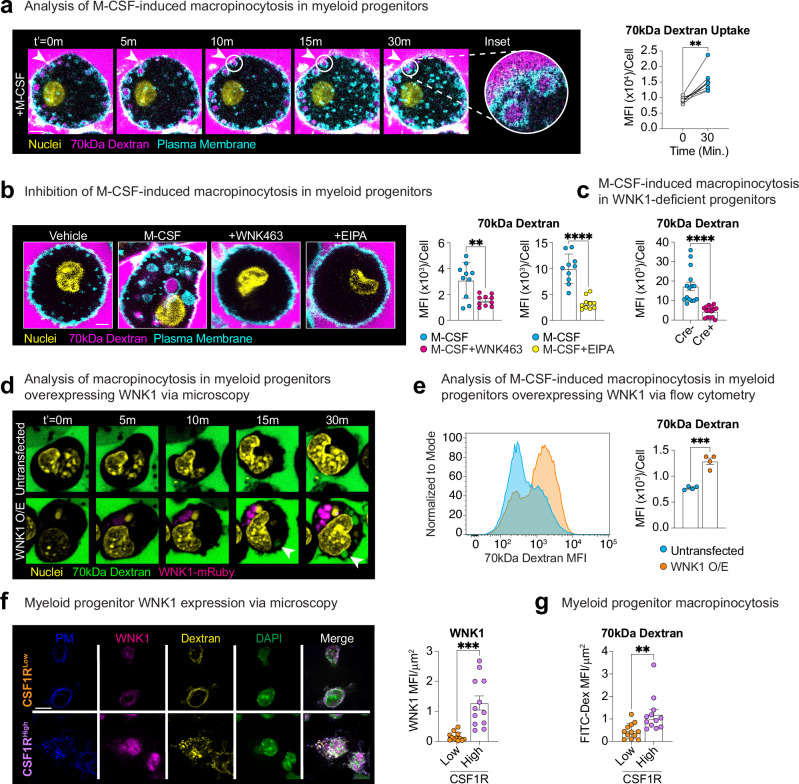
M-CSF stimulates macropinocytosis in myeloid progenitors in a WNK kinase activity-dependent manner. **a** Myeloid progenitors perform macropinocytosis in response to M-CSF. (Left) Representative images of myeloid progenitors incubated with M-CSF and 70kDa-florescent Dextran (magenta). Nuclei were stained with Hoechst (yellow) and the plasma membrane was labeled using CellMask (cyan), then myeloid progenitors were imaged at 100X magnification for 30 min. Scale bar, 2 μm. (Right) Quantitation of 70 kDa Dextran uptake per cell via geometric mean fluorescence intensity (MFI) at *t* = 0 and 30 min. Data is representative of four independent experiments, *n* = 8 per time point. Statistical significance was determined via two-tailed paired samples *t*-test. **p < .01. **b** Inhibition of WNK kinase activity or macropinocytosis blocks M-CSF-stimulated internalization of 70 kDa Dextran by myeloid progenitors. Experiments were performed as in (**a**) using myeloid progenitors. (Left) Representative images of myeloid progenitors treated with M-CSF and either WNK463 or EIPA. Scale bar, 2 μm. (Right) Quantitation of 70 kDa Dextran uptake per cell via MFI after 30 min. Data are from four independent experiments. Each dot represents the average MFI per field of view (*n* = 10 per group). Data are shown as mean ± SEM. Statistical significance was determined via two-tailed independent samples *t*-test. **p < .01, ****p < .0001. **c** M-CSF-stimulated internalization of 70 kDa Dextran is lost in WNK1-deficient myeloid progenitors. Experiments were performed as in (**a**) using bone marrow myeloid progenitors from *Csf1r*^*Cre+*^*; Wnk1*^*fl/fl*^ (Cre+) or *Csf1*^*Cre–*^*; Wnk1*^*fl/fl*^ (Cre−) mice. Shown is the quantitation of 70 kDa Dextran uptake via MFI per cell. Data is from four independent experiments with two-three mice (biological replicates) per experiment (n = 5 per condition). Statistical significance was determined via two-tailed independent samples *t*-test. ****p < .0001. **d**, **e** WNK1 boosts M-CSF-stimulated macropinocytosis in myeloid progenitors. Experiments were performed as in (**a**) using bone marrow myeloid progenitors overexpressing WNK1-mRuby. Shown are confocal microscopy (**d**) and flow cytometry (**e**) analysis of 70 kDa Dextran uptake. Data is from four independent experiments. Data are shown as mean ± SEM. Statistical significance was determined via two-tailed independent samples *t*-test. ***p < .001. **f**, **g** Confocal microscopy analysis of WNK1 protein expression and macropinocytosis in M-CSF-treated granulocyte and monocyte progenitors. **f** (Left) Representative fixed images of granulocyte progenitors (GPs, orange) sorted from WT animals based on Lin− cKit+ Sca-1− CD34+ CD64^High^ Ly6C+ CSF1R^Low^ or macrophage/monocyte progenitors (MPs, purple) sorted based on Lin− cKit+ Sca-1− CD34+ CD64^High^ Ly6C+ CSF1R^High^. See Supplementary Fig. 2a for flow cytometry gating strategy. Cells were stained with WNK1 antibody (magenta), plasma membrane dye (PM, blue), DAPI (green), and treated with 1 mg/mL 70 kDa Dextran (yellow), and 100 ng/mL M-CSF for 1 h. Scale bar, 5 μm (Right) Quantitation of WNK1 protein expression and 70 kDa Dextran uptake (**g**) via MFI per field view area (FOV = μm^2^). Data are from three independent experiments. *n* = 12 FOVs per group. Data are shown as mean ± SEM. Statistical significance was determined via two-tailed independent samples *t*-test. **p < .01, ***p < .001. Source data are provided in a Source Data file included with this manuscript.

Stimulation of mature macrophages with M-CSF triggers macropinocytosis, an engulfment process involving “cell drinking”, whose physiologic functions in metazoans are only recently emerging^15,33–35^. TRMs use M-CSF-induced macropinocytosis as a means of sensing their surroundings, but research on this type of uptake is limited to terminally-differentiated cells^14,34,36^. Despite the known M-CSF dependence for macrophage differentiation, there is no evidence that myeloid progenitors or monocytes perform macropinocytosis in response to M-CSF or that macropinocytosis is required for macrophage lineage commitment. To directly test this, we established a protocol to perform high-resolution time-lapse confocal microscopy analysis of macropinocytosis by different myeloid progenitor types (Supplementary Fig. 15a). Additionally, we analyzed uptake of two different macropinocytosis substrates, one that is pH-sensitive (DQ-Red bovine serum albumin^12,37^) and one that is pH-insensitive (70 kDa Dextran), to ensure specificity of the biological process. Indeed, myeloid progenitors performed macropinocytosis in response to M-CSF (Fig. 3a). Furthermore, treatment with the macropinocytosis-selective inhibitor EIPA^38–41^ (Supplementary Fig. 15b) significantly decreased M-CSF-stimulated continuous macropinocytosis across myeloid progenitor types and substrates tested (Fig. 3b and Supplementary Figs. 15c–f and 16a–d). Importantly, both inhibition of WNK kinase activity (Fig. 3b and Supplementary Figs. 15d, e and 16a–d) and absence of WNK1 protein (Fig. 3c and Supplementary Fig. 15f) also significantly inhibited M-CSF-stimulated continuous macropinocytosis in both mouse and human myeloid progenitors. Finally, treatment with LY294002, which disrupts macropinosome formation via inhibition of phosphatidylinositol 3-kinase (PI3K)^42^, or Apilimod, which prevents macropinosome vacuole maturation via inhibition of Phosphoinositide Kinase, FYVE-Type Zinc Finger Containing (PIKfyve^43^; Supplementary Fig. 15b) recapitulated the macropinocytosis inhibition observed with EIPA and WNK463 (Supplementary Fig. 15g, h).

Next, we sought to determine if WNK1 directly regulates M-CSF-stimulated macropinocytosis. To this end, we overexpressed a functional WNK1-mRuby fusion protein^44^ into hematopoietic stem and progenitor cells (Fig. 3d). Overexpression of WNK1 resulted in more rapid and significantly more total macropinocytotic uptake on a per cell basis (Fig. 3d, e). This finding is particularly relevant because during hematopoiesis, expression of CSF1R is a key factor delineating early monocyte/macrophage (CSF1R^high^) and granulocyte (CSF1R^low^) progenitors^45^, which we find also significantly delineates high versus low expression of WNK1 (Fig. 3f). Interestingly, progenitors with high CSF1R/WNK1 expression perform significantly more M-CSF-stimulated macropinocytosis than progenitors with low CSF1R/WNK1 expression (Fig. 3g). Taken together, our data suggest that M-CSF induces continuous macropinocytosis in mouse and human myeloid progenitors and that WNK1/WNK kinase activity directly regulates M-CSF-stimulated continuous macropinocytosis.

### Macropinocytosis supports appropriate myeloid lineage commitment

**Fig. 4:**
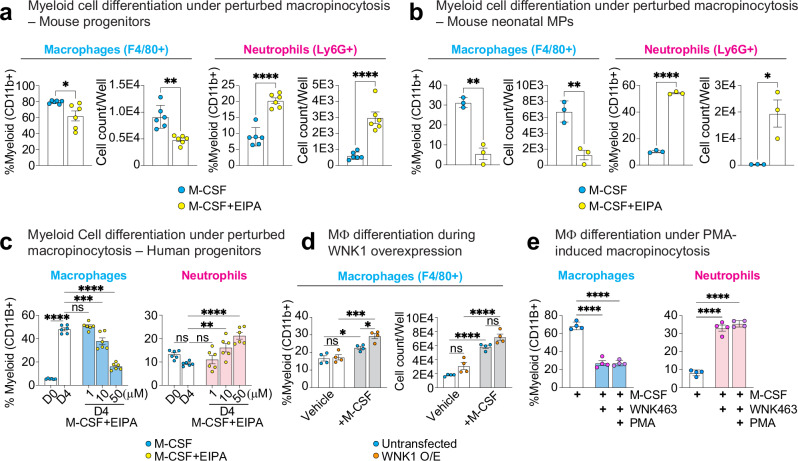
M-CSF stimulates macropinocytosis in myeloid progenitors in a WNK kinase activity-dependent manner. **a** Perturbation of macropinocytosis skews M-CSF-stimulated bone marrow myeloid progenitor differentiation from monocytes/macrophages to neutrophils. Bone marrow myeloid progenitors were isolated and differentiated with M-CSF in the presence of vehicle or EIPA for 4 d. See Supplementary Fig. 2a for flow cytometry gating strategy. Shown are summary plots of flow cytometry analysis for macrophages (F4/80+) and neutrophils (Ly6G+). Data are presented as a frequency of myeloid cells (CD11b+, left graphs) or total cell counts per well (right graphs) from three independent experiments with two mice per experiment. Data are shown as mean ± SEM. Statistical significance was determined via two-tailed independent samples *t*-test. *p < .05, **p < .01, ****p < .0001. See also Supplementary Fig. 16a for cell viability analysis. **b** Perturbation of macropinocytosis skews M-CSF-stimulated mouse neonatal myeloid progenitor differentiation from macrophages to neutrophils. Mouse embryonic stem cell-derived myeloid progenitors were differentiated with M-CSF in the presence of vehicle or EIPA for 4 d. Shown are summary plots of flow cytometry analysis for macrophages (F4/80+) and neutrophils (Ly6G+). Data are presented as a frequency of myeloid cells (CD11b+, left graphs) or total cell counts per well (right graphs) from three independent experiments. See Supplementary Fig. 2a for flow cytometry gating strategy. Data are shown as mean ± SEM. Statistical significance was determined via two-tailed independent samples *t*-test. *p < .05, **p < .01, ****p < .0001. See also Supplementary Fig. 16b for cell viability analysis. **c** Perturbation of macropinocytosis skews M-CSF-stimulated human myeloid progenitor differentiation from macrophages to neutrophils in a dose-dependent manner. Human myeloid progenitors were differentiated with M-CSF in the presence of vehicle or EIPA at indicated concentrations for 4 d. Shown are summary plots of flow cytometry analysis for macrophages (CD11B+, CD14^Low^, CD16^High^, CSF1R+, CD206+, and CD86+) and neutrophils (CD11B+, CD14^Low^, CD15+, and CD66B+). See Supplementary Fig. 2a for flow cytometry gating strategy. Data are presented as a frequency of myeloid cells (CD11B+) from three independent experiments (*n* = 6 per condition). Data are shown as mean ± SEM. Statistical significance was determined via one-way ANOVA. **p < .01, ***p < .001, ****p < .0001, ns = not significant. See also Supplementary Fig. 16c, d for cell viability analysis. **d** WNK1 overexpression enhances differentiation of myeloid progenitors in response to M-CSF. Experiments were performed as in (**a**), but with wildtype cells overexpressing functional WNK1-mRuby (see Fig. 3d, e for functional analysis) and analysis performed after 24 h. Shown are summary plots of flow cytometry analysis for macrophages (F4/80 + ). Data are presented as a frequency of myeloid cells (CD11b+, left graphs) or total cell counts per well (right graphs) from four independent experiments. See Supplementary Fig. 2a for flow cytometry gating strategy. Data are shown as mean ± SEM. Statistical significance was determined via two-tailed independent samples *t*-test. *p < .05, ***p < .001, ****p < .0001. **e** PMA-induced macropinocytosis does not correct aberrant differentiation in M-CSF-stimulated myeloid progenitors treated with WNK463. Experiments were performed as in (c), but with the inclusion of PMA treatment, which induces WNK kinase activity-independent macropinocytosis (Supplementary Fig. 16e, f). Shown are summary plots of flow cytometry analysis for macrophages (CD11B+, CD14^Low^, CD16^High^, CSF1R+, CD206+, and CD86+) and neutrophils (CD11B+, CD14^Low^, CD15+, and CD66B+). Data are presented as a frequency of myeloid cells (CD11B+) from four independent experiments. Data are shown as mean ± SEM. Statistical significance was determined via one-way ANOVA. ****p < .0001. Source data are provided in a Source Data file included with this manuscript.

Building on our finding that M-CSF stimulates macropinocytosis by both mouse and human progenitors in a WNK1/WNK kinase activity-dependent manner, we sought to directly test the hypothesis that M-CSF-stimulated macropinocytosis is necessary for differentiation of myeloid progenitors and monocytes into macrophages. Indeed, inhibition of macropinocytosis via EIPA resulted in a significant decrease in macrophage differentiation (F4/80+) and a concomitant significant increase in neutrophil generation (Ly6G+) when using either bone marrow myeloid progenitors where macrophages develop from a monocyte intermediate (Fig. 4a) or mouse neonatal myeloid progenitors which directly differentiate into macrophages (Fig. 4b). Additionally, disruption of macropinocytosis in human progenitors led to a significant decrease in macrophage differentiation and a significant increase in neutrophil generation (Fig. 4c).

Although we demonstrate that M-CSF-stimulated continuous macropinocytosis by myeloid progenitors requires WNK1/WNK kinase activity, whether WNK1/WNK kinase activity is directly required for M-CSF-induced differentiation or supports differentiation indirectly via its regulation of macropinocytosis is unknown. To test this, we took two approaches. First, we exploited our finding that WNK1 overexpression boosts M-CSF-stimulated macropinocytosis in myeloid progenitors (Fig. 3d, e) to test if increased WNK1-dependent macropinocytosis also increases macrophage differentiation. Strikingly, by analyzing macrophage differentiation at an earlier time point (24 h), we found that WNK1 overexpression enhanced macrophage differentiation in response to M-CSF (Fig. 4d). Second, we took advantage of our observation that treatment with phorbol 12-myristate 13-acetate (PMA), which induces growth factor-independent macropinocytosis^46^, stimulates macropinocytosis in mouse and human myeloid progenitors with WNK kinase activity inhibited (Supplementary Fig. 16e, f) to test if WNK kinase activity is itself necessary. Strikingly, PMA, despite rescuing macropinocytosis in M-CSF-stimulated/WNK kinase activity inhibited myeloid progenitors, was unable to rescue either failed macrophage differentiation or enhanced neutrophil differentiation (Fig. 4e). Collectively, our results suggest that differentiation of mouse and human myeloid progenitors requires M-CSF-stimulated macropinocytosis, which in turn requires WNK1/WNK kinase activity beyond its role in regulating M-CSF-stimulated continuous macropinocytosis.

### WNK1/WNK kinase activity links M-CSF signaling, macropinocytosis, and macrophage differentiation

**Fig. 5:**
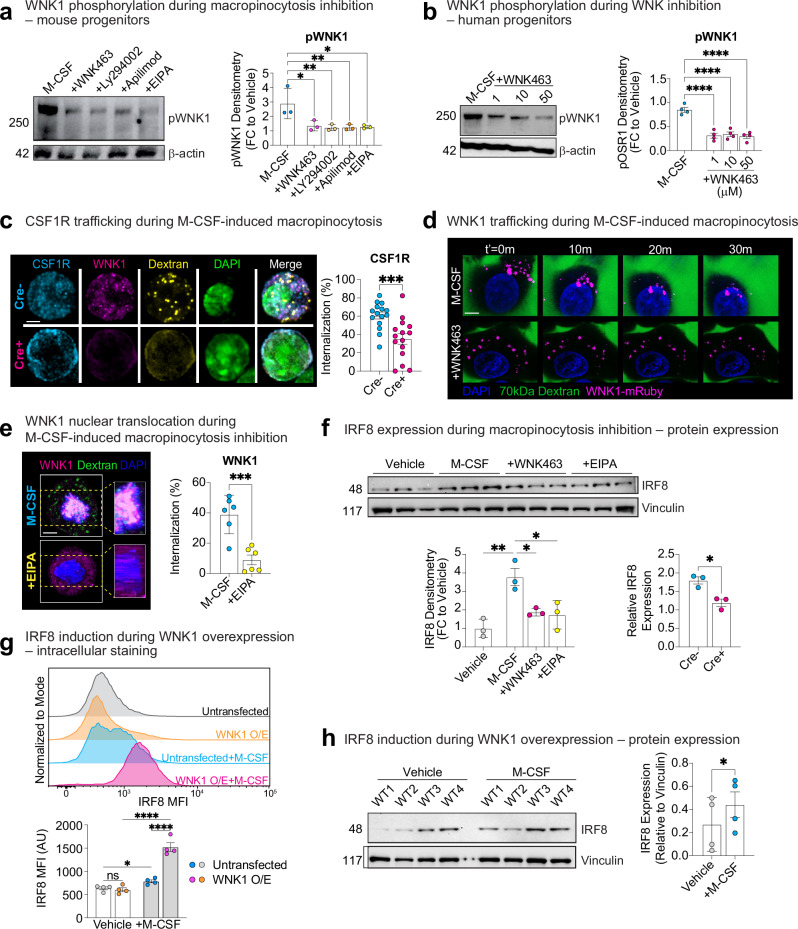
Macropinocytosis induces CSF1R internalization in a WNK1-dependent manner. **a** M-CSF-stimulated macropinocytosis induces phosphorylation of the WNK1 kinase region in bone marrow myeloid progenitors. Mouse myeloid progenitors were isolated from the bone marrow and stimulated with M-CSF in the presence of vehicle, WNK463, or indicated macropinocytosis inhibitors EIPA, Apilimod, and LY294002. Cells were harvested after 1 h, then assayed for WNK1 phosphorylation. Shown is a representative Phos-tag western blot (left) and quantitation of WNK1 phosphorylation (pWNK1; right). pWNK1 expression was normalized to loading control (β-actin, 42 kDa) and then calculated as a fold change (FC) of the pWNK1 bands (250 kDa) to a vehicle only (no M-CSF) control. Data are from two independent experiments with two biological replicates per condition/experiment. Data are shown as mean ± SEM. Statistical significance was determined via one-way ANOVA. *p < .05, **p < .01. **b** M-CSF-stimulated macropinocytosis induces phosphorylation of the WNK1 kinase region in human myeloid progenitors. Human iPSC-derived myeloid progenitors were stimulated with M-CSF in the presence of vehicle or WNK463 at the indicated concentrations, then assayed for WNK1 phosphorylation after 1 h. Shown is a representative Phos-tag western blot (left) and quantitation of WNK1 phosphorylation (pWNK1; right). pWNK1 levels were calculated and present as in (**a**). Data are pooled from two independent experiments with two biological replicates per condition/experiment. Data are shown as mean ± SEM. Statistical significance was determined via one-way ANOVA. **p < .01, ****p < .0001. **c** CSF1R is internalized in response to M-CSF stimulation in mouse myeloid progenitors. (Left) Representative images of bone marrow myeloid progenitors from *Csf1r*^*Cre+*^*; Wnk1*^*fl/fl*^ (Cre+) or *Csf1*^*Cre–*^*; Wnk1*^*fl/fl*^ (Cre−) were incubated with 70 kDa Dextran (yellow) and M-CSF for 1 h. Then, cells were fixed and labeled with CSF1R (cyan), WNK1 (magenta), and DAPI (green). Scale bar, 5 μm. (Right) Quantitation of CSF1R protein internalization. Each dot represents the average MFI per field of view (FOV) divided by the number of cells per region, with n = 15 FOVs per group. Scale bar, 5 μm. The data shown is representative of four independent experiments. Data are shown as mean ± SEM. Statistical significance was determined via two-tailed independent samples *t*-test. ***p < .001, ns = not significant. **d**, **e** WNK1 translocates into the nucleus in response to M-CSF-stimulated macropinocytosis in a WNK kinase activity-dependent manner. WNK1 nuclear translocation analyzed via time-lapse confocal microscopy of WNK1-mRuby-expressing myeloid progenitors (**d**) or fixed-image confocal microscopy with a validated WNK1 antibody (**e**). In **d**, WNK1-mRuby-expressing myeloid progenitors were incubated with 70 kDa Dextran and M-CSF in the presence of vehicle or WNK463 and imaged over 1 h. In **e**, wildtype myeloid progenitors were incubated with 70 kDa Dextran and M-CSF in the presence of vehicle or EIPA for 1 h prior to fixation and analysis. Shown is a representative image (left) and summary plot (right) of WNK1 nuclear translocation. Quantitation was performed on 3D projections of each cell using Manders’ Overlap Coefficient (MCC%) with DAPI. Each dot represents the WNK1/DAPI MCC% overlap per cell, with *n* = 6 FOVs per group. Scale bar, 5 μm. Data are from four independent experiments. Data are shown as mean ± SEM. Statistical significance was determined via two-tailed independent samples *t*-test. ***p < .001. **f** M-CSF-stimulated macropinocytosis induces a WNK kinase activity-dependent increase in IRF8 protein expression in myeloid progenitors. Mouse bone marrow myeloid progenitors were stimulated with M-CSF in the presence of vehicle, WNK463, or EIPA. Cells were harvested after 24 h, then assayed via western blot for IRF8 protein expression. Shown is a representative western blot (left) and quantitation of IRF8 expression (right). IRF8 expression was normalized to loading control (Vinculin, 117 kDa) and then calculated as a fold change (FC) of the IRF8 band (48kD) to a vehicle only (no M-CSF) control. Data are from three independent experiments. Data are shown as mean ± SEM. Statistical significance was determined via one-way ANOVA. *p < .05, **p < .01. **g**, **h** WNK1 overexpression enhances IRF8 protein expression in response to M-CSF. Experiments were performed as in (**f**), but with wildtype cells overexpressing functional WNK1-mRuby (see Fig. 3d, e for functional analysis). IRF8 protein expression was assessed using orthogonal methods to improve sensitivity of detection: via intracellular flow cytometry (**g**) or via western blot (**h**). In **g**, shown are representative images (left) and quantitation of the geometric mean fluorescence intensity of IRF8 (right). In **h**, shown is a representative western blot (left) and quantitation of IRF8 expression (right). IRF8 expression was normalized to loading control (Vinculin, 117 kDa) and then calculated as a fold change (FC) of the IRF8 band (48 kDa) to a vehicle-only (no M-CSF) control. Data are from four independent experiments. Data are shown as mean ± SEM. Statistical significance was determined via one-way ANOVA (**g**) or two-tailed independent samples *t*-test (**h**). *p < .05, ****p < .0001, ns = not significant. Source data are provided in a Source Data file included with this manuscript.

Next, we aimed to delineate how WNK1/WNK kinase activity directly informs myeloid lineage specification in response to M-CSF-stimulated macropinocytosis. Previous work has shown that downstream WNK1 function requires its phosphorylation, resulting in its activation and subsequent phosphorylation of the effector kinases OSR1 and SPAK^47,48^. Indeed, addition of M-CSF resulted in rapid (1 h) phosphorylation of WNK1 in its known chloride-sensing kinase domain in both mouse (Fig. 5a) and human (Fig. 5b) myeloid progenitors. Surprisingly, however, M-CSF treatment only induced phosphorylation of OSR1 but not SPAK (Supplementary Fig. 17a, b), suggesting that WNK1 is functioning in a non-conventional manner^49,50^. Nevertheless, treatment with EIPA, LY294002, or Apilimod all blocked M-CSF-induced WNK1 phosphorylation (Fig. 5a), confirming that WNK1 phosphorylation is specifically induced by M-CSF-stimulated macropinocytosis.

Recent work suggests that WNK1, beyond its established role in cell volume regulation, may also function directly in transcription regulation^50–54^, including by nuclear localization^49^. To investigate this, we performed both confocal microscopy of fixed CSF1R+ myeloid progenitors using a validated WNK1 antibody (Fig. 5c and Supplementary Fig. 17c) and time-lapse confocal microscopy of WNK1-mRuby-expressing myeloid progenitors exposed to M-CSF. Interestingly, CSF1R internalization, which occurs in mature macrophages in response to M-CSF^55,56^ and may provide a unique signaling platform^57^, was significantly decreased in the absence of WNK1 or when WNK kinase activity was inhibited (Fig. 5c and Supplementary Fig. 17d). Excitingly, we found that M-CSF induces translocation of WNK1 to the nucleus concomitant with CSF1R internalization, which was prevented when WNK kinase activity or macropinocytosis was inhibited (Fig. 5d, e and Supplementary Fig. 17e). Furthermore, M-CSF-induced CSF1R internalization (impaired by WNK1 absence or WNK kinase activity inhibition) was not rescued by PMA stimulation (Supplementary Fig. 18a, b). Finally, PMA, despite inducing equivalent levels of macropinocytosis to M-CSF-stimulated mouse and human progenitors, did not induce nuclear translocation of WNK1 (Supplementary Fig. 18c, d). Thus, our data demonstrate that M-CSF-stimulated macropinocytosis induces CSF1R internalization and concomitant translocation of WNK1 to the nucleus.

### M-CSF-stimulated macropinocytosis and WNK kinase activity regulate IRF8 protein levels

Given our finding that M-CSF-stimulated macropinocytosis induces WNK1 nuclear translocation and that WNK1 can regulate transcription factor activity and stability^49,52,53^, we hypothesized that WNK1/WNK kinase activity and M-CSF-stimulated macropinocytosis are controlling myeloid lineage fate through regulation of a macrophage lineage-enforcing transcription factor. One candidate is IRF8, a transcription factor that regulates macrophage versus neutrophil fate during hematopoiesis^58–60^. Indeed, we observed significant upregulation of IRF8 protein expression in progenitors stimulated with M-CSF (Fig. 5f) without increasing mRNA levels (Supplementary Fig. 19a). Strikingly, increased IRF8 protein expression observed in response to M-CSF was completely reversed upon inhibition of macropinocytosis or WNK kinase activity (Fig. 5f), again without affecting mRNA levels (Supplementary Fig. 19a). Conversely, overexpression of WNK1 significantly increased IRF8 expression in progenitors stimulated with M-CSF (Fig. 5h and Supplementary Fig. 19b), but not vehicle treated progenitors (Fig. 5g). Thus, our data suggest that M-CSF-stimulated macropinocytosis and subsequent WNK1 kinase activity and nuclear translocation is required for increased protein expression/stability of the macrophage lineage-enforcing transcription factor IRF8.

## Discussion

TRMs are essential for organismal development and tissue homeostasis. TRM development is dependent on CSF1R signaling, but the cell biological and molecular factors that differentially regulate myeloid progenitor fate remain an area of significant interest. Here, we make several unexpected, novel observations relevant to myeloid progenitor and precursor cells and molecular biology, myeloid cell lineage commitment, and TRM macrophage development. Across multiple lines of in vivo and in vitro evidence, we found that the serine-threonine kinase WNK1 is required for both mouse and human myeloid progenitor and monocyte differentiation into macrophages, and *Csf1r*^iCre^-mediated deletion of this kinase leads to perinatal lethality. Surprisingly, absence or perturbation of WNK1 not only disrupted macrophage development but also drove differentiation of human myeloid and mouse bone marrow progenitors towards the granulocyte lineage (e.g., neutrophils). We show that both mouse and human progenitors and monocytes perform continuous macropinocytosis in response to M-CSF in a WNK1/WNK kinase activity-dependent manner and that overexpression of WNK1 boosts continuous macropinocytosis. We also show that deletion of WNK1, inhibition of WNK kinase activity, or blocking M-CSF-stimulated macropinocytosis in mouse and human myeloid progenitors prevented macrophage differentiation and promoted neutrophil differentiation, whereas overexpression of WNK1 enhanced macrophage differentiation. Importantly, we found that WNK1 activity was directly required for macrophage differentiation downstream of M-CSF-stimulated macropinocytosis, as PMA-stimulated macropinocytosis was unable to rescue the myeloid differentiation defect. Finally, we found that M-CSF-stimulated macropinocytosis triggers CSF1R internalization and concomitant translocation of WNK1 to the nucleus, which in turn increases protein expression of IRF8. The M-CSF-induced increase in IRF8 protein expression depends on macropinocytosis and WNK1, as inhibition of macropinocytosis or WNK kinase activity suppresses IRF8 expression, while WNK1 overexpression boosts IRF8 protein levels. Taken together, we have discovered a new role for macropinocytosis and WNK1 in ensuring that myeloid progenitors develop into a specific lineage, which is essential for proper development.

Given the striking loss of TRMs across multiple tissues, we were initially surprised that monocyte numbers and frequencies were either unchanged or only modestly altered across tissues, which is consistent with previous observations in op/op mice and mice lacking CSF1R^17,61–63^. We did observe changes in the frequency of specific monocyte subsets in some tissues, including potential pre-monocytes, which may explain the observation that a small but significant fraction of mouse and human monocytes differentiate into neutrophils. These findings, together with a previous report suggesting that phenotypically-distinct monocytes can develop via one of two independent progenitors^18^, suggest that monocytes develop via a route independent of M-CSF/CSF1R^61,64–66^, but are subsequently unable to develop into TRMs in response to M-CSF. On the other hand, our sequencing analysis of MPP3s suggests that, at least in the bone marrow, the absence of WNK1 is expanding early granulocyte progenitors in response to M-CSF. This hypothesis is consistent with our observation that mouse and human myeloid progenitors also differentiate into neutrophils when WNK kinase activity is inhibited in vitro and that WNK1-deficient embryos have significantly more neutrophils systemically. Importantly, our finding of failed macrophage differentiation and enhanced neutrophil differentiation without altered myeloid cells (e.g., monocytes) in WNK1-deficient mice is also similar to previous work showing that IRF8 is important for macrophage versus neutrophil lineage commitment in primitive myelopoiesis^58,59^. Nevertheless, future studies using stage- and cell-type-specific Cre strains will be important to better delineate the stages at which WNK1 supports myelopoiesis.

Here, we link M-CSF-stimulated macropinocytosis and WNK1 activity/nuclear translocation with IRF8 protein expression. IRF8 is thought to control the lineage determination of different myeloid subsets through a complex circuit that involves the transcription factors ZEB2 and ID2^67–69^. Whether ZEB2, which was shown to control monocyte development^70^ and maintain tissue-specific identity in macrophages^67,71^, is involved in the identified CSF1R-macropinocytosis-WNK1-IRF8 pathway for macrophage differentiation is still unknown. Additionally, given the robustness of the phenotype of WNK1-deficient mice compared to IRF8-deficient mice, it is likely that other transcription factors, such as the myeloid-specific transcription factor PU.1, act in concert with WNK1 during macrophage differentiation.

Both M-CSF and IL-34 signal through CSF1R, yet IL-34 selectively supports microglia and Langerhans cells development and maintenance^64,72^. Despite increased neutrophilia in the brain, microglia numbers were relatively unchanged. Interestingly, IL-34 appears not to be required for embryonic development of microglia but instead for their maintenance. Indeed, IL-34-deficient mice exhibit reduced, not absent, adult microglia numbers^64,72^. This raises several intriguing questions. For instance, does IL-34 induce macropinocytosis in microglia progenitors, and if so, is it qualitatively different than that in myeloid progenitors? Interestingly, a recent report found that IL-34 is capable of inducing macropinocytosis in mature macrophages in vitro but is inhibited by the presence of certain amino acids^36^, suggesting that the nutrient environment may also inform induction and reliance on macropinocytosis in progenitors. Nevertheless, the cell biological processes that inform the unique developmental trajectory of microglia remain an intriguing area of interest.

Unlike other lysosomal degradative processes (e.g., efferocytosis, receptor-mediated endocytosis, autophagy), the biological importance of macropinocytosis in mammals remains less understood, possibly due to a lack of genetic and pharmacological tools developed to specifically perturb this endocytic process. Indeed, important caveats remain to our conclusions regarding the role of macropinocytosis for macrophage differentiation in vivo. For instance, our in vivo studies relied on adoptive transfer of high numbers of cells that do not perfectly recapitulate physiology. Additionally, EIPA, the only established inhibitor specific to macropinocytosis, is toxic at high doses or when used for prolonged periods. WNK463, however, was developed to therapeutically target WNK kinases in vivo and allows for prolonged use at higher doses^26^. To date, macropinocytosis has primarily been reported to be important for growth factor signaling in lower organisms (e.g., Dictyostelium), in terminally differentiated mammalian cells (e.g., macrophages), and in oncogenically transformed cells^15,35^. Here, we demonstrate for the first time that myeloid progenitors and monocytes perform macropinocytosis and establish that macropinocytosis is important for appropriate myeloid lineage commitment. Our data suggest a model whereby M-CSF/CSF1R signaling, either via macropinocytosis induction or prolonged cell surface signaling, determines the ultimate outcome of myeloid differentiation.

Finally, our proposed model raises an important question: if M-CSF can drive progenitor differentiation into either lineage, depending on whether macropinocytosis is induced or not, what are the distinct signals or factors that arise during macropinocytosis that drive one fate over another? The answer may relate to signaling in or at the macropinosome itself. For instance, cell fate may be influenced by the strength of amplification in growth factor signaling via macropinosome cup formation^57,73^. Our data demonstrating that M-CSF- but not PMA-stimulated macropinocytosis (1) induces CSF1R internalization, (2) WNK1 nuclear translocation, (3) increased IRF8 protein expression, and (4) macrophage differentiation, provides evidence to support a macropinosome-localized signaling hypothesis. Furthermore, our data suggests that WNK1 nuclear translocation is a key step in macrophage differentiation, possibly via its previously identified role in transcription regulation^49,52,53^. An alternative hypothesis is that solutes within the macropinosome itself could relay requisite signals to differentiate into one lineage over another, similar to a recent study demonstrating that mouse embryonic stem cells rely on macropinocytotic uptake of proteins to maintain stemness and switch to amino acid dependence following transition to committed states^74^. Solute-sensing signaling hubs, such as the mechanistic target of rapamycin (mTOR), have been previously shown to respond to growth factors within the macropinosome^75^. Consistent with this hypothesis, a previous report suggests that WNK1 can serve as a scaffold for the mTORC2 signaling complex^76^. Unlike our current study, however, WNK1 scaffold function was independent of its kinase activity, making this hypothesis unlikely. Nevertheless, determining the specific signal(s) arising in or at the macropinosome that dictate myeloid progenitor and monocyte lineage commitment is an exciting area of work moving forward.

## Methods

### Contact for reagent and resource sharing

Requests for resources and reagents should be directed to the Lead Contacts, Justin S. A. Perry (perryj@mskcc.org) or Alissa J. Trzeciak (trzeciaa@mskcc.org).

### Mice

Animals were housed at the Memorial Sloan Kettering Cancer Center (MSKCC) animal facility under specific pathogen free (SPF) conditions on a 12-h light/dark cycle under ambient conditions with free access to food and water. WNK1 deletion from myeloid cells was achieved by crossing *Csf1r*^*iCre*^ (Jackson Laboratories, Strain #021024) mice with *Wnk1*^*fl/fl*^ mice^77^ (a gift from Chou-Long Huang, University of Iowa), to obtain wildtype (WT; *Csf1r*^*Cre–*^*; Wnk1*^*fl/fl*^, Cre-), heterozygous (*Csf1r*^*Cre+*^; *Wnk1*^*fl/+*^, Cre±), and conditional WNK1 KO littermates (*Csf1r*^*Cre+*^; *Wnk1*^*fl/fl*^, Cre+). For *Csf1r*^*Cre+*^; *Wnk1*^*fl/fl*^ studies, animals were weighed and monitored to determine failure to thrive. For timed pregnancies, *Csf1r*^*Cre+*^; *Wnk1*^*fl/+*^ heterozygous males were single housed for 24 h before pairing with homozygous floxed (*Csf1r*^*Cre–*^*; Wnk1*^*fl/fl*^) females. Vaginal plugs were checked every 24 h on subsequent days after pairing to estimate time of conception. Pregnant dams were euthanized by CO_2_, and embryos were harvested at gestation days 9.5 and 10.5. For adoptive transfer experiments, CD45.1+ congenic 7–8-week-old male mice were obtained from Jackson Laboratories (Strain #0002014) and used as recipients for transfer and transplantation studies. All studies were approved by the Sloan Kettering Institute, Institutional Animal Care and Use Committee.

### Tissue collection and processing

For *Csf1r*^*Cre+*^; *Wnk1*^*fl/fl*^ immune cell characterization studies, mice between 3 and 4 weeks of age were euthanized and perfused using a 23 G needle with 20 mL of cold PBS without Ca^2+^ or Mg^2+^ (Corning, 21-040-CM) with 5 mM EDTA (Invitrogen, 15575-038). Blood was collected immediately following euthanasia, prior to perfusion via cardiac puncture, and transferred to heparin-coated tubes (Grenier, 450474). Brain, lungs, liver, kidneys, spleen, and heart were dissected, weighed, minced into small fragments, and incubated at 37 °C for 30 min in enzyme mix consisting of PBS with 160 Wünsch U/mL collagenase D (Sigma-Aldrich, 11088866001), 5% heat-inactivated FBS (Sigma, 1306C), and 10 mM HEPES (Gibco, 15630-080) (“PFH” buffer). The digestion reaction was stopped by incubation with 10 mM EDTA for 5 min. After enzyme digestion, all tissues were further dissociated by mechanical disruption using 70 µm cell strainers and an 18 G needle with 3-mL syringe plunger in 6-well plates containing 3 mL of cold PFH buffer. Single-cell suspensions were transferred to 15 mL tubes and pelleted by centrifugation at 1500 rpm for 5 min at 4 °C. Cell pellets were then resuspended in 38% Isotonic Percoll (10% 10X PBS with 90% Percoll (GE Healthcare, 17-0891-01)) in PFH buffer to gradient separate leukocytes by spinning at 2000 rpm for 30 min at RT with no brake. Bone marrow cells were collected by flushing femurs and tibias using a syringe and a 25 G needle with 10 mL PFH buffer. Bone marrow was dissociated by gently flushing through a 70 µm filter with the back of the syringe plunger. Collected blood, spleen (post-digestion), and bone marrow suspensions were spun down and lysed with 1 mL RBC lysis buffer (Sigma, R7757) for 5 min, washed with 10 mL PFH buffer, and centrifuged at 1500 rpm for 5 min at 4 °C.

For timed pregnancies, pregnant dams were euthanized at fetal time points embryonic (E)9.5, 10.5, and 11.5 fetuses were dissected from fetal horns. Fetuses were separated under a dissection microscope into the yolk sac, head, and trunk, and processed into single cell suspensions by placing in 100 µL of PFH buffer supplemented with 160 Wünsch U/mL collagenase D (Sigma-Aldrich, 11088866001) for 5 min at 37 °C, then gently pipetted up and down until all visible tissue was dispersed. Embryonic cells were then centrifuged at 1500 rpm for 5 min at 4 °C.

### Flow cytometry

Isolated single cell suspensions were washed once in PBS and stained with Live/Dead Fixable Aqua stain (Thermo Fisher Scientific, L34966) diluted 1:1000 in PBS (no FBS) for 30 min at RT, protected from light. Samples were then washed once in PFH buffer and resuspended in 50 µL of PFH containing purified anti-mouse CD16/32 antibody (1:50, BioXCell, BE0307) and incubated for 10 min on ice. Samples were then directly incubated with fluorochrome-conjugated antibodies (see Supplementary Table 1 below for antibodies and dilutions) for an additional 30 min on ice, then washed in PFH buffer and analyzed by flow cytometry using an Attune NxT flow cytometer (Invitrogen). In all tissues, live single cells were gated by exclusion of Live/Dead Aqua-positive dead cells, positive gating of side scatter and forward scatter, and doublet cell exclusion using forward scatter width against forward scatter area^78^. To calculate cell numbers per sample, counting beads (Thermo Fisher Scientific, C36950) were added at known concentrations to known volumes of samples and gated for back calculation after flow cytometry analysis. All post-acquisition analysis was conducted using FlowJo 10.10 (BD Biosciences).

### Microscopy

#### Tissue Processing

Brain, lungs, heart, kidney, liver, and spleen were harvested from mice following perfusion and immediately submerged in 4% formalin (Stat-Labs, 28530-1) for 24–48 h at 4 °C. Tissues were subsequently washed 3x in PBS without Ca^2+^ or Mg^2+^ (Corning, 21-040-CM), then submerged in 30% sucrose (Sigma, S0389) in PBS overnight or until they sunk. For small intestine, the entire small intestine was removed following perfusion, and fat was carefully dissected away from the mesenteric line, taking care not to disrupt the underlying tissues. The entire small intestine was then opened by making a continuous incision along the mesenteric line. Following this, the luminal contents were removed by gently agitation of the small intestine in two consecutive washes of ice-cold DPBS. The intestine was carefully flattened with the lumen facing upwards, and lightly fixed by drop application of cold 2% PFA. Following ~2 min of fixation, the entire small intestine was rolled onto a Dumont #4 blunt forceps. The intestinal roll was fixed in place by insertion of a 24-gauge needle through the entire roll, and the tissue was fixed overnight in 4% PFA at 4 °C prior to dehydration, embedding, and sectioning. For skin, following perfusion, hair was removed from the entire ear of the mouse using Nair hair removal cream applied for 5 min and then wiped off using a Kimwipe delicate task wiper. Following depilation, the entire ear was removed using sharp scissors and washed 3x in room temperature PBS to remove further hair and excess Nair cream. After washing, the entire ear was pinned flatly to a silicone-filled petri dish and fixed overnight in 4% PFA at 4 °C. After fixation, the ear tissue was washed 3x in cold PBS to remove excess PFA and the skin was then separated by grasping the free edge of the dorsal ear skin at the incised edge with a fine forceps and carefully peeling back the skin from the underlying connective tissue. All tissues were then embedded in Tissue-Tek OCT compound (Sakura, 4583), snap-frozen on dry ice, and immediately stored at −80 °C until sectioning. Ten-micrometer sections were cut on a Leica Microm HM550 and mounted on charged slides (Fisherbrand Superfrost Plus Gold, 15-188-48) for H&E and IF staining.

#### H&E staining

Briefly, tissue sections were brought to RT and dehydrated in 95% ethanol, stained with Harris Modified Hematoxylin (Fisher Scientific, SH26-500D) for 5 min, run under tap water until optimal bluing was achieved, then submerged into 0.5% acid alcohol for 10 s to stop the reaction. Sections were subsequently dehydrated in 95% ethanol and submerged in Eosin Y (Sigma-Aldrich, 1732-87-1) for 30 s. Sections were then treated with increasing concentrations of ethanol (70%, 95%, 100%), cleared with xylenes (Sigma-Aldrich, 534056), coverslip-mounted with Permount (Fisher-Scientific, SP15-100), and allowed to dry for 24 h prior to Differential Interference Contrast (DIC) imaging.

#### DIC Imaging

H&E and brightfield images were taken on an EVOS M5000 imaging system (Invitrogen) at 10×, 20×, and 40× magnification.

#### In situ Immunofluorescence

Tissue sections or fixed cultured cells (see isolation and culture of mouse and human progenitors) were brought to RT and rehydrated in PBS for 5 min, then blocked and permeabilized in 10% normal goat serum (Sigma-Aldrich, G9023) and 0.1% Triton-X-100 (Sigma-Aldrich, T9284) for 2 h. Individual sections were then separated using a barrier pen (Vector Labs, H-4000) and incubated in primary antibodies at 4 °C overnight in a humidified chamber (see Supplementary Tables 2 and 4 for mouse and human immunofluorescence antibodies, respectively). Slides were then washed 3x for 5 min in PBS. Sections were subsequently stained with Alexa Fluor Plus IgG H + L secondary antibodies at room temperature for 1 h (Life Technologies/Invitrogen) diluted 1:500. Finally, sections were washed 3x in PBS with the second to last wash containing nuclei dye Hoechst (Thermo Fisher, H3570) diluted 1:1000. Slides were mounted with coverslips and Prolong Gold Antifade (Thermo-Fisher P36934), sealed with clear nail polish, and allowed to dry overnight before imaging. Images (40X) were taken as 30–50 Z-stacks on an inverted Zeiss LSM 980 with Airyscan 2 and processed/analyzed in Fiji.

### Hybridization chain reaction (HCR) and image analysis

Embryonic (E) day 9.5 and 10.5 embryos were collected (see tissue collection and processing) and fixed overnight in 4% paraformaldehyde (PFA) in 1× PBS at 4 °C. After washes with PBS-T (1x PBS with 0.1% Tween-20), embryos were dehydrated and rehydrated in methanol and treated with 6% solution of hydrogen peroxide and treated with 10 μg/mL proteinase K and post-fixed with 4% PFA. The hybridization and amplification steps were performed as described in ref. ^79^ and probes (V3.0) against *Hhex*, *Kit*, and *Lyve1* were designed by Molecular Instruments, Inc. Stained embryos were soaked in DAPI for 48 h, mounted in 1% ultra-low gelling temperature agarose (Sigma), and cleared using Ce3D^80^. Stained embryos were imaged on a Nikon A1 confocal microscope, and image analysis was performed using the Imaris software (Bitplane, Inc.). The surface model tool was used to generate surfaces in the GFP channel to remove blood from all images to allow for more accurate quantification. To quantify *Kit* and *Lyve1* signal intensities within the *Hhex* region, a 3D *Hhex* surface was generated from each of the stained embryos, and spots with a diameter of 4 µm were used to cover the surface and project total sum intensities for each of the channels with a threshold cutoff at 3000.

### Isolation and culture of mouse bone marrow monocytes (precursor cells)

Monocytes from 3 to 4-week-old Cre− and Cre+ mice were obtained by flushing femurs and tibias with 10 mL DMEM (Fisher Scientific, MT10017CV) containing 20% FBS. To enrich for Ly6C+ monocytes, total bone marrow was subjected to negative selection via mouse monocyte isolation kit (MACS Miltenyi Biotec, 130-100-629), performed according to manufacturer instructions. Resultant monocytes were collected, counted, and cultured in αMEM (Corning, 15-012-CV) supplemented with 10% FBS and 1% PSQ, with 50 ng/mL recombinant murine M-CSF (PeproTech, 315-02) at 37 °C and 5% CO_2_ in a 12-well plate with vehicle or 10 µM WNK463 (Selleck Chemicals, S8358). Cells were monitored daily via fluorescent microscopy (EVOS 5500) and collected after 7 d of differentiation. Differentiated monocytes were then either harvested for immediate flow cytometry or exposed to indicated treatments prior to analysis.

### Isolation and culture of mouse bone marrow progenitors

Progenitors from 3 to 4-week-old Cre− and Cre+ mice were obtained by flushing femurs and tibias with 10 mL DMEM (Fisher Scientific, MT10017CV) containing 20% FBS. To enrich for lineage-negative cells, total bone marrow was incubated with a cocktail of biotinylated antibodies against a panel of lineage antigens (CD5, CD45R (B220), CD11b, Anti-Gr-1 (Ly6C/G), 7–4, and Ter-119 antibodies) and subsequently incubated with anti-biotin microbeads from the lineage cell depletion kit for mouse (MACs Miltenyi Biotec, 130-090-858), used as suggested by the manufacturer. Resultant progenitor lineage-negative cells were collected, counted, and cultured in DMEM containing 20% heat-inactivated FBS, 50 ng/mL murine stem cell factor (PeproTech, 250-03), 10 ng/mL recombinant murine IL-3 (PeproTech, 213-13), and 20 ng/mL recombinant human IL-6 (PeproTech, 200-06) without PSQ for 48–72 h at 37 °C and 5% CO_2_ (modified from ref. ^81^). For imaging studies, prior to progenitor isolation, 24 well TC- treated plates or 8 well glass chambers (Lab-Tek II, 155409) were coated for 1 h at RT with Poly-L-lysine hydrobromide (Sigma, P2636) at 0.5 mg/mL in water, washed 3x with water, and subsequently coated overnight at 4 °C with 2.5 mg/mL recombinant human Laminin (Thermo Fisher Scientific, A29249) in PBS. The following day, prior to stimulation with M-CSF, cells were serum-starved for 1 h. Images were captured for 1 h after M-CSF treatment unless otherwise stated.

### Isolation and culture of mouse neonatal myeloid progenitors

We established a novel protocol to generate neonatal myeloid progenitors^82^. Mouse epiblast stem cells (EpiSCs) (passage 16, C57BL/6J X 129S1/SvImJ F1 hybrid genetic background) were cultured on gelatin-coated dishes (MSKCC Media Preparation Core) with irradiated C57BL/6J mouse E13.5 fibroblasts in EpiSC media (50% DMEM-F12 (Gibco, 11320082), 50% Neurobasal medium (Gibco, 21103049) supplemented with 0.5% N2 supplement (Gibco, 17502048), 1% B27 supplement without vitamin A (Gibco, 12587010), 2 mM GlutaMAX (Gibco, 35050061), 1% penicillin-streptomycin (Gibco, 15140122), 0.1% 2-mercaptoethanol (Gibco, 21985023), 20 ng/mL activin A (Peprotech, 120-14 P), 12.5 ng/mL heat-stable bFGF (ThermoScientific, PHG0368), and 175 nM NVP-TNKS656 (Selleck Chemicals, S7238)). Media was changed daily, and cells were passaged every ~48 h at a 1:6 ratio after dissociation with Accutase (STEMCELL Technologies, 07922) into small clumps of 3–5 cells. All cells were maintained at 37 °C in 5% CO_2_ under normoxic conditions.

Upon confluency, EpiSCs were detached from the fibroblast layer using 0.1 Mandl U/µL collagenase IV (Gibco, 17104019), followed by dissociation using Accutase for 1–2 min to obtain a single-cell suspension. To differentiate EpiSCs into myeloid progenitors, EpiSCs were plated at a density of 60,000 cells/cm² onto tissue culture dishes coated with 16.7 µg/mL fibronectin (Sigma-Aldrich, FC010) and 10 µg/mL laminin (STEMCELL Technologies, 200-0117) in Lo FABC-R media, which consisted of CDM^83,84^ supplemented with 5 ng/mL FGF2 (ThermoScientific, PHG0368), 5 ng/mL Activin A (Peprotech, 120-14 P), 30 ng/mL BMP4 (R&D Systems, 314-BP), 3 µM CHIR 99021 (Tocris, 252917-06-9), 50 nM Chroman 1 (MedChem Express, HY-15392), and 5 µM Emricasan (Selleck Chemicals, S7775).

After 24 h, the medium was switched to Hi FAB media (CDM supplemented with 10 ng/mL FGF2, 10 ng/mL Activin A, and 40 ng/mL BMP4). On day 2, media was replaced with VF media (Essential 6 medium (Gibco, A1516401) containing 15 ng/mL VEGF (R&D, 293-VE) and 5 ng/mL FGF2). VF media was refreshed at the end of day 3. On day 4, cells were cultured in VFSI media (Essential 6 medium supplemented with 15 ng/mL VEGF, 5 ng/mL FGF2, 200 ng/mL SCF (PeproTech, 300-07), and 20 ng/mL IL-6 (R&D, 206-IL)). On day 5, cells were transferred to SITI media (Essential 6 medium containing 100 ng/mL SCF, 10 ng/mL IL-6, 30 ng/mL TPO (PeproTech, 300-18), and 30 ng/mL IL-3 (PeproTech, 200-03)), to complete their transition into myeloid progenitors. SITI media was changed daily until day 8. On Day 8, cells were dissociated into single cells with Accutase and resultant myeloid progenitors were collected, counted, and cultured in 75% IMDM with GlutaMAX (Gibco, 31980030), 25% F12 with GlutaMAX (Gibco, 31765035), 1:100 B27 supplement without vitamin A, 100 µg/mL penicillin-streptomycin supplemented with 10% FBS, with 100 ng/mL recombinant murine M-CSF (PeproTech, 315-02) at 37 °C and 5% CO_2_ in a 12-well plate with vehicle or 10 µM WNK463 (Selleck Chemicals, S8358) or 10 µM 5-[ethyl(1- methylethyl) amino]−2-pyrazinecarboxamide (EIPA, Tocris, 1154-25- 2) where indicated. Cells were monitored daily via fluorescent microscopy (EVOS 5500) and collected after 4 d of differentiation. Differentiated myeloid progenitors were then either harvested for immediate flow cytometry or exposed to indicated treatments prior to western blot analysis or microscopy.

### Mouse neutrophil isolation and stimulation

#### Isolation and nuclear imaging

Neutrophils from 3 to 4-week-old Cre− and Cre+ mice were obtained by flushing femurs and tibias with 10 mL DMEM (Fisher Scientific, MT10017CV) containing 20% FBS. To enrich for Ly6G+ neutrophils, total bone marrow was subjected to the mouse neutrophil isolation kit (Mojo Sort, Biolegend, 480057), performed according to manufacturer's instructions. For imaging studies, isolated Ly6G+ neutrophils were resuspended in 4% formalin (Stat-Labs, 28530-1) for 10 min at 4 °C, washed once in PBS, and resuspended in 250 ng/mL DAPI (Thermo Scientific, 62248) in PBS. Single cell suspensions were mounted on charged slides (Fisherbrand Superfrost Plus Gold, 15-188-48), mounted with coverslips, and immediately analyzed at 100× magnification on an inverted Zeiss LSM 980 to capture polymorphonuclear morphology.

#### Respiratory burst assay

Isolated neutrophils from Cre− and Cre+ mice were immediately subjected to respiratory burst assay kit as per manufacturer’s instructions (Abcam, ab236210). Briefly, 5 × 105 neutrophils were cultured in polypropylene tubes, stimulated with 5 ng/mL of Phorbol 12-myristate 13-acetate (PMA) for 1 h at 37 °C, stained with DHR-123, and analyzed on an Attune NxT flow cytometer (Invitrogen).

### Adoptive transfer experiments

Bone marrow monocytes or progenitors were isolated from 8-to-12-week-old CD45.1 congenic male and female donors. To enrich for Ly6C+ monocytes, total bone marrow was subjected to negative selection via mouse monocyte isolation kit (MACS Miltenyi Biotec, 130-100-629), performed according to manufacturer instructions. For progenitors, total bone marrow was incubated with a cocktail of biotinylated antibodies against a panel of lineage antigens (CD5, CD45R (B220), CD11b, Anti-Gr-1 (Ly6C/G), 7–4, and Ter-119 antibodies) and subsequently incubated with anti-biotin microbeads from the lineage cell depletion kit for mouse (MACs Miltenyi Biotec, 130-090-858), used as suggested by the manufacturer. Total Ly6C+ or Lin− cells were counted using Cell Counter chamber slides (Invitrogen, C10283) in a Countess II FL (Invitrogen). Approximately 1 × 10^6^ CD45.1+ Ly6C+ or 1 × 10^5^ Lin- cells were diluted in sterile PBS prior to intraperitoneal injection^32,85–89^. Injections occurred at P5, P8, and P11 or P2, P5, P8 as indicated. Mice were either monitored for extended survival or euthanized at P28 (2–3 weeks after final injection). Denoted organs/tissues were analyzed for the fraction of CD45.1+ cells (chimerism) in the total CD45+ pool. Alternatively, CD45.1 congenic mice were used as wild-type recipients for bone marrow transplantation experiments, where cage mates were treated with 1 dose of 900 rad lethal irradiation and put on broad-spectrum antibiotics diluted in drinking water at 2 mg/mL for 1 week following irradiation (Baytril 100, Enrofloxacin, Bayer). Irradiated animals were IP injected with 5 × 10^5^ Lin- cells obtained from Cre- or Cre+ mice as described above within 24 h of lethal irradiation.

### Single cell RNA sequencing

To perform single cell RNA sequencing (scRNA-seq) of Cre- and Cre+ myeloid progenitors, MPP3s (Lin- cKit+ Sca-1+ Flt3- CD48+ CD150-) were sorted using multi-color flow cytometry (FACS Aria, BD) into fresh tubes containing PBS with 0.04% BSA (Sigma-Aldrich, A8806). Cells were washed 1x and resuspended in PBS containing 0.04% BSA at a final concentration of 700–1300 cells/µL. Cell viability was confirmed to be above 80% via staining with 0.2% (w/v) Trypan Blue (Countess II). Each mouse (*n* = 3 Cre−, *n* = 4 Cre+) was labeled separately by staining with MHC Class I-barcoded antibodies bearing hashtags 1–7 (BioLegend, A0301-A0307). Library prep and sequencing were performed by the Single Cell Research Initiative (SCRI) at MSKCC as follows: scRNA-seq was performed on a Chromium instrument (10× Genomics) following the user guide manual for 3′ v3.1. Cells were captured in droplets, then subjected to reverse transcription and cell barcoding. Following this step, emulsions were broken, and cDNA was purified using Dynabeads MyOne SILANE, followed by PCR amplification per manual instructions. Approximately 30,000 cells were targeted for each sample. Samples were multiplexed together on one lane of a 10X Chromium following a published cell hashing protocol^90^. Final libraries were sequenced on the Illumina NovaSeq S4 platform (R1–28 cycles, i7–8 cycles, R2–90 cycles). The cell-gene count matrix was constructed using the Sequence Quality Control (SEQC) package^88^. Viable cells were identified based on library size and complexity, with “cells” exhibiting >20% of transcripts derived from mitochondria excluded from analysis. The resulting matrix data were further processed and analyzed using R version 4.2.2 (R Core Team 2022) and the Seurat package version 4.3.0^91^. Seurat objects were created using only genes appearing in at least three cells. Cells were further filtered to exclude those with <200 genes detected or >7000 genes detected. Read counts were then normalized within each sample using the SCTransform function with the glmGamPoi method, percent mitochondrial reads as the vars.to.regress argument, and vst.flavor v2. The RunPCA function was then run with npcs set to 30. The graph representing cells with similar expression patterns was generated with the FindNeighbors function using the 30 largest principal components. Cell clusters were generated using the Louvain algorithm implemented by the FindClusters function with a resolution parameter equal to 0.6. Marker genes for each cluster were determined using the Wilcoxon test on the SCT normalized counts after running the PrepSCTFindMarkers function using the function FindAllMarkers and including only genes with log fold changes >0.25 and Bonferroni-corrected *P* values < 0.01. Dimensionality reduction by Uniform Manifold Approximation and Projection was performed using the RunUMAP function with the 30 largest principal components. All visualizations of scRNA-seq data were generated using the Seurat^91^, scCustomize, and ggplot2 packages.

### Isolation and culture of human blood monocytes (precursor cells)

The use of human material was approved by the MSKCC Institutional Biosafety Committee. Peripheral blood mononuclear cells (PBMCs) isolated from deidentified healthy blood donors, who provided written informed consent, were obtained by gradient separation using 1-Step Polymorphs (Accurate Chemical, AN221725). CD14+ monocytes were isolated with Mojosort human CD14 nanobeads (BioLegend, 480093). 5 × 10^6^ monocytes were seeded and differentiated into macrophages for 7 d in RPMI-1640 (Corning, 17-105-CV) supplemented with 10% FBS and 1% PSQ, with 50 ng/mL recombinant human M-CSF (PeproTech, 300-25) at 37 °C and 5% CO_2_. Human monocyte-derived macrophages (HMDMs) were re-seeded at 2 × 10^5^ cells/mL (1 mL per well) in a 12-well plate with vehicle or 10 µM WNK463 (Selleck Chemicals, S8358). Cells were monitored daily via fluorescent microscopy (EVOS 5500) with images presented in the manuscript collected after 7 d of differentiation. HMDMs were then harvested by scraping cells directly into media, centrifuged at 2500 rpm in a benchtop centrifuge for 5 min at RT, incubated with FC block and indicated antibodies, then analyzed by flow cytometry (see Supplementary Table 3 for human antibodies).

### Human pluripotent stem cell (hPSC) cultures

Human PBMCs obtained from deidentified donors who provided written informed consent were reprogrammed into pluripotent stem cells (PSCs) using recombinant Sendai viral vectors that express Yamanaka factors^92^ by the MSKCC Stem Cell Core. PSCs were maintained on CF1 mouse embryonic fibroblasts (ThermoFisher, A34181) in ESC medium (ThermoFisher, 10829018) supplemented with bFGF (PeproTech, 100-18B). EBs were formed by disassociating and maintaining PSCs in ESC media supplemented with a Rho-associated protein kinase (ROCK) inhibitor (10 μM; Sigma, Y0503) while orbitally shaking (100 rpm) for 6d^93^. Mature EBs were transferred to tissue culture-treated 6-well plates and maintained in APEL media (Stem Cell Technologies, 5270) supplemented with 5% PFHM-II (Fisher, 12040077), 1% penicillin-streptomycin, human IL-3 (25 ng/mL; PeproTech, 200-03) and M-CSF (50 ng/mL; PeproTech, 300-25), in the presence of either vehicle or 10 µM WNK463 (Selleck Chemicals, S8358) for an additional 7 d. In some experiments, myeloid progenitors were derived normally, then treated with M-CSF in the presence of vehicle or WNK463 (10 µM) for an additional 5–10 d to produce terminally-differentiated macrophages. Resultant PSC-derived macrophages were then analyzed by fluorescent microscopy or flow cytometry, as in analyzes performed with HMDMs.

### WNK1-mRuby transfection

The pcDNA3.1_mRuby2-rWNK1 plasmid^44^ was a generous gift from Dr. Arohan R. Subramanya, University of Pittsburgh School of Medicine. Mouse bone marrow progenitor cells were isolated (see isolation and culture of mouse bone marrow progenitor cells), and plasmids were transfected using Lipofectamine 3000 (ThermoFisher, Lipofectamine 300 Reagent, 100022050, P3000 Reagent, 100022057) in OptiMEM (Gibco, 11058-021) per manufacturer's instructions. When transfected cells were used for imaging, they were replated 48 h later on 8-well glass chambers (Lab-Tek II, 155409) coated with Poly-L-lysine hydrobromide and recombinant human Laminin (see isolation and culture of mouse bone marrow progenitors). Images were captured for 1 h after M-CSF treatment, then harvested for flow cytometry or western blots unless otherwise stated.

### Macropinocytosis assays

To assess macropinocytosis, the reagents DQ-Red BSA (Thermo Fisher Scientific, D12051), Tetramethylrhodamine (TMR)-dextran, 70,000 MW, lysine fixable (Thermo Fisher Scientific, D1818) or Fluorescein (FITC)-dextan, 70,000 MW, lysine fixable (Thermo Fisher Scientitic, D1822) was added to indicated cells at a concentration of 10 μg/mL of DQ-Red BSA or 1 mg/mL dextran with 100 ng/mL of M-CSF for 1 h or unless otherwise indicated. In some cases, the plasma membrane was stained using CellMask Far Red Cells were then either live imaged fixed for 10 m in 4% PFA and stained with intracellular antibodies (see Supplementary Tables 2 and 4 for mouse and human immunofluorescence antibodies, respectively) before being analyzed via fluorescent microscopy (EVOS M5000 or Zeiss LSM 980) or washed 1x with PBS and analyzed by flow cytometry. In some experiments, indicated cells were collected and lysed for western blot. For growth factor-independent macropinocytosis induction, Phorbol 12-myristate 13-acetate (PMA) was added instead of M-CSF at a concentration of 5 ng/mL. To inhibit macropinocytosis, the compounds 5-[ethyl(1-methylethyl) amino]−2-pyrazinecarboxamide (EIPA, at 1 µM, 10 µM, or 50 µM where indicated, Tocris, 1154-25- 2), LY294002 (50 μM, SelleckChem, S1105), or Apilimod (10 μM, MedChemExpress, HY-14644) were added 1 h prior to macropinocytosis induction.

To quantify macropinocytosis via microscopy, Z-stacked images were 3D-projected in Fiji software, channels were split, and Just Another Colocalization Plugin (JACoP, v2.1.4^94^) was used to threshold each fluorochrome as follows: CSF1R, 250; WNK1, 20; and Nuclei, 200. Manders’ Coefficients (MCC) were calculated based on amount of fluorochrome overlap of (i.e., fraction of A overlapping B), and percentage of colocalization was calculated by multiplying MCC numbers by 100.

### Western Blotting

After treatment with cytokines and/or inhibitors, cells were washed 1x in HBSS without Ca2+ or Mg2+ (Gibco, 14170-112) as phosphate-containing buffers can disrupt phosphorylation assay data. Pelleted cells were lysed in Pierce RIPA buffer (Thermo Scientific, 89901) containing protease and phosphatase inhibitor cocktails (Roche, 04 906 845 001). Lysates were then used in total-protein western blots (Nu-Page 3–8% Tris-Acetate Gel, Invitrogen, EA0375BOX) or phosphorylation blots (6 or 7.5% Phos-Tag gels, Wako/Fujifilm, 192-18001). Protein blots were transferred to PVDF membranes (BioRad, 1620177) using the Trans-Blot Turbo System (BioRad, 170-4270) according to the manufacturer's protocol. Membranes were blocked for 30 min with 5% Milk in TBS-T (TBS: Teknova, T1680, Tween-20: Sigma, P1379), washed 3x, then probed overnight at 4 °C with primary antibodies (see Supplementary Tables 5 and 6 for mouse and human western blot antibodies, respectively). Antibodies were diluted in TBS-T containing 3% BSA. Secondary goat, rabbit, and mouse antibodies directly linked to HRP (Amersham) were diluted at 1:5000 in 3% BSA in TBS-T and added to membranes for 1 h at RT. Membranes were washed 3x and treated with SuperSignal West Pico Plus ECL (Thermo Scientific, 34578) for 5 min in the dark. Membranes were imaged using the iBright 1500 imaging system (Invitrogen) to analyze chemiluminescence. Densitometry was calculated using Fiji, and expression was normalized by dividing by the total protein of the loading control, and expression was represented by calculating the fold change of each condition to vehicle control bands.

### Reverse transcription quantitative PCR

Total RNA was extracted from progenitor cells after 1h M-CSF stimulation (see isolation and culture of mouse bone marrow progenitor cells) using the NucleoSpin RNA kit (Macherey-Nagel, 740955.250). The QuantiTect Reverse Transcription Kit (Qiagen, 205314) was used to synthesize cDNA according to the manufacturer’s instructions. Quantitative gene expression for mouse genes was performed using TaqMan probes and TaqMan Fast Advanced Master Mix (Applied Biosystems/ThermoFisher, 4444554) with a QuantStudio 6 Pro (Applied Biosystems). The murine *Irf8* gene expression assay *(*ThermoFisher, Mm00492567_m1 FAM) was tested and quantified relative to *Gapdh (*ThermoFisher, Mm99999915_g1 FAM).

### Statistics and reproducibility

Statistical analyzes were performed using GraphPad Prism 10.2.3. Significance was determined using Kaplan–Meier’s Log-Rank Mantel-Cox test, independent samples Student’s *t* tests, or one-way ANOVA, according to data requirements. All experiments were performed independently at least three times unless otherwise stated.

### Reporting summary

Further information on research design is available in the Nature Portfolio Reporting Summary linked to this article.

## Supplementary information

Supplementary Information

Description of Additional Supplementary Files

Supplementary Data 1

Supplementary Data 2

Reporting Summary

Transparent Peer Review file

## Source data

Source Data

## Data Availability

Single-cell RNA sequencing data for this experiment have been deposited at the Gene Expression Omnibus under accession number GSE196336. All data are included in the Supplementary Information or available from the authors, as are unique reagents used in this Article. The data used to generate charts and graphs is available in the Source Data file whenever possible. All other data are available from the corresponding author upon reasonable request. Source data are provided with this paper.
