# Supplementary material for: WNK1 mediates M-CSF-induced macropinocytosis to enforce macrophage lineage fidelity
Source: Nat Commun. 2025 May 28;16:4945. doi: 10.1038/s41467-025-59901-0 (PMC12120055; doi:10.1038/s41467-025-59901-0)
Supplement: Supplementary file 1 — Supplementary Information [file 41467_2025_59901_MOESM1_ESM.pdf]

WNK1 mediates M-CSF-induced macropinocytosis to enforce macrophage lineage fidelity

Alissa J. Trzeciak^ … Justin S. A. Perry^.

^Corresponding authors: perryj@mskcc.org and trzeciaa@mskcc.org.

Supplementary Figures 1 – 19

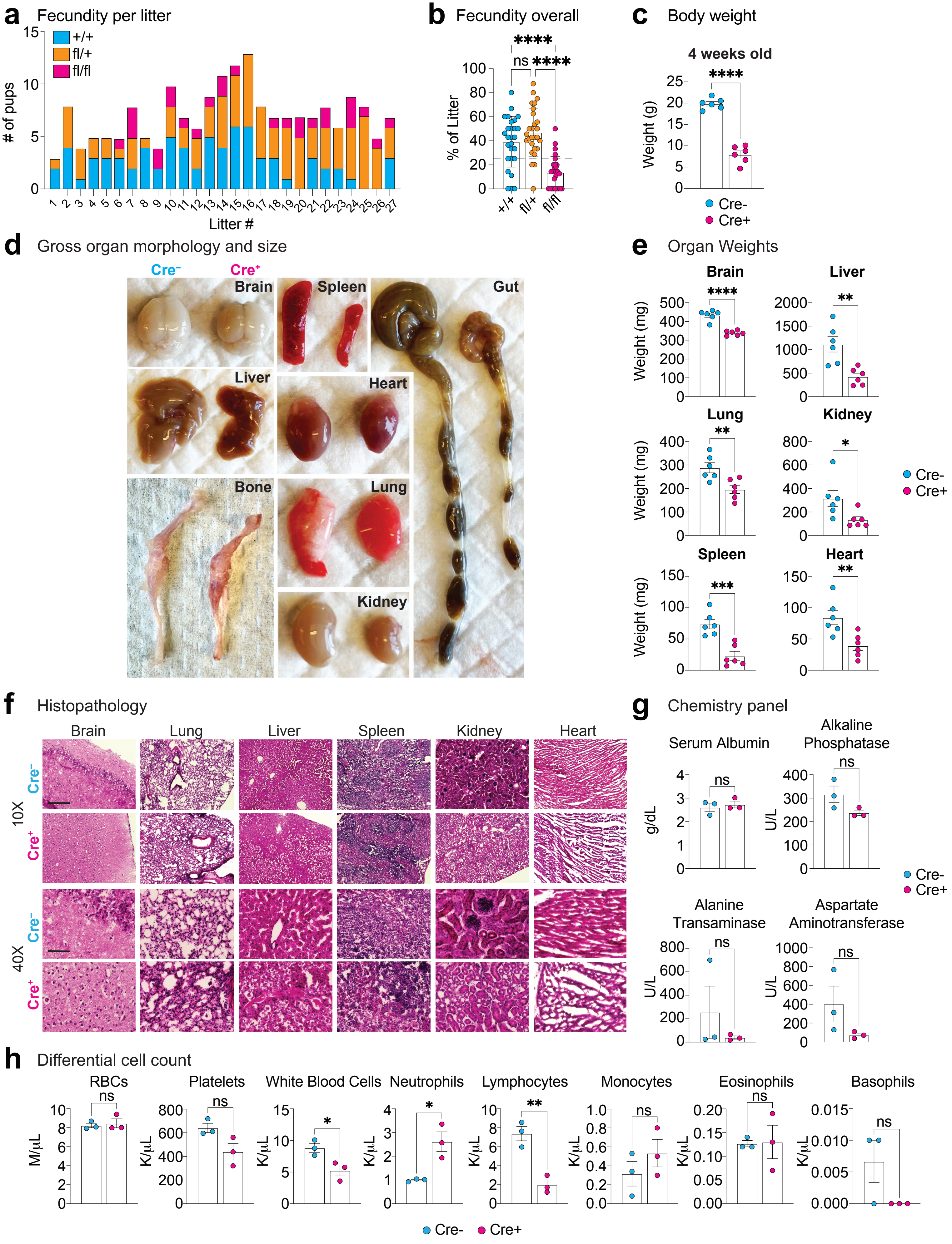

a Fecundity per litter
+/+
fl/+
fl/fl
# of pups
Litter #
b Fecundity overall
% of Litter
ns
****
+/+
fl/+
fl/fl
c Body weight
4 weeks old
Weight (g)
Cre-
Cre+
d Gross organ morphology and size
Cre⁻
Cre⁺
Brain
Spleen
Gut
Liver
Heart
Bone
Lung
Kidney
e Organ Weights
Brain
Liver
Lung
Kidney
Spleen
Heart
Weight (mg)
Cre-
Cre+
f Histopathology
Brain
Lung
Liver
Spleen
Kidney
Heart
10X
40X
Cre⁻
Cre⁺
g Chemistry panel
Serum Albumin
Alkaline Phosphatase
Alanine Transaminase
Aspartate Aminotransferase
g/dL
U/L
Cre-
Cre+
h Differential cell count
RBCs
Platelets
White Blood Cells
Neutrophils
Lymphocytes
Monocytes
Eosinophils
Basophils
M/μL
K/μL
Cre-
Cre+

Supplementary Fig. 1. Tissue-resident macrophages are absent in many organs of *Csf1r^{Cre+}; Wnk1$^{fl/fl}$* mice.

(a, b) *Csf1r^{Cre+}; Wnk1$^{fl/fl}$* mice do not follow Mendelian inheritance. **(a)** Prevalence of homozygous wildtype (blue), heterozygous (fl/+, orange), and homozygous WNK1-deficient mice (fl/fl, magenta) per litter, and **(b)** percentage of genotype in each litter. n= 27 litters. Data shown as mean ± SEM. Statistical significance was determined via one-way ANOVA. ****p < .0001, ns = not significant.

(c-e) *Csf1r^{Cre+}; Wnk1$^{fl/fl}$* mice are severely runted. Weights (**c**) of *Csf1r^{Cre+}; Wnk1$^{fl/fl}$* (Cre+, magenta, n=6) vs. *Csf1^{Cre-}; Wnk1$^{fl/fl}$* (Cre-, blue, n=6) mice before humane sacrifice at 4 weeks of age. Data are representative of three independent experiments. (**d**) Representative images of organs from Cre- and Cre+ mice. Shown are (clockwise from top left) brain, spleen, colon, kidneys, bones, liver, heart, and lungs. Images are representative of organ analysis from six Cre- mice and six Cre+ mice. (**e**) Shown are major organs (clockwise from top left: brain, liver, kidney, heart, spleen, and lung) weights (mg) of Cre+ (magenta, n=6) vs. Cre- (blue, n=6). Data are representative of three independent experiments. Data shown as mean ± SEM. Statistical significance was determined via independent samples *t*-test. *p < .05, **p < .01, ***p < .001, ****p < .0001.

(f) Hematoxylin & eosin (H&E) staining of brain, lung, liver, spleen, kidney, and heart. 10X scale bar, 300µm, 40X scale bar, 50 µm. Data are representative of four independent experiments.

(g) Serum chemistry parameters of *Csf1r^{Cre+}; Wnk1$^{fl/fl}$* (Cre+, magenta, n=3) vs. *Csf1^{Cre-};Wnk1$^{fl/fl}$* (Cre-, blue, n=3) mice before sacrifice at 4 weeks of age including (clockwise from top left) albumin (g/dL, grams per deciliter), alkaline phosphatase, aspartate aminotransferase, and alanine transaminase (U/L, units per liter) did not constitute trends or correlate with clinical abnormalities. Data shown as mean ± SEM. ns = not significant.

(h) *Csf1r^{Cre+}; Wnk1$^{fl/fl}$* (Cre+, magenta, n=3) vs. *Csf1^{Cre-}; Wnk1$^{fl/fl}$* (Cre-, blue, n=3) mice had decreased total white blood cell counts, decreased lymphocyte numbers, and increased neutrophils before sacrifice at 4 weeks of age. Red blood cell (RBCs, M/µl, millions per cubic microliter), platelet, monocyte, eosinophil, and basophil numbers were not significantly different (K/µl, thousands per cubic microliter. Data shown as mean ± SEM. *p < .05, **p < .01, ns = not significant.

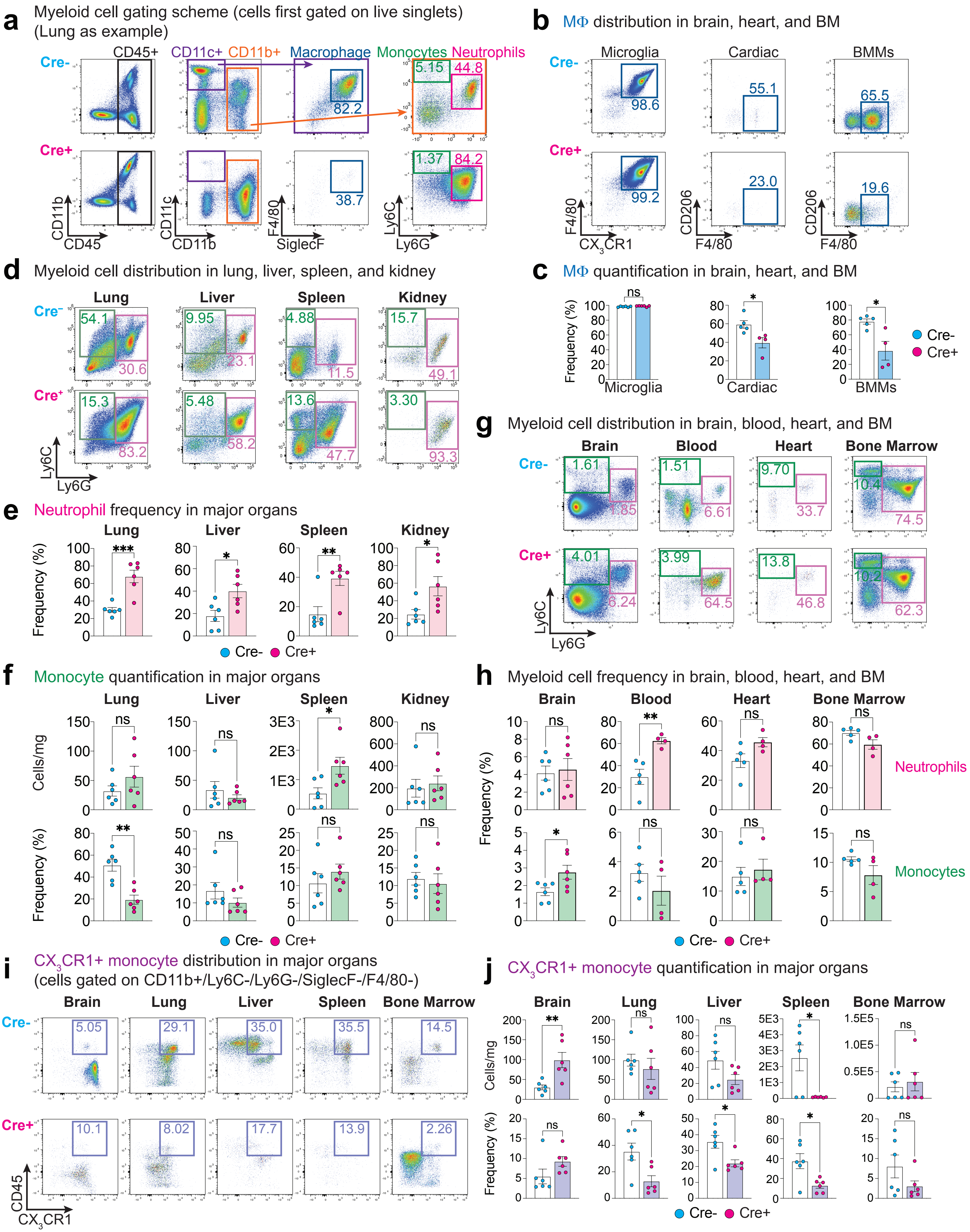

a
Myeloid cell gating scheme (cells first gated on live singlets)
(Lung as example)
b
MΦ distribution in brain, heart, and BM
c
MΦ quantification in brain, heart, and BM
d
Myeloid cell distribution in lung, liver, spleen, and kidney
e
Neutrophil frequency in major organs
f
Monocyte quantification in major organs
g
Myeloid cell distribution in brain, blood, heart, and BM
h
Myeloid cell frequency in brain, blood, heart, and BM
i
CX3CR1+ monocyte distribution in major organs
(cells gated on CD11b+/Ly6C-/Ly6G-/SiglecF-/F4/80-)
j
CX3CR1+ monocyte quantification in major organs
Cre-
Cre+

Supplementary Fig. 2. Analysis of tissue-resident macrophages, neutrophils, and monocytes in *Csf1r*$^{Cre+}$*; Wnk1*$^{fl/fl}$ mice.

(a) Example of flow cytometry gating scheme for **Fig. 1c, d** and Supplementary Fig. 2b-h. Shown is gating of cells in the lung.

(b-h) Data are from experiments performed in **Fig. 1c, d**. Flow cytometry analyses of tissue-resident macrophages, neutrophils (Ly6G+), and monocytes (Ly6C+) in essential tissues obtained, analyzed, and presented as in **Fig. 1c, d**. Data are from at least four *Csf1r*$^{Cre+}$; *Wnk1*$^{fl/fl}$ mice and five littermate controls. Data shown as mean ± SEM. Statistical significance was determined via independent samples *t*-test. *$p < .05$, **$p < .01$, ***$p < .001$, ns = not significant.

(i, j) Flow cytometry analysis of CX_3CR1+ monocytes isolated from experiments performed in **Fig. 1c, d**. Shown are representative flow cytometry plots (**i**) and summary plots of absolute numbers per milligram of tissue and frequencies (**j**) of CX_3CR1+ monocytes. Data are from six *Csf1r*$^{Cre+}$; *Wnk1*$^{fl/fl}$ mice and six littermate controls. Data shown as mean ± SEM. Statistical significance was determined via independent samples *t*-test. *$p < .05$, **$p < .01$, ns = not significant.

a Immunofluorescence (Lung, Liver, Spleen, and Kidney)

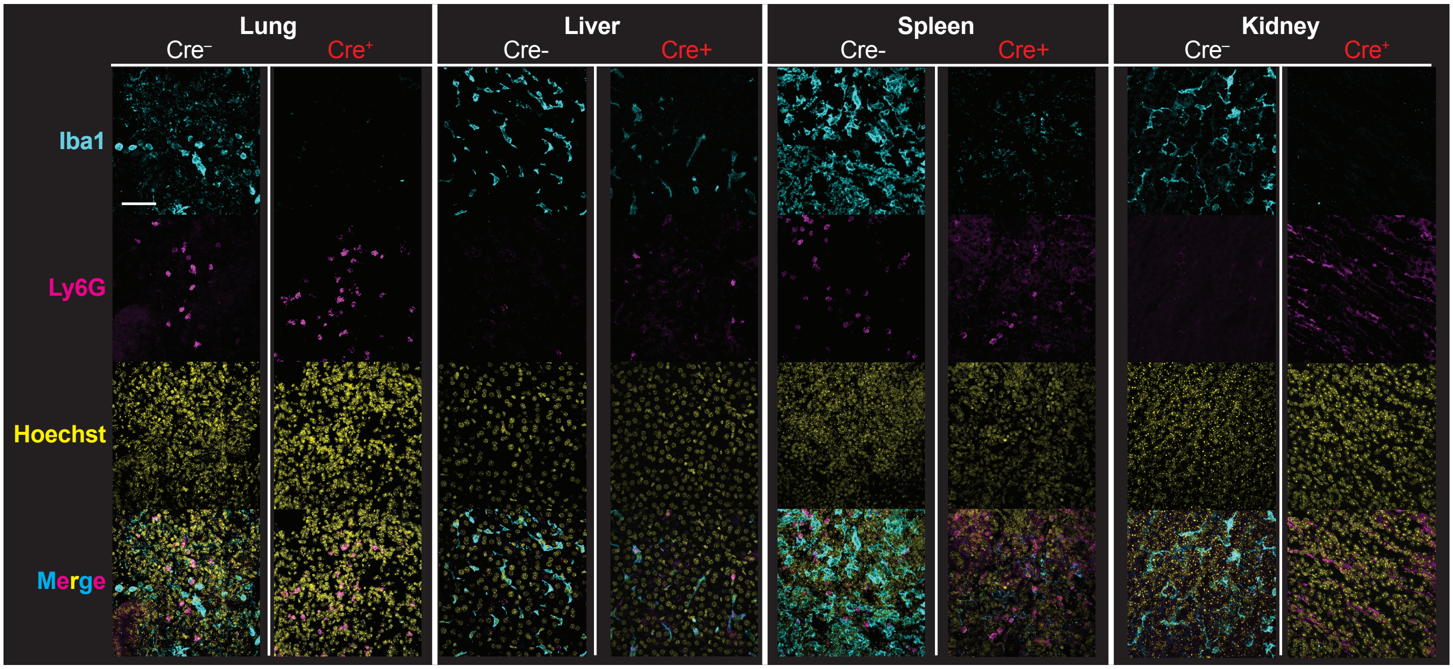

b Immunofluorescence (Small Intestine, Brain, and Heart)

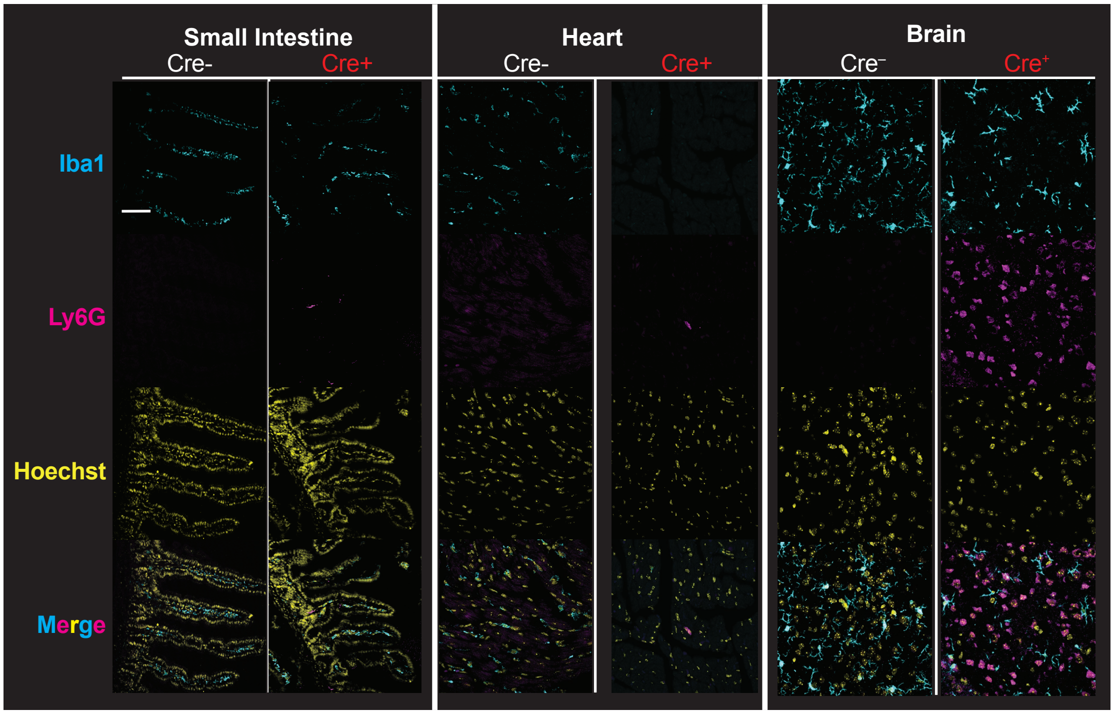

c Immunofluorescence (Skin)

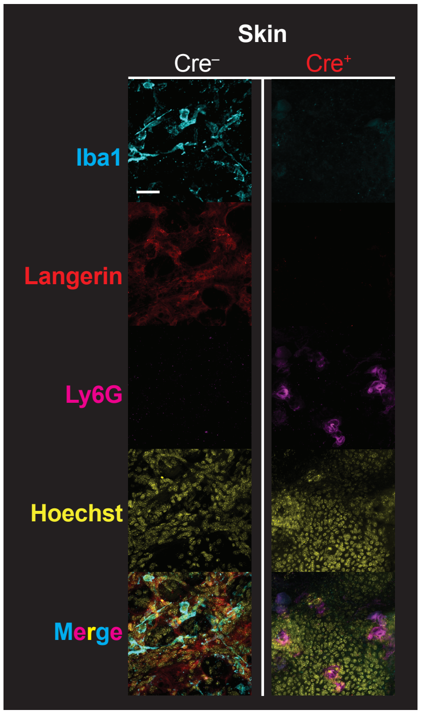

Supplementary Fig. 3. Immunofluorescence analysis of tissue-resident macrophages and neutrophils in *Csf1r*$^{Cre+}$*; Wnk1*$^{fl/fl}$ mice.

(a-c) Immunofluorescence analysis of macrophages and neutrophils in the lung, liver, spleen, and kidney (**a**; corresponding to **Fig. 1c, d**), in the small intestine, heart, and brain (**b**; corresponding to Supplementary Fig. 2b, c), and skin (**c**). Confocal microscopy analysis was performed using Iba1 for macrophages (cyan) and Ly6G for neutrophils (magenta). The skin was stained with an addition marker (Langerin, red) to denote Langerhans cells. Nuclei were labeled using Hoechst (yellow). Scale bar, 20μm. Images are representative of four independent experiments.

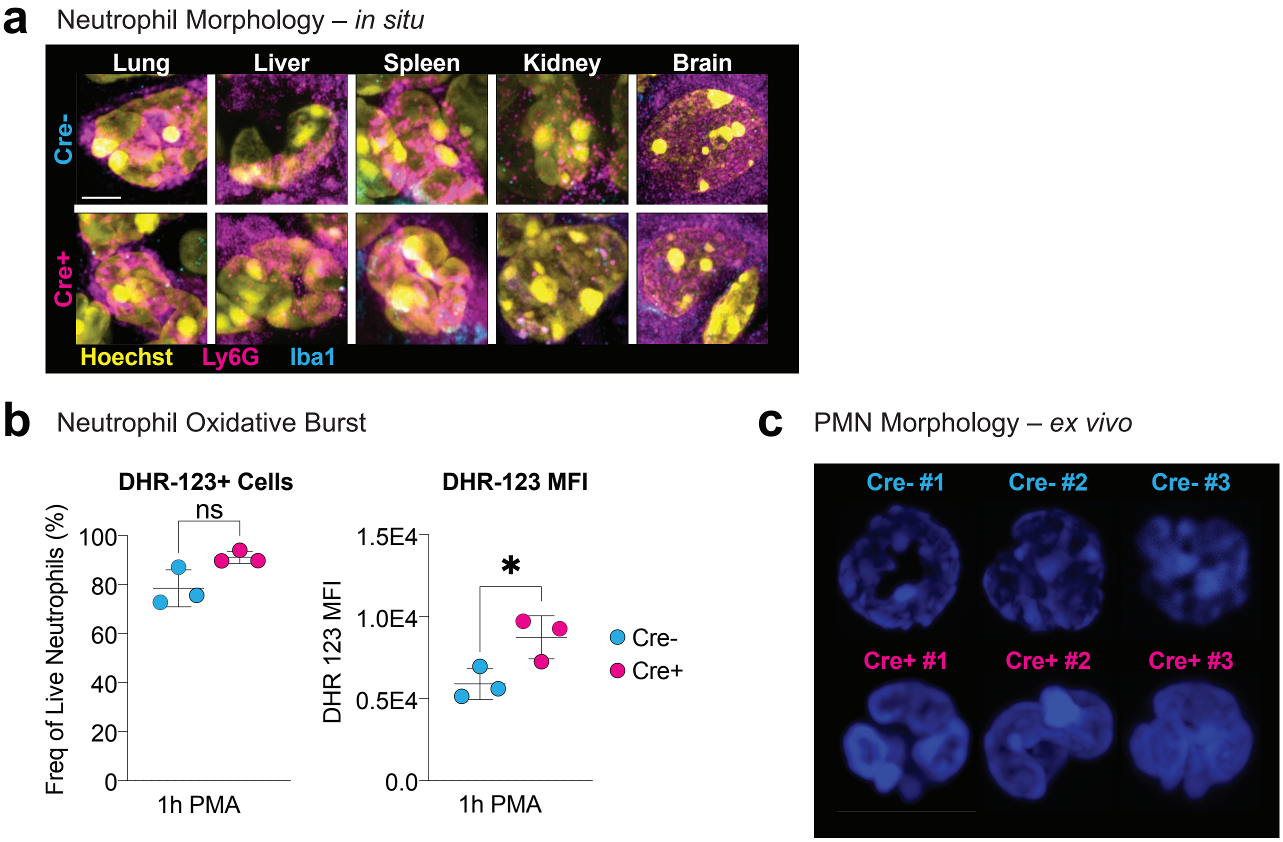
a
Neutrophil Morphology – in situ
Lung
Liver
Spleen
Kidney
Brain
Cre-
Cre+
Hoechst
Ly6G
Iba1
b
Neutrophil Oxidative Burst
DHR-123+ Cells
ns
Freq of Live Neutrophils (%)
100
80
60
40
20
0
1h PMA
DHR-123 MFI
*
DHR 123 MFI
1.5E4
1.0E4
0.5E4
0.0
1h PMA
Cre-
Cre+
c
PMN Morphology – ex vivo
Cre- #1
Cre- #2
Cre- #3
Cre+ #1
Cre+ #2
Cre+ #3

Supplementary Fig. 4. Function and morphology of neutrophils from *Csf1r*$^{Cre+}$*; Wnk1*$^{fl/fl}$ mice.

(a) High magnification confocal microscopy analysis was performed of lung, liver, spleen, kidney, and brain neutrophils (Ly6G, magenta) as in **Fig. 1d** and Supplementary Fig. 3. Nuclei were labeled using Hoechst (yellow). Scale bar, 5µm. Images are representative of four independent experiments.

(b) Flow cytometric analysis of respiratory burst assay performed on neutrophils purified *ex vivo* from Cre- (n=3, blue dots) and Cre+ (n=3, magenta dots). Frequencies of Dihydrorhodamine-123 (DHR-123) cells (*left*) and MFI (*right*) were analyzed after 1h of phorbol 12-myristate 13-acetate (PMA) stimulation. Data shown as mean ± SEM. Statistical significance was determined via independent samples *t*-test. *$p < .05$, ns = not significant.

(c) Immunofluorescence analysis of *ex vivo* isolated neutrophil polymorphonuclear (PMN) morphology. Nuclei were labeled with DAPI from n=3 mice per group.

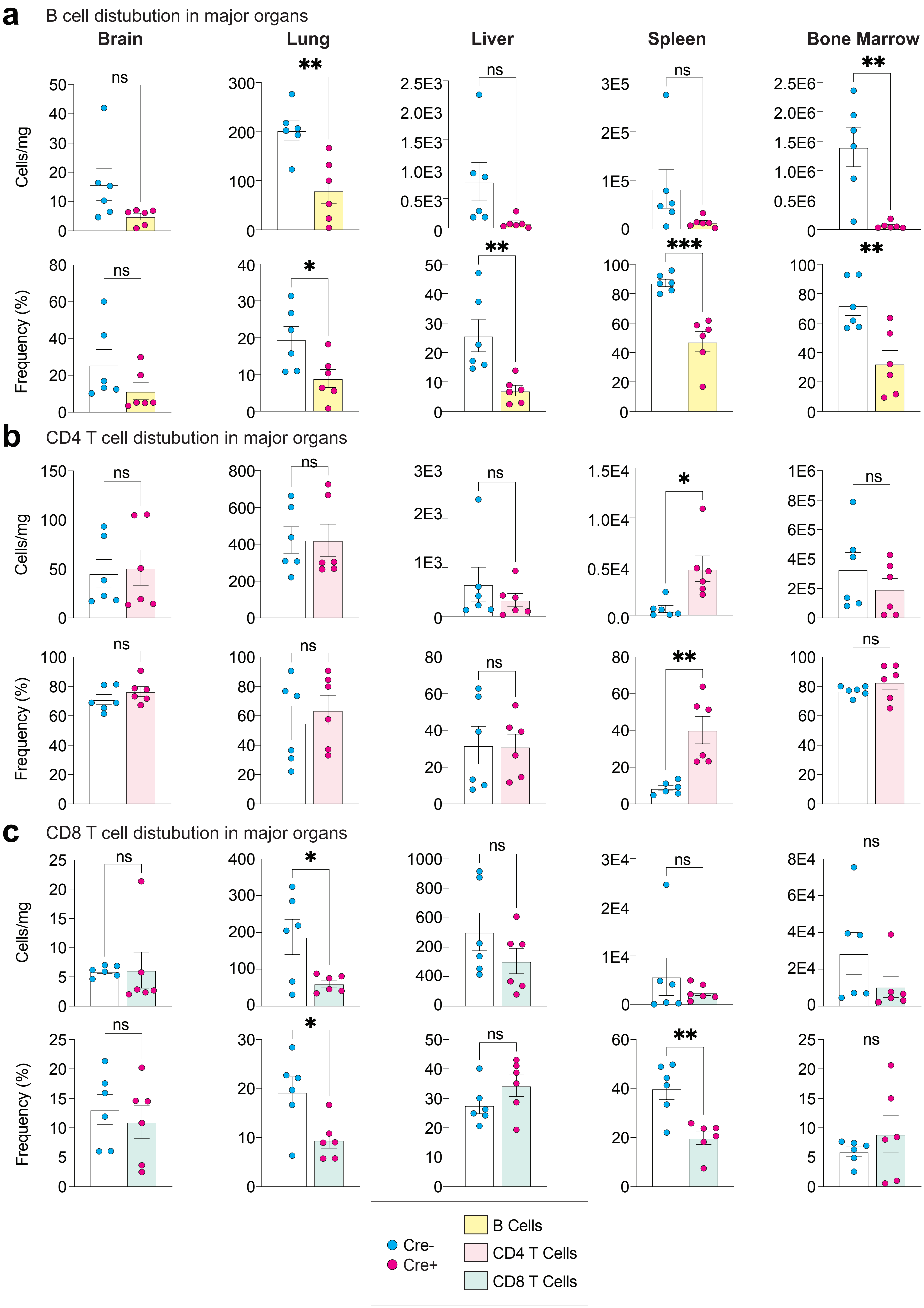
a
B cell distubution in major organs
Brain
Lung
Liver
Spleen
Bone Marrow
Cells/mg
Frequency (%)
ns
**
ns
ns
**
ns
*
**
***
**
b
CD4 T cell distubution in major organs
Cells/mg
Frequency (%)
ns
ns
ns
*
ns
ns
ns
ns
**
ns
c
CD8 T cell distubution in major organs
Cells/mg
Frequency (%)
ns
*
ns
ns
ns
ns
*
ns
**
ns
Cre-
Cre+
B Cells
CD4 T Cells
CD8 T Cells

Supplementary Fig. 5. Lymphoid cell distribution in major organs of *Csf1r*$^{Cre+}$*; Wnk1*$^{fl/fl}$ mice.

(a-c) Flow cytometry analysis of the lymphoid compartment isolated from experiments performed in **Fig. 1c, d**. Shown are summary plots of absolute numbers per milligram of tissue (top graphs) and frequencies (bottom graphs) of (**a**) B cells (CD19+ MHCII+ CD4- CD8- CD11b- CD45+), (**b**) CD4+ T cells (CD4+ CD8- CD19- CD11b- CD11c- CD45+), and (**c**) CD8+ T cells (CD8+ CD4- CD19- CD11b- CD11c- CD45+). FACS gating was first performed as shown in Supplementary Fig. 2a. Data are from six *Csf1r*$^{Cre+}$; *Wnk1*$^{fl/fl}$ mice and six littermate controls. Data are shown as mean ± SEM. Statistical significance was determined via independent samples *t*-test. *$p < .05$, **$p < .01$, ***$p < .001$, ns = not significant.

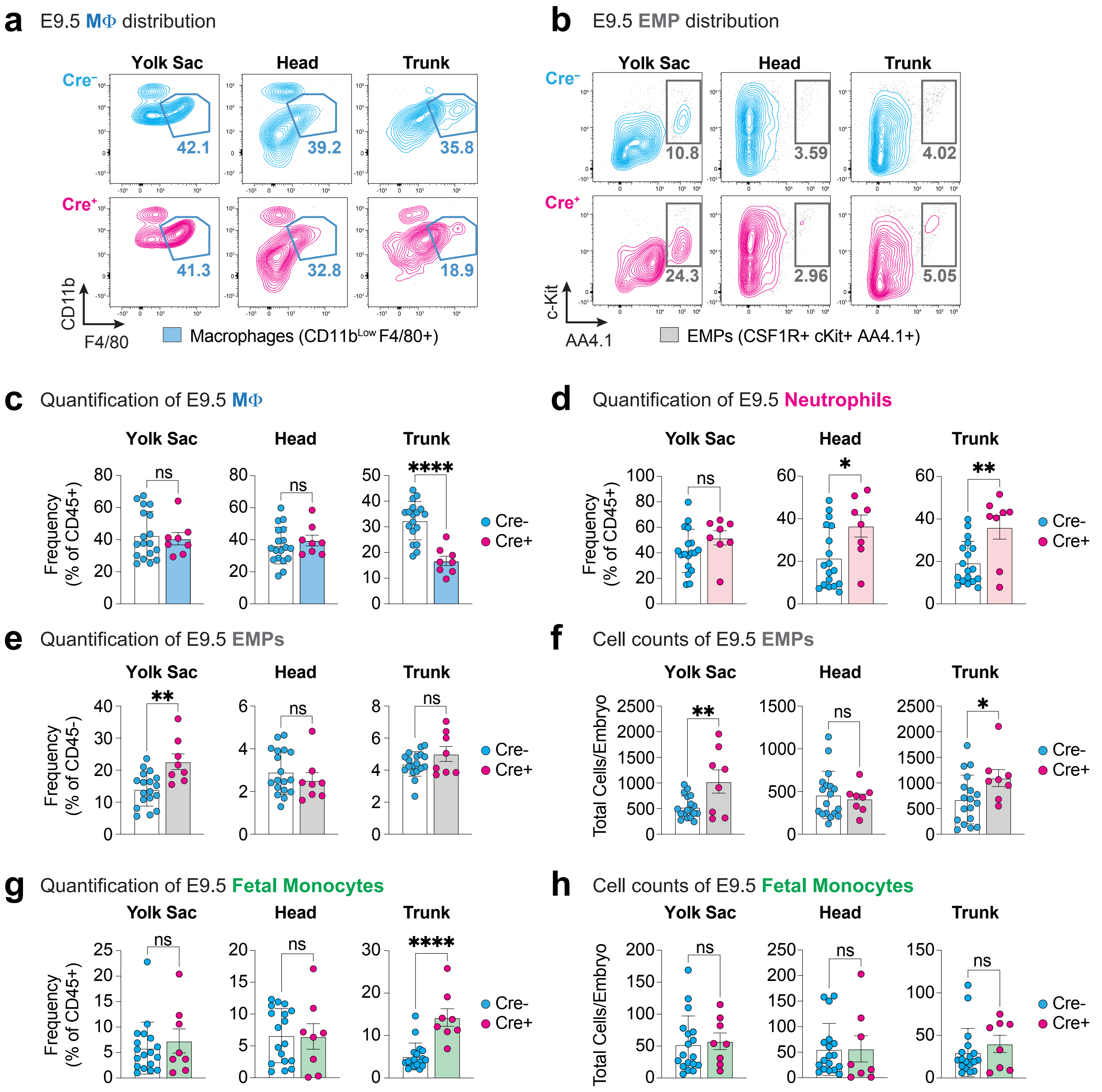
a
E9.5 MΦ distribution
Yolk Sac
Head
Trunk
Cre⁻
Cre⁺
42.1
39.2
35.8
41.3
32.8
18.9
CD11b
F4/80
Macrophages (CD11bLow F4/80+)
b
E9.5 EMP distribution
10.8
3.59
4.02
24.3
2.96
5.05
c-Kit
AA4.1
EMPs (CSF1R+ cKit+ AA4.1+)
c
Quantification of E9.5 MΦ
d
Quantification of E9.5 Neutrophils
Frequency (% of CD45+)
ns
****
*
**
Cre-
Cre+
e
Quantification of E9.5 EMPs
Frequency (% of CD45-)
f
Cell counts of E9.5 EMPs
Total Cells/Embryo
g
Quantification of E9.5 Fetal Monocytes
h
Cell counts of E9.5 Fetal Monocytes

Supplementary Fig. 6. ***Csf1r^{Cre+}; Wnk1$^{fl/fl}$*** **E9.5 embryo characterization**.

(a, b) Analysis of *Csf1r$^{+/+}$; Wnk1$^{fl/fl}$* and *Csf1r$^{Cre/+}$; Wnk1$^{fl/fl}$* E9.5 embryos. Representative flow plots of embryonic macrophages (**a**) and erythromyeloid progenitors (EMPs; **b**) in the yolk sac, head, and trunk regions of Cre- embryos and Cre+ embryos at E9.5 of macrophages as CD11b^{Low} F4/80+ cells and EMPs as CSF1R+ cKit+ AA4.1+ cells for analysis in **Fig. 1e, f** and Supplementary Fig. 6c-h.Timed pregnancies were performed with Csf1rCre/+; Wnk1fl/+ mice bred to Csf1r+/+; Wnk1fl/fl mice.

(c-h) Flow cytometric analysis of embryonic macrophages, fetal neutrophils, erythromyeloid progenitors (EMPs), and fetal monocytes in E9.5 embryos. 8 pregnant dams were euthanized at E9.5 and embryos were isolated, genotyped, and analyzed. Shown are summary plots of the frequencies (and absolute counts in **f** and **h**) from flow cytometry analysis CD11b^{Low} F4/80+ macrophages (**c**), CD11b^{High} Ly6C+ Ly6G+ fetal neutrophils (**d**)**,** Lin- CSF1R+ cKit+ AA4.1 + EMPs (**e, f**), and CD11b^{High} Ly6C+ fetal monocytes (**g, h**) in Cre- (n=18, blue dots) and Cre+ (n=8, magenta dots) from E9.5 embryos harvested as in **Fig. 1e, f**. FACS gating was first performed as shown in Supplementary Fig. 2a. Pooled data are from 8 independent experiments with between two and six biological replicates per experiment. Data shown as mean ± SEM. Statistical significance was determined via independent samples t-test. *p < .05, **p < .01, ****p < .0001, ns = not significant.

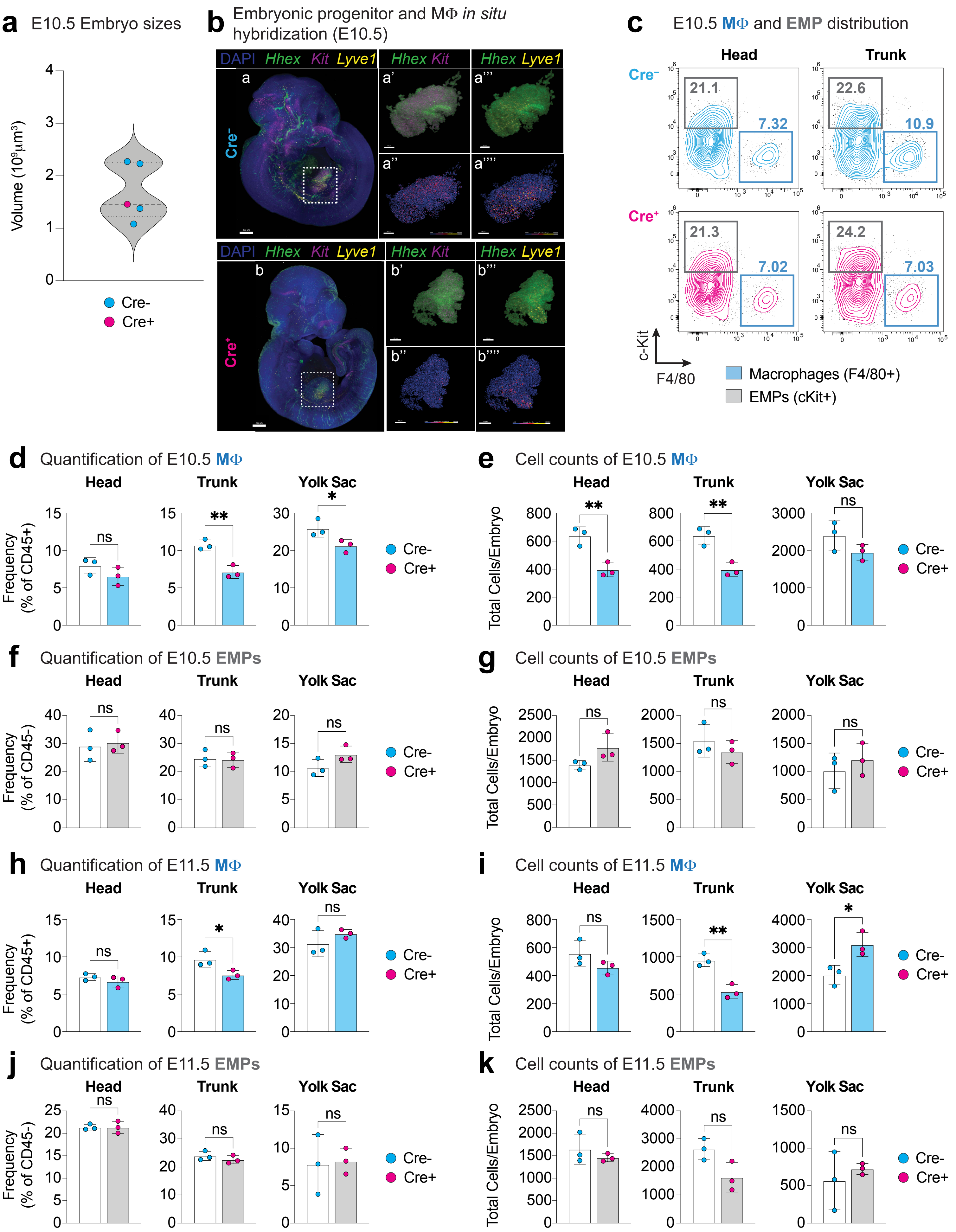

a E10.5 Embryo sizes
Volume (10⁹µm³)
Cre-
Cre+
b Embryonic progenitor and MΦ in situ hybridization (E10.5)
DAPI Hhex Kit Lyve1
Hhex Kit
Hhex Lyve1
Cre⁻
Cre⁺
c E10.5 MΦ and EMP distribution
Head
Trunk
21.1
7.32
22.6
10.9
21.3
7.02
24.2
7.03
c-Kit
F4/80
Macrophages (F4/80+)
EMPs (cKit+)
d Quantification of E10.5 MΦ
e Cell counts of E10.5 MΦ
f Quantification of E10.5 EMPs
g Cell counts of E10.5 EMPs
h Quantification of E11.5 MΦ
i Cell counts of E11.5 MΦ
j Quantification of E11.5 EMPs
k Cell counts of E11.5 EMPs
Head
Trunk
Yolk Sac
Frequency (% of CD45+)
Frequency (% of CD45-)
Total Cells/Embryo
ns
*
**

Supplementary Fig. 7. *Csf1r^{Cre+}; Wnk1$^{fl/fl}$* E10.5 and E11.5 embryo characterization.

(a, b) Analysis of *Csf1r$^{+/+}$; Wnk1$^{fl/fl}$* and *Csf1r$^{Cre/+}$; Wnk1$^{fl/fl}$* E10.5 embryos. Timed pregnancies were performed with *Csf1r$^{Cre/+}$; Wnk1$^{fl/+}$* mice bred to *Csf1r$^{+/+}$; Wnk1$^{fl/fl}$* mice. (**a**) Embryonic size variation as measured in volume ($10^9 \mu m^3$) at E10.5. Cre+ embryo (magenta dot) represented in Supplementary Fig. 7b-g falls within the Cre- E10.5 embryo (blue) normal size variation. (**b**) 4 pregnant dams were euthanized at E10.5 and embryos were isolated and analyzed using RNA probes identifying erythromyeloid progenitors (*Kit*, purple), macrophages (*Lyve1*, yellow), and fetal liver (*Hhex*, green). Shown are representative images of E10.5 embryos from *Csf1r$^{+/+}$; Wnk1$^{fl/fl}$* (Cre-; top) and *Csf1r$^{Cre/+}$; Wnk1$^{fl/fl}$* (Cre+; bottom). Highlighted regions are of the fetal liver (' to '''), the earliest tissue to feature yolk sac-derived erythromyeloid progenitor (EMP) differentiation into tissue-resident macrophages, illustrating the dearth of macrophage presence in Cre+ mice. Data in (a) is from one representative litter**.** FACS gating was first performed as shown in Supplementary Fig. 2a.

(c) Representative flow plots of embryonic macrophages and erythromyeloid progenitors in the head and trunk regions of Cre- embryos (blue plots) and Cre+ embryos (magenta plots) at E10.5 as cKit+ cells (grey) and macrophages as F4/80+ cells (blue) of analysis in Supplementary Fig. 7d-k. FACS gating was first performed as shown in Supplementary Fig. 2a.

(d-g) Flow cytometric analysis of embryonic macrophages and EMPs in E10.5 embryos. Shown are summary plots of frequencies and absolute numbers from flow cytometry analysis of F4/80+ macrophages **(d, e)** and Lin- cKit+ EMPs **(f, g)** in Cre+ (n=3, magenta dots) and Cre- (n=3, blue dots) from E10.5 embryos harvested as in Extended Data Fig. 7b. Data shown with three biological replicates per experiment. Data are shown as mean ± SEM. Statistical significance was determined via independent samples *t*-test. $*p < .05$, $**p < .01$, ns = not significant.

(h-k) Flow cytometric analysis of embryonic macrophages and EMPs in E11.5 embryos. Shown are summary plots of frequencies and absolute numbers from flow cytometry analysis of F4/80+ macrophages **(h, i)** and Lin- cKit+ EMPs **(j, k)** in Cre+ (n=3, magenta dots) and Cre- (n=3, blue dots) from E11.5 embryos harvested similar to Supplementary Fig. 7b. Data shown with three biological replicates per experiment. Data are shown as mean ± SEM. Statistical significance was determined via independent samples *t*-test. $*p < .05$, $**p < .01$, ns = not significant.

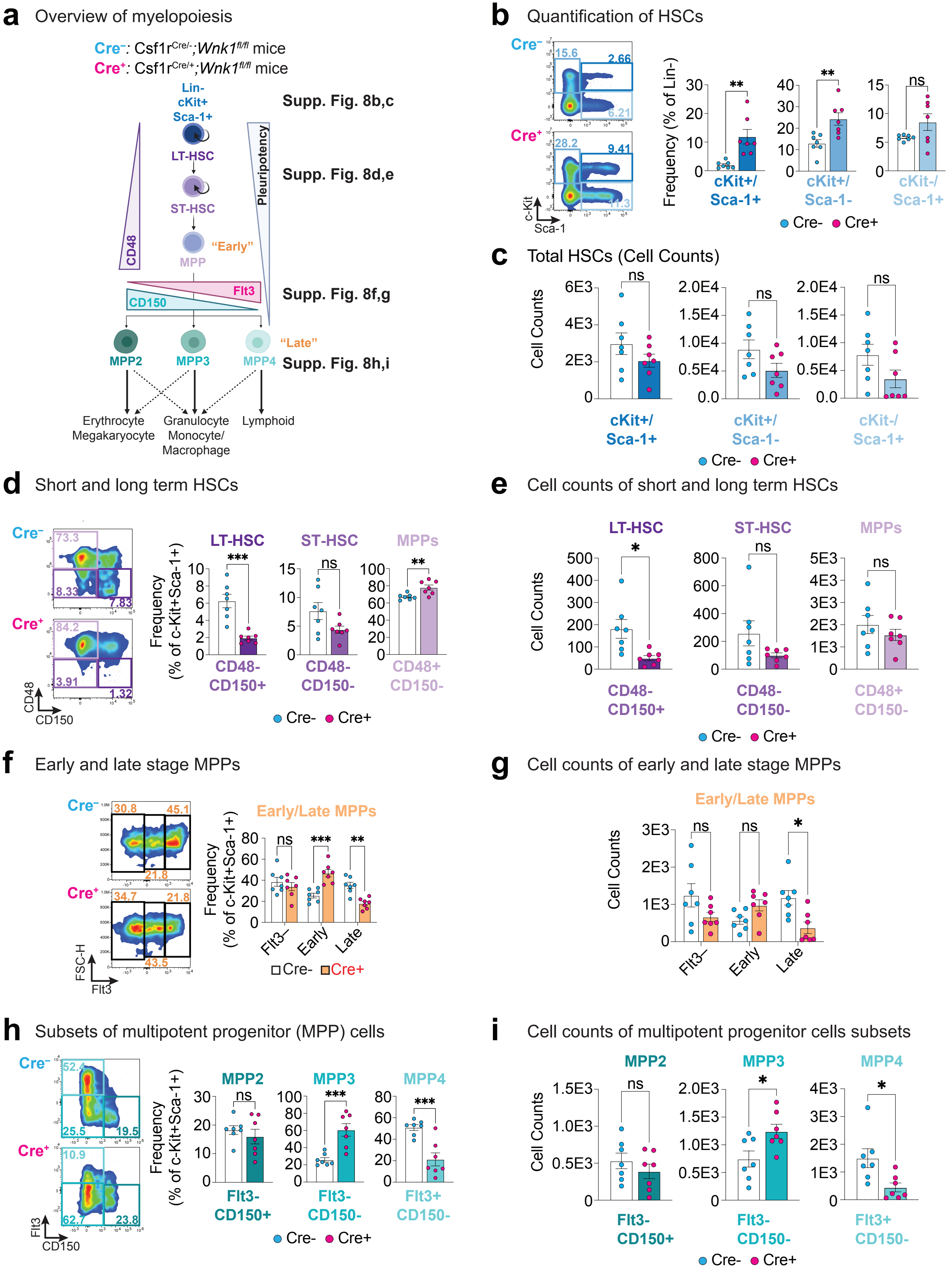
a Overview of myelopoiesis
Cre⁻: Csf1r Cre/-;Wnk1 fl/fl mice
Cre⁺: Csf1r Cre/+;Wnk1 fl/fl mice
Lin- cKit+ Sca-1+
LT-HSC
ST-HSC
MPP
"Early"
"Late"
CD48
Pleuripotency
Flt3
CD150
MPP2
MPP3
MPP4
Erythrocyte Megakaryocyte
Granulocyte Monocyte/ Macrophage
Lymphoid
Supp. Fig. 8b,c
Supp. Fig. 8d,e
Supp. Fig. 8f,g
Supp. Fig. 8h,i
b Quantification of HSCs
Frequency (% of Lin-)
cKit+/ Sca-1+
cKit+/ Sca-1-
cKit-/ Sca-1+
c-Kit
Sca-1
Cre- Cre+
c Total HSCs (Cell Counts)
Cell Counts
d Short and long term HSCs
LT-HSC
ST-HSC
MPPs
Frequency (% of c-Kit+Sca-1+)
CD48- CD150+
CD48- CD150-
CD48+ CD150-
CD48
CD150
e Cell counts of short and long term HSCs
f Early and late stage MPPs
Early/Late MPPs
Flt3–
Early
Late
FSC-H
Flt3
g Cell counts of early and late stage MPPs
h Subsets of multipotent progenitor (MPP) cells
Flt3- CD150+
Flt3- CD150-
Flt3+ CD150-
i Cell counts of multipotent progenitor cells subsets

Supplementary Fig. 8. Analysis of hematopoietic stem and progenitor cell types in *Csf1r*$^{Cre/+}$*; Wnk1*$^{fl/fl}$ mice

(a) Schematic of hematopoiesis with long-term (LT), short-term (ST), and multipotent progenitors (MPPs) with corresponding markers. Also shown is the figure panel that corresponds to the lineage state analyzed. Where possible, bar graph color corresponds to cell color in the schematic. FACS gating was first performed as shown in Supplementary Fig. 2a.

(b, c) Quantification of hematopoietic stem cell (HSC) cKit and Sca-1 expression and cell count. (**b**) Representative FACS (*left*) and summary (*right*) plots of cKit and Sca-1 expression in HSCs and (**c**) cell counts of different HSC populations. Bone marrow cells were isolated from three-to-four-week-old mice and were first gated on lineage negative (Lin-) cells, then analyzed for cKit and Sca-1 positivity. Data are from n=7 *Csf1*$^{+/+}$*; Wnk1*$^{fl/fl}$ (Cre-, blue dots) and n=7 *Csf1r*$^{Cre/+}$*; Wnk1*$^{fl/fl}$ (Cre+, magenta dots) mice across four independent experiments. Data are shown as mean± SEM. Statistical significance was determined via independent samples t-test. **p < .01, ns = not significant.

(d, e) Quantification of LT-HSC, ST-HSC, and early MPP populations. (**d**) Representative FACS (*left*) and summary (*right*) plots of the HSC (defined as Lin- cKit+ Sca-1+) subtypes long-term (LT: CD48- CD150+, dark purple bar), short-term (ST: CD48- CD150-, purple bar), and multipotent progenitor (MPP: CD48+ CD150-, light purple bar) and (**e**) cell counts. Data are from n=7 Cre- (blue dots) and n=7 Cre+ (magenta dots) mice across four independent experiments. Data are shown as mean ± SEM. Statistical significance was determined via independent samples t-test. *p < .05, **p < .01, ***p < .001, ns = not significant.

(f, g) Quantification of early and late stage MPPs. (**f**) Representative FACS (*left*) and summary (*right*) plots of Flt3 negative (Flt3^{-}), early (Flt3^{low}), and late (Flt3^{high}) MPPs and (**g**) cell counts. Cells were initially gated as CD48+ CD150-. Data are from n=7 Cre- (blue dots) and n=7 Cre+ (magenta dots) mice across four independent experiments. Data are shown as mean ± SEM. Statistical significance was determined via one-way ANOVA. *p < .05, **p < .01, ***p < .001, ns = not significant.

(h, i) Quantification of MPP subsets. (**h**) Representative FACS (*left*) and summary (*right*) plots of the following MPP subsets: MPP2 (erythrocyte/megakaryocyte lineage, dark green bar), MMP3 (granulocyte/monocyte/macrophage lineage, green bar), and MPP4 (lymphoid lineage, light green bar) and (**i**) cell counts. Data are from n=7 Cre- (blue dots) and n=7 Cre+ (magenta dots) mice across four independent experiments. Data are shown as mean ± SEM. Statistical significance was determined via independent samples t-test. *p < .05, ***p < .001, ns = not significant.

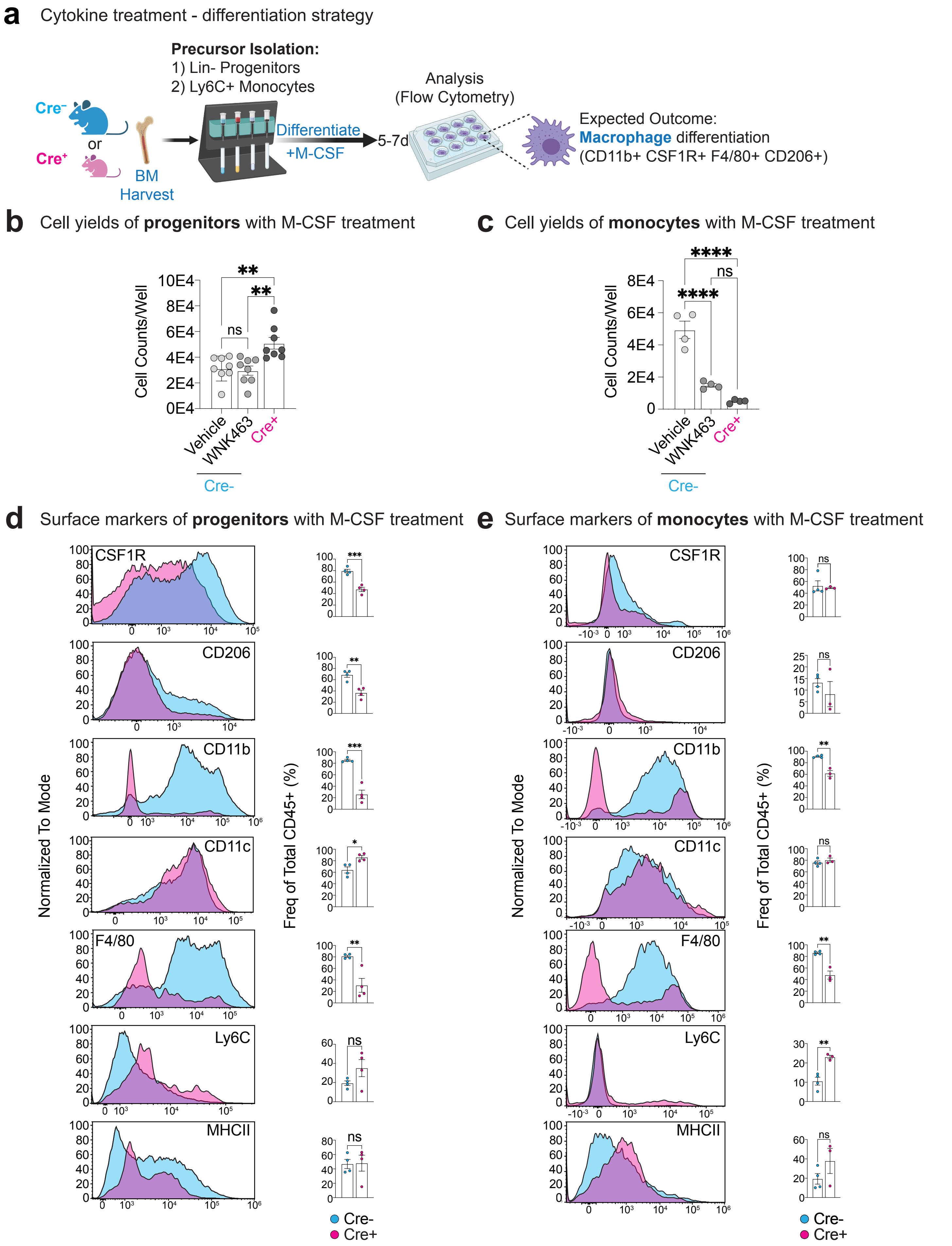
a
Cytokine treatment - differentiation strategy
Cre⁻
or
Cre⁺
BM Harvest
Precursor Isolation:
1) Lin- Progenitors
2) Ly6C+ Monocytes
Differentiate +M-CSF
5-7d
Analysis (Flow Cytometry)
Expected Outcome:
Macrophage differentiation
(CD11b+ CSF1R+ F4/80+ CD206+)
b
Cell yields of progenitors with M-CSF treatment
Cell Counts/Well
10E4
8E4
6E4
4E4
2E4
0E4
**
**
ns
Vehicle
WNK463
Cre+
Cre-
c
Cell yields of monocytes with M-CSF treatment
Cell Counts/Well
8E4
6E4
4E4
2E4
0
****
ns
****
Vehicle
WNK463
Cre+
Cre-
d
Surface markers of progenitors with M-CSF treatment
Normalized To Mode
CSF1R
CD206
CD11b
CD11c
F4/80
Ly6C
MHCII
Freq of Total CD45+ (%)
***
**
***
*
**
ns
ns
Cre-
Cre+
e
Surface markers of monocytes with M-CSF treatment
Normalized To Mode
CSF1R
CD206
CD11b
CD11c
F4/80
Ly6C
MHCII
Freq of Total CD45+ (%)
ns
ns
**
ns
**
**
ns
Cre-
Cre+

Supplementary Fig. 9. ***Csf1r*$^{Cre+}$*; Wnk1*$^{fl/fl}$ cells do not become canonical macrophages.**

(a) Shown is the experimental strategy used to test macrophage differentiation of bone marrow Lin- progenitors or monocytes from *Csf1r*$^{Cre+}$*; Wnk1*$^{fl/fl}$ (Cre+, blue) or *Csf1*$^{Cre-}$*; Wnk1*$^{fl/fl}$ (Cre- magenta) mice in response to M-CSF.

(b, c) Analysis of total cell yields from M-CSF-treated mouse bone marrow (**b**) bone marrow Lin- progenitors and (**c**) Ly6C+ monocytes from **Fig. 2a, b**. Data are representative of three independent experiments. Summary plots are shown as mean ± SEM. Statistical significance was assessed via independent samples *t*-test. $^{**}p < .01$, $^{****}p < .0001$, ns = not significant.

(d, e) Representative flow cytometry plots and summary data of CSF1R, CD206, CD11b, CD11c, F4/80, Ly6C, and MHCII in bone marrow Lin- progenitors (**b**) or Ly6C+ monocytes (**c**) treated with M-CSF as in (**a**). Data are representative of three independent experiments and shown as mean ± SEM. Statistical significance was determined via independent samples *t*-test. $^{*}p < .05$, $^{**}p < .01$, $^{***}p < .001$, ns = not significant. FACS gating was first performed as shown in Supplementary Fig. 2a.

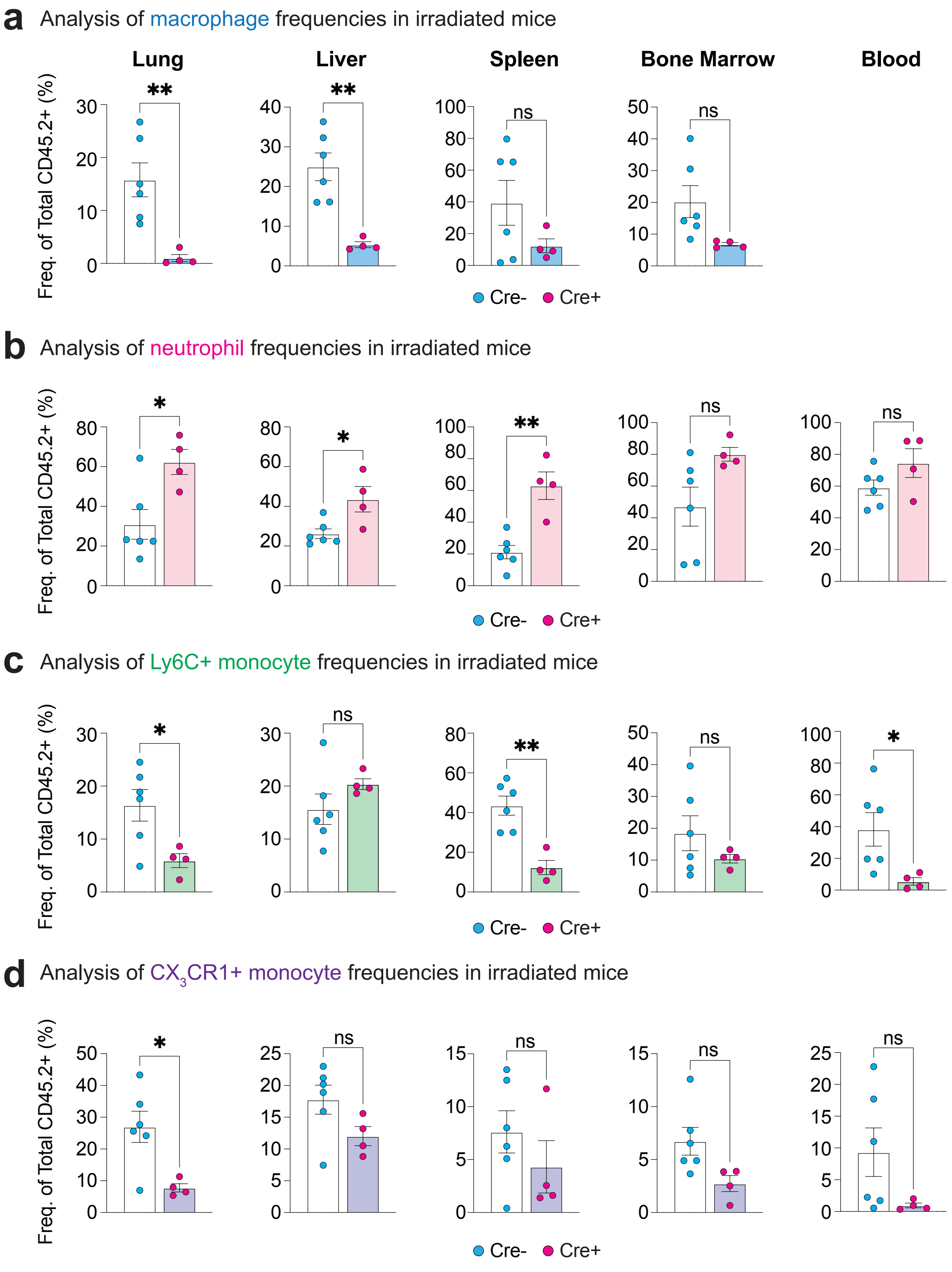
a Analysis of macrophage frequencies in irradiated mice
Lung
Liver
Spleen
Bone Marrow
Blood
Freq. of Total CD45.2+ (%)
**
**
ns
ns
Cre-
Cre+
b Analysis of neutrophil frequencies in irradiated mice
Freq. of Total CD45.2+ (%)
*
*
**
ns
ns
Cre-
Cre+
c Analysis of Ly6C+ monocyte frequencies in irradiated mice
Freq. of Total CD45.2+ (%)
*
ns
**
ns
*
Cre-
Cre+
d Analysis of CX_3CR1+ monocyte frequencies in irradiated mice
Freq. of Total CD45.2+ (%)
*
ns
ns
ns
ns
Cre-
Cre+

Supplementary Fig. 10. Myeloid cell frequencies in bone marrow transplantation experiments.

(a-d) Analysis of **(a)** macrophages, **(b)** neutrophils, **(c)** Ly6C+ monocytes, and **(d)** CX_3CR1+ monocytes arising from Cre- (Cre- CD45.2+ donor progenitors into wildtype CD45.1+ adult mice; blue dots) and Cre+ (Cre+ CD45.2+ donor progenitors into wildtype CD45.1+ adult mice; magenta dots, shaded bars) across tissues analyzed as in **Fig. 2d, e**. Data are pooled from two independent experiments, with three (Cre-, n=6 total) or two (Cre+, n=4 total) mice per group. Data are shown as mean ± SEM. Statistical significance was determined via independent samples *t*-test. *$p < .05$, **$p < .01$, ns = not significant. FACS gating was first performed as shown in Supplementary Fig. 2a.

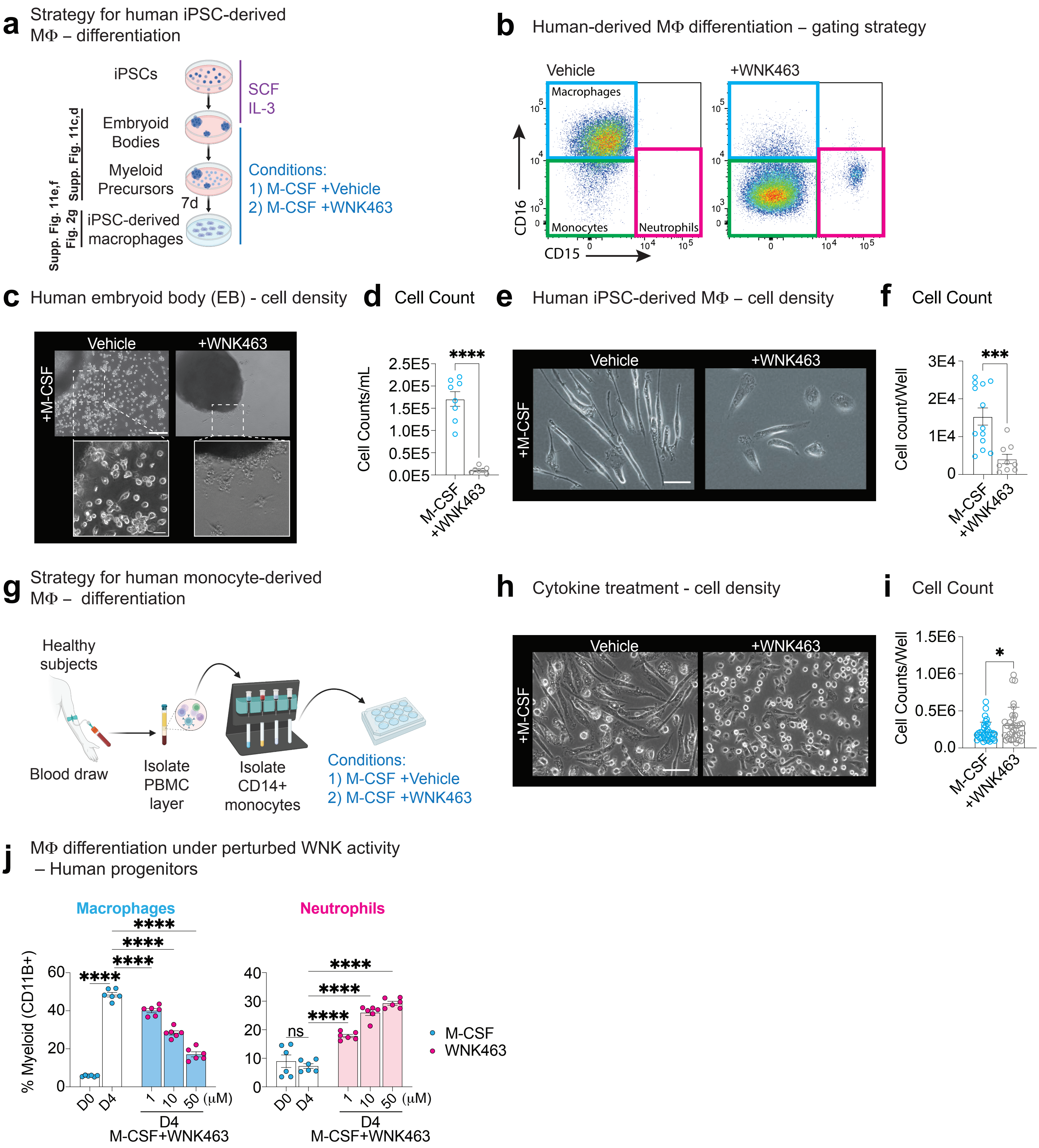
a
Strategy for human iPSC-derived MΦ – differentiation
iPSCs
SCF
IL-3
Embryoid Bodies
Myeloid Precursors
7d
iPSC-derived macrophages
Conditions:
1) M-CSF +Vehicle
2) M-CSF +WNK463
Supp. Fig. 11c,d
Supp. Fig. 11e,f
Fig. 2g
b
Human-derived MΦ differentiation – gating strategy
Vehicle
+WNK463
Macrophages
Monocytes
Neutrophils
CD16
CD15
c
Human embryoid body (EB) - cell density
Vehicle
+WNK463
+M-CSF
d
Cell Count
Cell Counts/mL
2.5E5
2.0E5
1.5E5
1.0E5
0.5E5
0.0E5
****
M-CSF
+WNK463
e
Human iPSC-derived MΦ – cell density
Vehicle
+WNK463
+M-CSF
f
Cell Count
Cell count/Well
3E4
2E4
1E4
0
***
M-CSF
+WNK463
g
Strategy for human monocyte-derived MΦ – differentiation
Healthy subjects
Blood draw
Isolate PBMC layer
Isolate CD14+ monocytes
Conditions:
1) M-CSF +Vehicle
2) M-CSF +WNK463
h
Cytokine treatment - cell density
Vehicle
+WNK463
+M-CSF
i
Cell Count
Cell Counts/Well
1.5E6
1.0E6
0.5E6
0.0
*
M-CSF
+WNK463
j
MΦ differentiation under perturbed WNK activity – Human progenitors
Macrophages
Neutrophils
% Myeloid (CD11B+)
****
ns
D0
D4
1
10
50
(µM)
D4
M-CSF+WNK463
M-CSF
WNK463

Supplementary Fig. 11. Human cells do not differentiate into macrophages in the presence of WNK463.

(a, b) Analysis of human pluripotent stem cell (iPSC)-derived macrophage differentiation in response to M-CSF. Shown is the experimental strategy (**a**) and flow cytometry gating strategy (**b**) used to test the effect of inhibition of WNK kinase activity (via WNK463) on differentiation of human iPSCs into myeloid progenitors and myeloid progenitors into macrophages. Also shown is the figure panel that corresponds to the state of differentiation being analyzed. Human macrophages were quantified based on expression of CD11B+, $CD14^{Low}$, $CD16^{High}$, CSF1R+, CD206+, and CD86+. Human monocytes were quantified based on expression of CD11B+, $CD14^{High}$, $CD16^{Low}$, CD206-, and CD86-. Human neutrophils were gated based on expression of CD11B+, $CD16^{mid}$, $CD14^{Low}$, CD15+, and CD66B+.

(c, d) Perturbation of WNK kinase activity blocks maturation of human embryoid bodies (EBs) into myeloid progenitors. Representative images **(c)** and quantitation **(d)** of myeloid progenitor numbers from experiments performed as detailed in Extended Data Fig. 11a. Pooled data are from four independent experiments with one-to-two biological replicates (unique donors) per experiment. Data are shown as mean ± SEM. Statistical significance was determined via independent samples *t*-test. $^{****}p < .0001$. Scale bar, 150μm.

(e, f) Perturbation of WNK kinase activity prevents differentiation of human EMPs into mature macrophages. Representative images **(e)** and quantitation of total cell numbers **(f)** as detailed in **Fig. 2g**. Pooled data are from four independent experiments with one-to-two biological replicates (unique donors) per experiment. Data are shown as mean ± SEM. Statistical significance was determined via independent samples *t*-test. $^{***}p < .001$. Scale bar, 20μm. See also Extended Data Fig. 12a for flow cytometry characterization of cell-surface proteins.

(g) Analysis of human blood monocyte-derived macrophage differentiation in response to M-CSF. Shown is the experimental strategy used to test the effect of WNK protein function inhibition (via WNK463) on differentiation of human blood monocytes into macrophages.

(h, i) Perturbation of WNK function blocks maturation of human blood monocytes into mature macrophages. Representative images **(h)** and quantitation of total cell numbers **(i)** as detailed in **Fig. 2h**. Pooled data are from four independent experiments with two biological replicates (unique male and female donors) per experiment. Data are shown as mean ± SEM. Statistical significance was determined via independent samples *t*-test. $^{*}p < .05$. Scale bar, 20μm. See also Extended Data Fig. 12b for flow cytometry characterization of cell-surface proteins.

(j) Experiments were performed as in Extended Data Fig. 11e and f, except using a dose titration curve of WNK463 up to the highest tolerable dose (50 μM). Pooled data are from four independent experiments with one-to-two biological replicates (unique donors) per experiment. Data are shown as mean ± SEM. Statistical significance was determined via independent samples *t*-test. $^{****}p < .0001$.

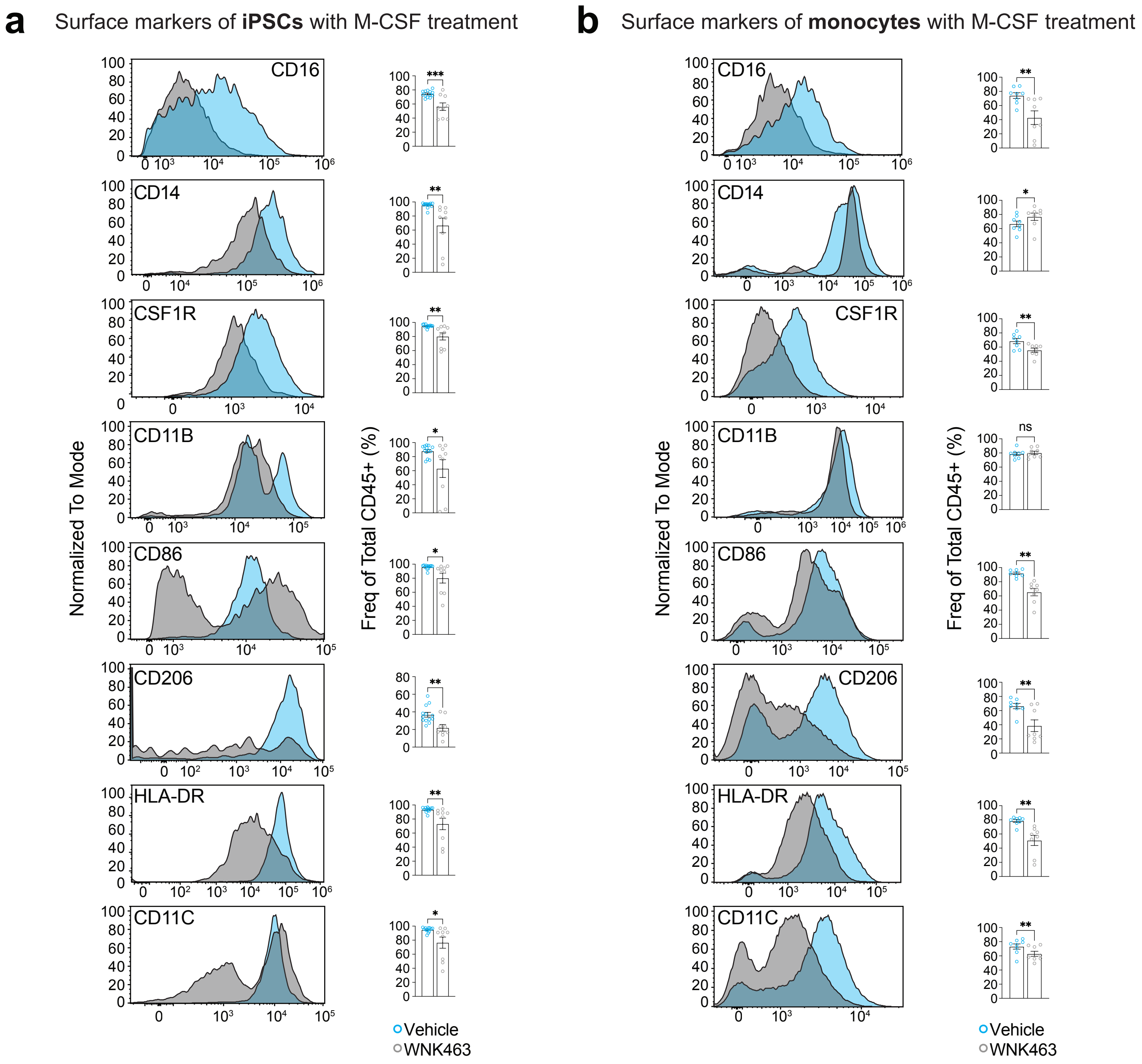

a
Surface markers of iPSCs with M-CSF treatment
b
Surface markers of monocytes with M-CSF treatment
CD16
CD14
CSF1R
CD11B
CD86
CD206
HLA-DR
CD11C
Normalized To Mode
Freq of Total CD45+ (%)
Vehicle
WNK463

Supplementary Fig. 12. Human myeloid progenitors and monocytes do not differentiate into macrophages in the presence of WNK463.

(a, b) Representative histograms and summary plots for flow cytometry analysis of human cell surface markers in iPSC-derived myeloid progenitors **(a)** and in human monocytes **(b)** treated with human M-CSF in the presence of vehicle or WNK463 as detailed in **Fig. 2g, h**. Pooled data are from four independent experiments with (**a**) two-three or (**b**) one-to-two biological replicates (unique donors) per experiment. Data are shown as mean ± SEM. Statistical significance was determined via independent samples *t*-test. *$p < .05$, **$p < .01$, ***$p < .001$, ns = not significant. FACS gating was first performed as shown in Supplementary Fig. 11b.

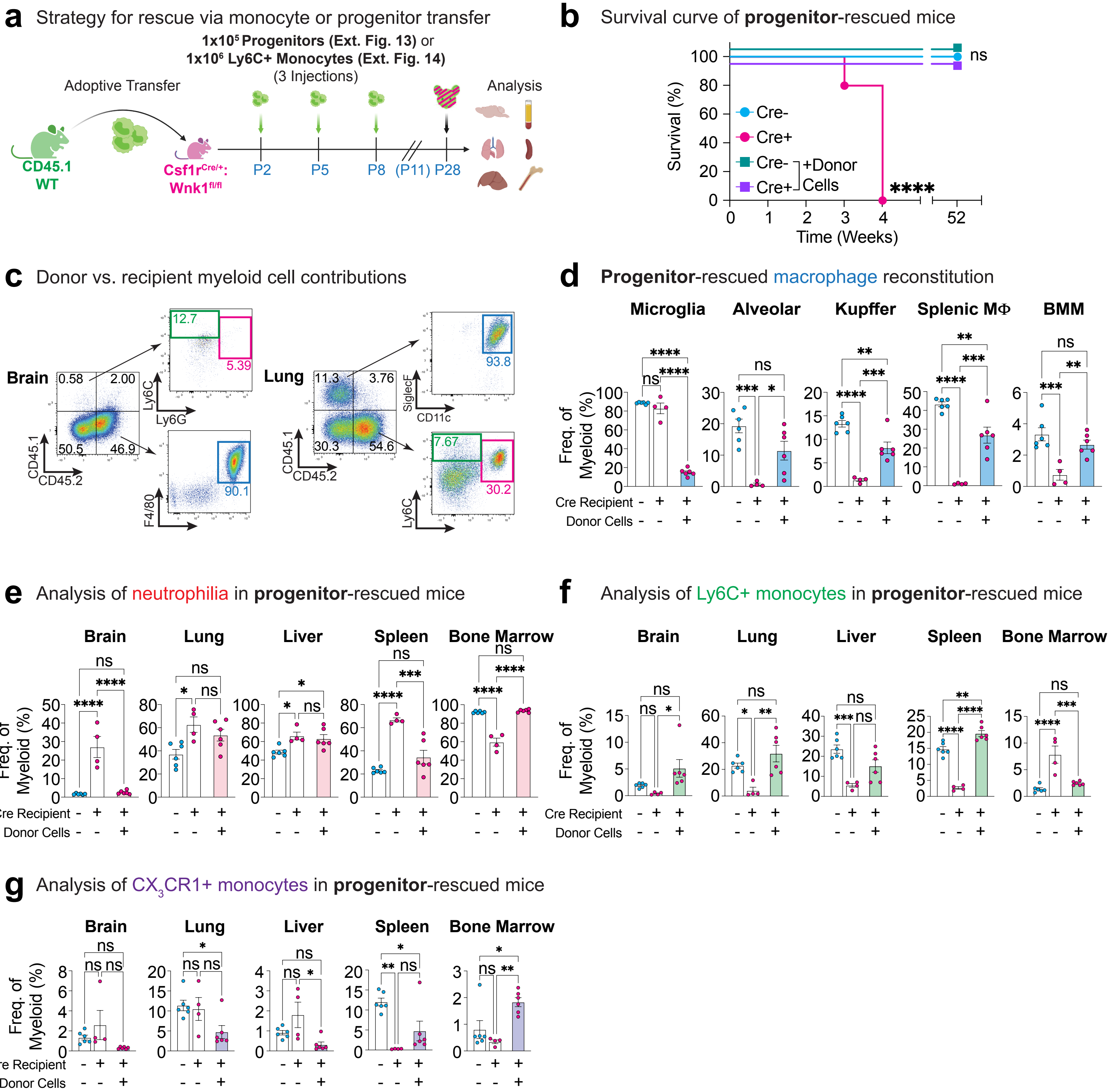

a Strategy for rescue via monocyte or progenitor transfer
1x10⁵ Progenitors (Ext. Fig. 13) or
1x10⁶ Ly6C+ Monocytes (Ext. Fig. 14)
(3 Injections)
Adoptive Transfer
Analysis
CD45.1 WT
Csf1rCre/+: Wnk1fl/fl
P2
P5
P8
(P11) P28
b Survival curve of progenitor-rescued mice
Survival (%)
Time (Weeks)
Cre-
Cre+
Cre-
Cre+
+Donor Cells
ns
****
c Donor vs. recipient myeloid cell contributions
Brain
Lung
d Progenitor-rescued macrophage reconstitution
Microglia
Alveolar
Kupffer
Splenic MΦ
BMM
Freq. of Myeloid (%)
Cre Recipient
Donor Cells
e Analysis of neutrophilia in progenitor-rescued mice
f Analysis of Ly6C+ monocytes in progenitor-rescued mice
g Analysis of CX3CR1+ monocytes in progenitor-rescued mice
Brain
Lung
Liver
Spleen
Bone Marrow

Supplementary Fig. 13. CD45.1 donor progenitor contribution in Cre+ recipient mice.

(a) Rescue of macrophage development via neonatal injection of bone marrow Lin- progenitors or bone marrow monocytes. Experimental strategy for adoptive transfer of wildtype bone marrow Lin- progenitors (Extended Data Fig. 13b-g) or bone marrow monocyte (Extended Data Fig. 14) into *Csf1r*$^{Cre+}$; *Wnk1*$^{fl/fl}$ (Cre+) or *Csf1*$^{Cre-}$; *Wnk1*$^{fl/fl}$ (Cre-) neonatal mice.

(b) Analysis of survival in mice from experiments using bone marrow Lin- progenitors as detailed in Extended Data Fig. 13a. Survival curve of Cre- mice (blue), Cre+ mice (magenta), Cre- mice injected with wildtype CD45.1+ bone marrow myeloid progenitors (green), and Cre+ mice injected with wildtype CD45.1+ bone marrow myeloid progenitors beginning on perinatal day (P)2 (purple). Mice were monitored daily from birth and euthanized if IACUC-approved criteria were met. Data are from two independent experiments with three mice per group. Differences in survival were determined using the Mantel-Cox test. ****$p < .0001$, ns = not significant.

(c) Representative flow cytometry plot of the brain (*left*) and lungs (*right*) from Cre+ recipient mice (CD45.2+) injected with CD45.1+ bone marrow myeloid progenitors as in (**a**).

(d) Analysis of macrophage reconstitution in mice from experiments using Lin- progenitors as detailed in Supplementary Fig. 13a. Summary plots of macrophage frequency as a fraction CD45+ CD11b+ (except lung) F4/80+ cells in the lungs, liver, spleen, and bone marrow of Cre- (blue dots, white bars), Cre+ (magenta dots, white bars), and CD45.1+ CD11b+ F4/80+ cells Cre+ mice injected with wildtype CD45.1+ bone marrow Lin- progenitors on P2 (magenta dots, blue bars). FACS gating was first performed as shown in Supplementary Fig. 2a.

Data are from two independent experiments with two-to-three mice per group. Data are shown as mean ± SEM. Statistical significance was determined via one-way ANOVA. *$p < .05$, **$p < .01$, ***$p < .001$, ****$p < .0001$, ns = not significant.

(e) Analysis of neutrophilia in mice from experiments using Lin- progenitors as detailed in Extended Data Fig. 13a. Shown are summary plots of cell frequency as a fraction of CD45+ CD11b+ Ly6C+ Ly6G+ cells in the brain, lungs, liver, spleen, and bone marrow of Cre- (blue dots, white bars), Cre+ (magenta dots, white bars) and CD45.1+ CD11b+ Ly6C+ Ly6G+ cell in Cre+ mice injected with wildtype CD45.1+ bone marrow myeloid progenitors (magenta dots, pink bars). Data are from two independent experiments with two-to-three mice per group. Data are shown as mean ± SEM. Statistical significance was determined via one-way ANOVA. *$p < .05$, ***$p < .001$, ****$p < .0001$, ns = not significant.

(f, g) Analysis of **(f)** Ly6C+ monocyte and **(g)** CX_3CR1+ monocyte frequencies from bone marrow Lin- progenitor transfer experiments as detailed in Extended Data Fig. 13a. **(f)** Shown are summary plots of cell frequency of CD45+ CD11b+ F4/80- Ly6C+ as a fraction of myeloid (CD45+ CD11b+) cells in the brain, lungs, liver, spleen, and bone marrow of Cre- (blue dots, white bar), Cre+ (magenta dots, white bar), and frequencies of CD45.1+ CD11b+ F4/80- Ly6C+ Cre+ mice injected with wildtype CD45.1+ bone marrow myeloid progenitors (magenta dots, green bars). **(g)** Shown are summary plots of cell frequency of CD45+ CD11b+ F4/80- Ly6C- CX_3CR1+ as a fraction of myeloid (CD45+ CD11b+) cells in the brain, lungs, liver, spleen, and bone marrow of Cre- (blue dots, white bar), Cre+ (magenta dots, white bar), and frequencies of CD45.1+ CD11b+ F4/80- Ly6C+ Cre+ mice injected with wildtype CD45.1+ bone marrow myeloid progenitors (magenta dots, purple bars). Data are from two independent experiments with two-to-three mice per group. Data are shown as mean ± SEM. Statistical significance was determined via one-way ANOVA. *$p < .05$, **$p < .01$, ns = not significant.

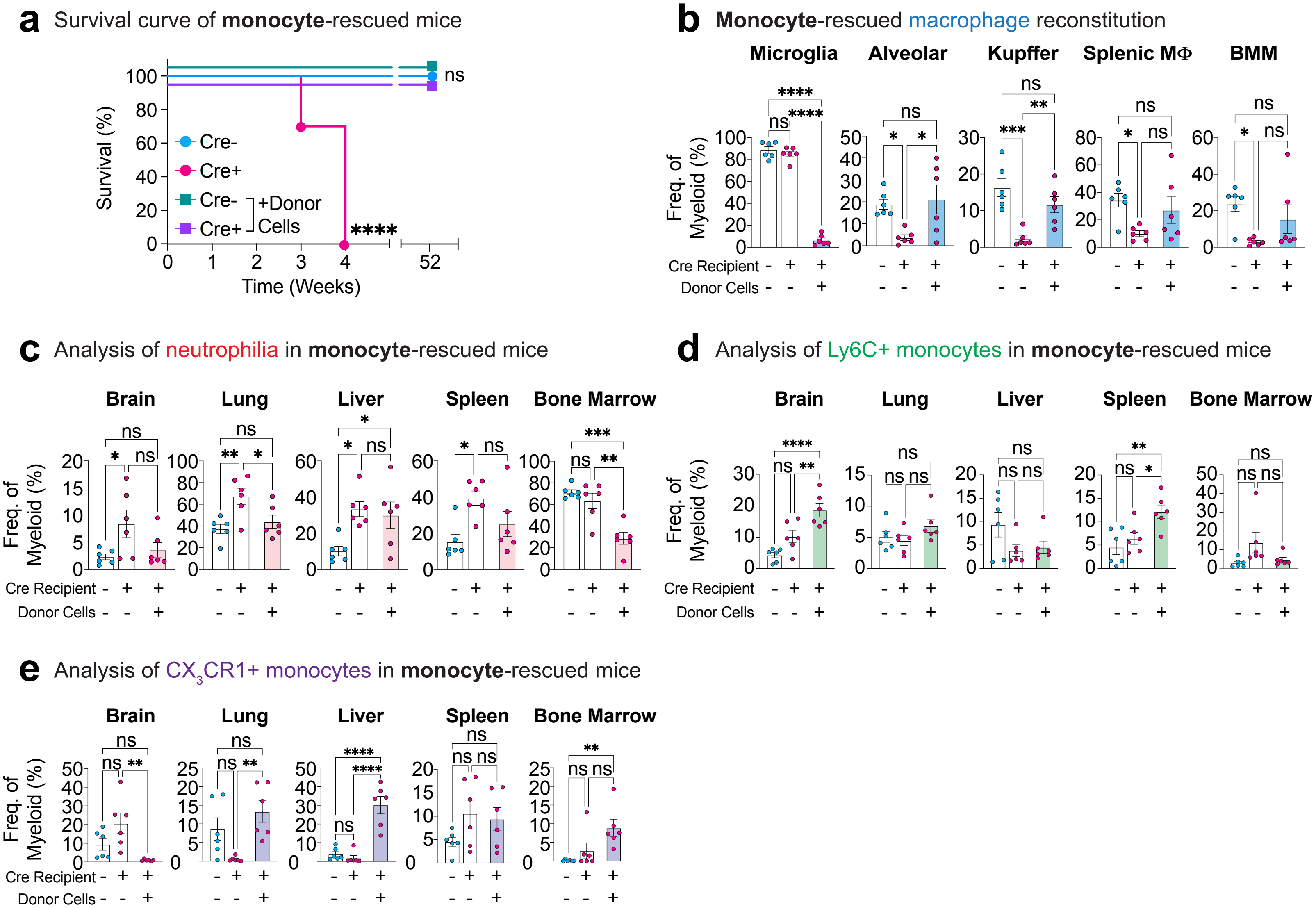
a
Survival curve of monocyte-rescued mice
Survival (%)
Time (Weeks)
Cre-
Cre+
Cre-
Cre+
+Donor Cells
ns
****
b
Monocyte-rescued macrophage reconstitution
Microglia
Alveolar
Kupffer
Splenic MΦ
BMM
Freq. of Myeloid (%)
Cre Recipient
Donor Cells
c
Analysis of neutrophilia in monocyte-rescued mice
Brain
Lung
Liver
Spleen
Bone Marrow
Freq. of Myeloid (%)
Cre Recipient
Donor Cells
d
Analysis of Ly6C+ monocytes in monocyte-rescued mice
Brain
Lung
Liver
Spleen
Bone Marrow
Freq. of Myeloid (%)
Cre Recipient
Donor Cells
e
Analysis of CX3CR1+ monocytes in monocyte-rescued mice
Brain
Lung
Liver
Spleen
Bone Marrow
Freq. of Myeloid (%)
Cre Recipient
Donor Cells

Supplementary Fig. 14. CD45.1 donor monocyte contribution in Cre+ recipient mice.

(a) Analysis of survival in mice from experiments using bone marrow monocytes as detailed in Supplementary Fig. 13a. Survival curve of Cre- mice (blue), Cre+ mice (magenta), Cre- mice injected with wildtype CD45.1+ bone marrow monocytes (green), and Cre+ mice injected with wildtype CD45.1+ bone marrow monocytes beginning on perinatal day (P)2 (purple). Mice were monitored daily from birth and euthanized if IACUC-approved criteria were met. Data are from two independent experiments with three mice per group. Differences in survival were determined using the Mantel-Cox test. ****$p < .0001$, ns = not significant.

(b) Analysis of macrophage reconstitution in mice from experiments using bone marrow Ly6C+ monocytes as detailed in Supplementary Fig. 13a. Summary plots of macrophage frequency as a fraction CD45+ CD11b+ (except lung) F4/80+ cells in the lungs, liver, spleen, and bone marrow of Cre- (blue dots, white bars), Cre+ (magenta dots, white bars), and CD45.1+ CD11b+ F4/80+ cells Cre+ mice injected with wildtype CD45.1+ bone marrow monocytes on P2 (magenta dots, blue bars). Data are from two independent experiments with three mice per group. Data are shown as mean ± SEM. Statistical significance was determined via one-way ANOVA.*$p < .05$, **$p < .01$, ***$p < .001$, ****$p < .0001$, ns = not significant. FACS gating was first performed as shown in Supplementary Fig. 2a.

(c) Analysis of neutrophilia in mice from experiments using bone marrow Ly6C+ monocytes as detailed in Supplementary Fig. 13a. Shown are summary plots of cell frequency as a fraction of CD45+ CD11b+ Ly6C+ Ly6G+ cells in the brain, lungs, liver, spleen, and bone marrow of Cre- (blue dots, white bars), Cre+ (magenta dots, white bars) and CD45.1+ CD11b+ Ly6C+ Ly6G+ cell in Cre+ mice injected with wildtype CD45.1+ bone marrow monocytes (magenta dots, pink bars). Data are from two independent experiments with three mice per group. Data are shown as mean ± SEM. Statistical significance was determined via one-way ANOVA. *$p < .05$, **$p < .01$, ***$p < .001$, ns = not significant.

(d, e) Analysis of **(d)** Ly6C+ monocyte and **(e)** CX_3CR1+ monocyte frequencies from bone marrow Ly6C+ monocyte transfer experiments as detailed in Supplementary Fig. 13a. **(d)** Shown are summary plots of cell frequency of CD45+ CD11b+ F4/80- Ly6C+ as a fraction of myeloid (CD45+ CD11b+) cells in the brain, lungs, liver, spleen, and bone marrow of Cre- (blue dots, white bar), Cre+ (magenta dots, white bar), and frequencies of CD45.1+ CD11b+ F4/80- Ly6C+ Cre+ mice injected with wildtype CD45.1+ bone marrow monocytes (magenta dots, green bars). **(e)** Shown are summary plots of cell frequency of CD45+ CD11b+ F4/80- Ly6C- CX_3CR1+ as a fraction of myeloid (CD45+ CD11b+) cells in the brain, lungs, liver, spleen, and bone marrow of Cre- (blue dots, white bar), Cre+ (magenta dots, white bar), and frequencies of CD45.1+ CD11b+ F4/80- Ly6C+ Cre+ mice injected with wildtype CD45.1+ bone marrow monocytes (magenta dots, purple bars). Data are from two independent experiments with three mice per group. Data are shown as mean ± SEM. Statistical significance was determined via one-way ANOVA. *$p < .05$, **$p < .01$, ****$p < .0001$, ns = not significant.

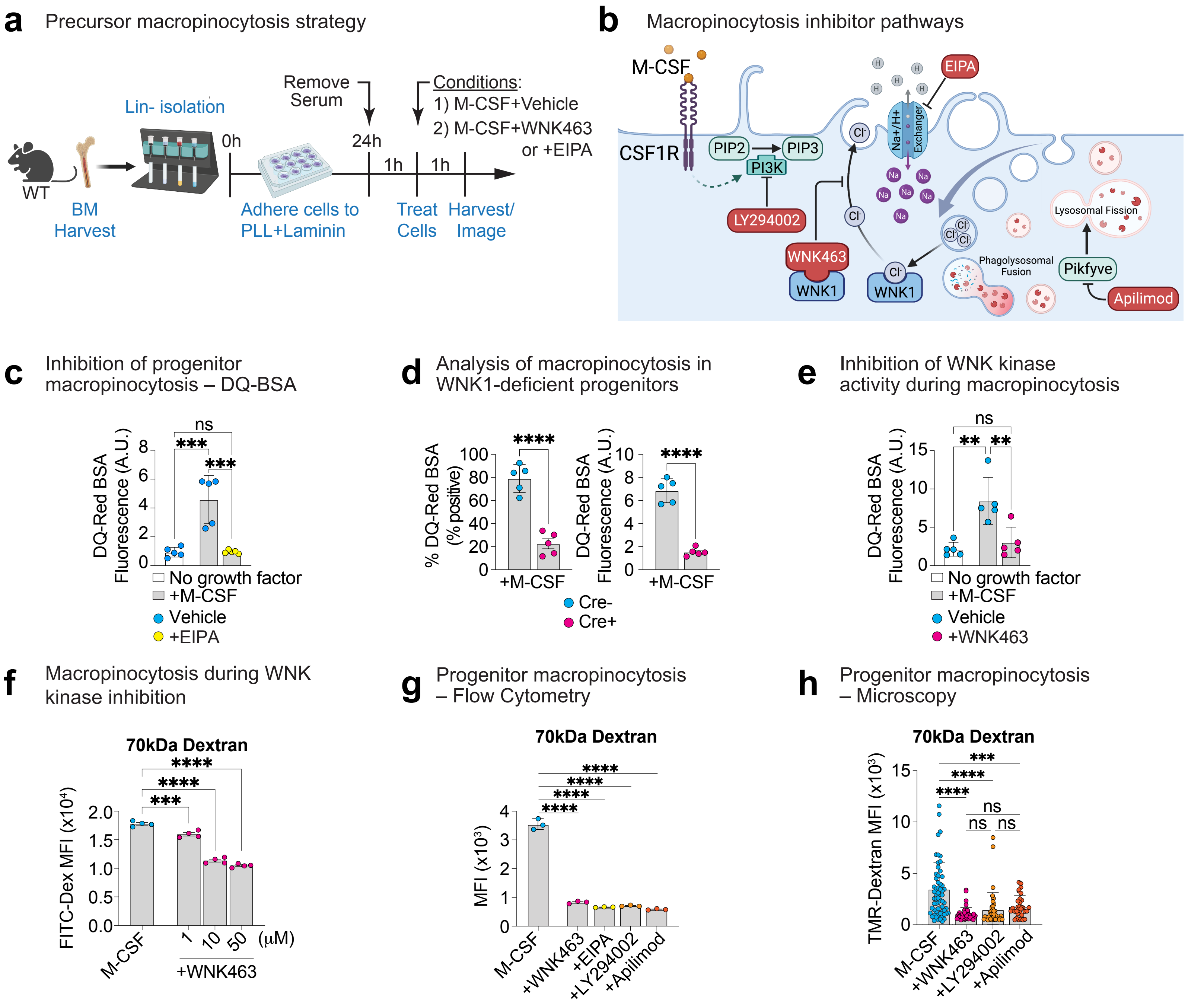
a
Precursor macropinocytosis strategy
WT
BM
Harvest
Lin- isolation
0h
Adhere cells to
PLL+Laminin
Remove
Serum
24h
1h
1h
Conditions:
1) M-CSF+Vehicle
2) M-CSF+WNK463
or +EIPA
Treat
Cells
Harvest/
Image
b
Macropinocytosis inhibitor pathways
M-CSF
CSF1R
PIP2
PIP3
PI3K
LY294002
EIPA
Na+/H+
Exchanger
WNK463
WNK1
WNK1
Phagolysosomal
Fusion
Lysosomal Fission
Pikfyve
Apilimod
c
Inhibition of progenitor
macropinocytosis – DQ-BSA
DQ-Red BSA
Fluorescence (A.U.)
ns
***
***
No growth factor
+M-CSF
Vehicle
+EIPA
d
Analysis of macropinocytosis in
WNK1-deficient progenitors
% DQ-Red BSA
(% positive)
****
+M-CSF
DQ-Red BSA
Fluorescence (A.U.)
****
+M-CSF
Cre-
Cre+
e
Inhibition of WNK kinase
activity during macropinocytosis
DQ-Red BSA
Fluorescence (A.U.)
ns
**
**
No growth factor
+M-CSF
Vehicle
+WNK463
f
Macropinocytosis during WNK
kinase inhibition
70kDa Dextran
FITC-Dex MFI (x10⁴)
****
****
***
M-CSF
1
10
50
(μM)
+WNK463
g
Progenitor macropinocytosis
– Flow Cytometry
70kDa Dextran
MFI (x10³)
****
****
****
****
M-CSF
+WNK463
+EIPA
+LY294002
+Apilimod
h
Progenitor macropinocytosis
– Microscopy
70kDa Dextran
TMR-Dextran MFI (x10³)
***
****
****
ns
ns
ns
M-CSF
+WNK463
+LY294002
+Apilimod

Supplementary Fig. 15. Analysis of M-CSF-stimulated macropinocytosis in bone marrow progenitors.

(a) Strategy for analyzing M-CSF-induced macropinocytosis using myeloid progenitors. Shown is the experimental strategy used to determine if bone marrow myeloid progenitors perform macropinocytosis in response to M-CSF and if WNK1 is required for this process.

(b) Methods for disrupting macropinocytosis. Shown is a model of macropinocytosis with strategies used to disrupt this process. EIPA - 5-[N-ethyl-N-isopropyl] amiloride. PIP2 - Phosphatidylinositol 4,5-bisphosphate. PIP3 - Phosphatidylinositol (3,4,5)-trisphosphate. PI3K - Phosphoinositide 3-kinase. PIKfyve - Phosphoinositide kinase, FYVE-type zinc finger containing.

(c) Bone marrow myeloid progenitors perform macropinocytosis in response to M-CSF. Analysis of DQ-Red BSA internalization in bone marrow myeloid progenitors isolated and stimulated with M-CSF. In conjunction with M-CSF, the putative macropinocytosis substrate DQ-Red bovine serum albumin (BSA) was added to cells and incubated for 1h in the presence of either vehicle or the macropinocytosis inhibitor EIPA. Quantitation (fluorescence intensity in arbitrary units (A.U.)) of DQ-Red BSA is shown. Data is representative of two independent experiments with two to three biological replicates per experiment (n=5 per condition). Data are shown as mean ± SEM. Statistical significance was determined via one-way ANOVA. ***p < .001, ns = not significant.

(d) WNK1 is required for M-CSF-stimulated macropinocytosis by bone marrow myeloid progenitors. Bone marrow myeloid progenitors from *Csf1r*$^{Cre+}$*; Wnk1*$^{fl/fl}$ (Cre+) or *Csf1*$^{Cre-}$*; Wnk1*$^{fl/fl}$ (Cre-) mice were isolated and stimulated with M-CSF and DQ-Red BSA as in **Fig. 3a**. Flow cytometry quantitation of frequencies of macropinocytotic (DQ-Red BSA+) progenitors (*left*) and fluorescence intensity in arbitrary units (A.U.)) of DQ-Red BSA (*right*) are shown. Data is from two independent experiments with two-three mice (biological replicates) per experiment (n=5 per condition). Data are shown as mean ± SEM. Statistical significance was determined via independent samples *t*-test. ****p < .0001.

(e) WNK kinase activity is required for M-CSF-stimulated macropinocytosis of DQ-Red BSA by bone marrow myeloid progenitors. WT bone marrow myeloid progenitors were isolated and stimulated with M-CSF and DQ-Red BSA as in **(d)**, except in the presence of vehicle or 10µm WNK463. Quantitation (fluorescence intensity in arbitrary units (A.U.)) of DQ-Red BSA+ is shown. Data is representative of two independent experiments with at least two-to-three mice (biological replicates) per experiment (n=5 per condition). Data are shown as mean ± SEM. Statistical significance was determined via one-way ANOVA. **p < .01, ns = not significant.

(f-h) M-CSF stimulates internalization of 70kDa Dextran by bone marrow myeloid progenitors via WNK kinase activity-dependent macropinocytosis. Experiments performed as in **Fig. 3a** with the WNK kinase activity inhibitor WNK463 (dose titration; **f**) or the macropinocytosis inhibitors EIPA, LY294002, or Apilimod (**g**) were added together with M-CSF. Shown is the summary plot of flow cytometry quantification of FITC-Dextran+ MFI. Data are from three independent experiments (n=3-4 per condition). **(h)** Time-lapse confocal microscopy analysis of M-CSF-induced internalization of 70kDa Dextran by bone marrow myeloid progenitors with WNK463, LY294002, or Apilimod. Quantitation (fluorescence intensity in arbitrary units) of TMR-Dextran+ myeloid progenitors are shown. Each dot is an individual cell, data pooled from three independent experiments. Data are shown as mean ± SEM. Statistical significance was determined via one-way ANOVA. ***p < .001, ****p < .0001, ns = not significant.

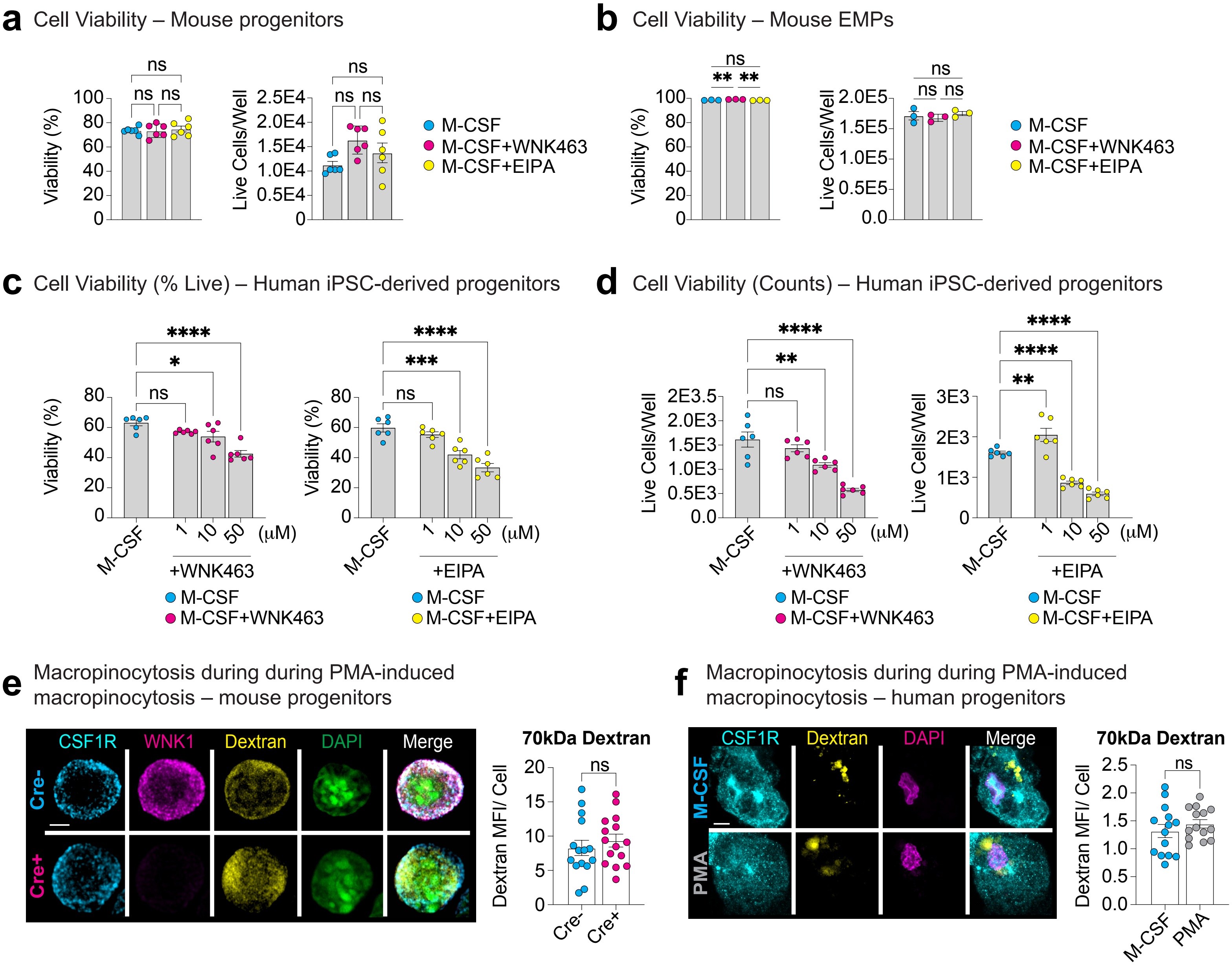
a
Cell Viability – Mouse progenitors
Viability (%)
Live Cells/Well
ns
M-CSF
M-CSF+WNK463
M-CSF+EIPA
b
Cell Viability – Mouse EMPs
Viability (%)
Live Cells/Well
ns
**
M-CSF
M-CSF+WNK463
M-CSF+EIPA
c
Cell Viability (% Live) – Human iPSC-derived progenitors
Viability (%)
****
*
***
ns
M-CSF
1
10
50
(μM)
+WNK463
+EIPA
M-CSF
M-CSF+WNK463
M-CSF
M-CSF+EIPA
d
Cell Viability (Counts) – Human iPSC-derived progenitors
Live Cells/Well
****
**
ns
M-CSF
1
10
50
(μM)
+WNK463
+EIPA
M-CSF
M-CSF+WNK463
M-CSF
M-CSF+EIPA
e
Macropinocytosis during during PMA-induced macropinocytosis – mouse progenitors
CSF1R
WNK1
Dextran
DAPI
Merge
Cre-
Cre+
70kDa Dextran
Dextran MFI/ Cell
ns
Cre-
Cre+
f
Macropinocytosis during during PMA-induced macropinocytosis – human progenitors
CSF1R
Dextran
DAPI
Merge
M-CSF
PMA
70kDa Dextran
Dextran MFI/ Cell
ns
M-CSF
PMA

Supplementary Fig. 16. Macropinocytosis assay cell viability.

(a, b) Analysis of cell viability as frequencies of propidium iodide negative cells (*left*) or total live cell yields (*right*) from mouse bone marrow progenitors (**a**) or mouse neonatal erythromyeloid progenitors (**b**) treated with 10µm WNK463 (pink dots) or 10µm EIPA (yellow dots) from **Fig. 4**. Data are shown as mean ± SEM. Statistical significance was determined via one-way ANOVA. ns = not significant.

(c, d) Analysis of cell viability as frequencies of propidium iodide negative cells (**c**) or total live cell yields (**d**) from human iPSC-derived myeloid progenitors treated with 1, 10 or 50µm of WNK463 (pink dots) or EIPA (yellow dots) from **Fig. 4**. Data are shown as mean ± SEM. Statistical significance was determined via one-way ANOVA. **p* < .05, ***p* < .01, ****p* < .001, *****p* < .0001, ns = not significant.

(**e**) Analysis of macropinocytosis in PMA-treated mouse myeloid progenitors. (*Left*) Representative fixed confocal microscopy images of bone marrow myeloid progenitors isolated from $Csf1r^{Cre+}$*; * $Wnk1^{fl/fl}$ (Cre+) or $Csf1^{Cre-}$*; * $Wnk1^{fl/fl}$ (Cre-) cultured with 70kDa Dextran (yellow) and PMA for 1h. Cells were then stained with CSF1R (cyan), WNK1 (magenta), and DAPI (green). Scale bar, 5µm. (*Right*) Quantitation of 70kDa Dextran uptake via MFI per cell on 3D projections of each cell, with n=15 FOVs per group. Data are from four independent experiments. Data are shown as mean ± SEM. Statistical significance was determined via independent samples *t*-test. ns = not significant.

(f) Analysis of macropinocytosis in PMA-treated human myeloid progenitors. (*Left*) Representative fixed confocal microscopy images of human iPSC-derived myeloid progenitors cultured with 70kDa Dextran (yellow) and PMA for 1h. Cells were then stained with CSF1R (cyan) and DAPI (magenta). Scale bar, 5µm. (*Right*) Quantitation of 70kDa Dextran uptake via MFI per cell, with n=14 FOVs per group. Data are from three independent experiments. Data are shown as mean ± SEM. Statistical significance was determined via independent samples *t*-test. ns = not significant.

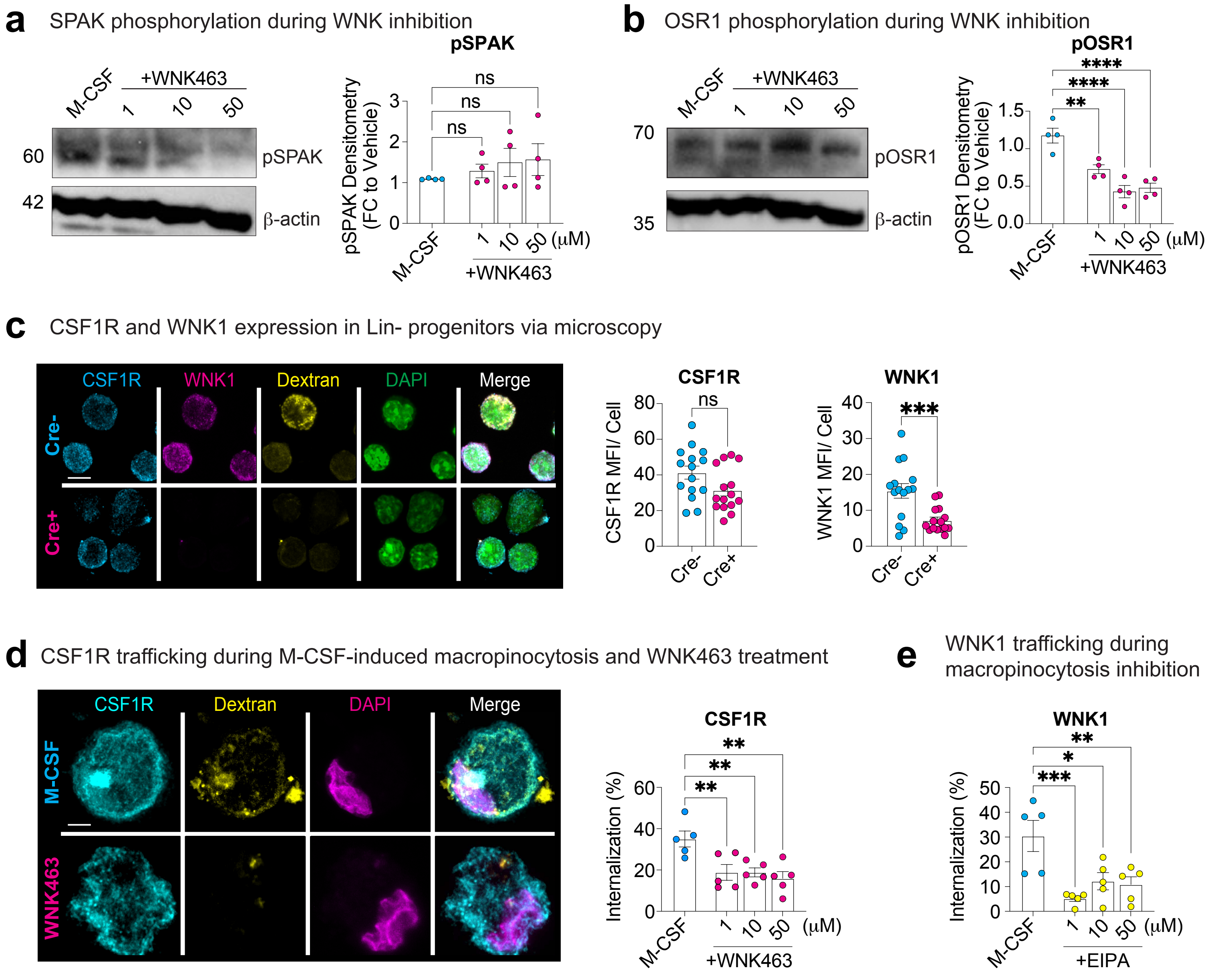
a
SPAK phosphorylation during WNK inhibition
M-CSF
+WNK463
1
10
50
60
42
pSPAK
β-actin
pSPAK
pSPAK Densitometry (FC to Vehicle)
ns
ns
ns
3
2
1
0
M-CSF
1
10
50
(μM)
+WNK463
b
OSR1 phosphorylation during WNK inhibition
M-CSF
+WNK463
1
10
50
70
35
pOSR1
β-actin
pOSR1
pOSR1 Densitometry (FC to Vehicle)
****
****
**
1.5
1.0
0.5
0.0
M-CSF
1
10
50
(μM)
+WNK463
c
CSF1R and WNK1 expression in Lin- progenitors via microscopy
CSF1R
WNK1
Dextran
DAPI
Merge
Cre-
Cre+
CSF1R
CSF1R MFI/ Cell
ns
80
60
40
20
0
Cre-
Cre+
WNK1
WNK1 MFI/ Cell
***
40
30
20
10
0
Cre-
Cre+
d
CSF1R trafficking during M-CSF-induced macropinocytosis and WNK463 treatment
CSF1R
Dextran
DAPI
Merge
M-CSF
WNK463
CSF1R
Internalization (%)
**
**
**
60
40
20
0
M-CSF
1
10
50
(μM)
+WNK463
e
WNK1 trafficking during macropinocytosis inhibition
WNK1
Internalization (%)
**
*
***
50
40
30
20
10
0
M-CSF
1
10
50
(μM)
+EIPA

Supplementary Fig. 17. Inhibition of WNK kinase activity impairs CSF1R internalization and WNK1 translocation.

(a, b) Analysis of SPAK and OSR1 phosphorylation during M-CSF-stimulated macropinocytosis in human myeloid progenitors. Human iPSC-derived myeloid progenitors were stimulated with M-CSF, then assayed for SPAK (**a**) or OSR1 (**b**) phosphorylation after 1h. Shown are representative Phos-tag western blot (*left*) and quantitation (*right*) of pSPAK or pOSR1 phosphorylation. pSPAK and pOSR1 levels were normalized to loading control (β-actin, 42kDa) and then calculated as a fold change (FC) relative to control. Data are pooled from two independent experiments with two biological replicates per condition/experiment. Data are shown as mean ± SEM. Statistical significance was determined via one-way ANOVA. $^{**}p < .01$, $^{****}p < .0001$, ns = not significant.

(**c**) Analysis of CSF1R and WNK1 expression in M-CSF-treated mouse wildtype (Cre-) and WNK1-deficient (Cre+) myeloid progenitors. (*Left*) Representative fixed confocal microscopy images of myeloid progenitors from experiments in **Fig. 5c**. Scale bar, 5µm. (*Right*) Quantitation of CSF1R and WNK1 protein expression. Each dot represents the average MFI per field of view (FOV) divided by the number of cells per region, with n=15 FOVs per group. Data are from four independent experiments. Data are shown as mean ± SEM. Statistical significance was determined via independent samples *t*-test. $^{***}p < .001$, ns = not significant.

(d) Analysis of CSF1R internalization in WNK463-treated human myeloid progenitors. (*Left*) Representative fixed confocal microscopy images of human myeloid progenitors treated with 70kDa Dextran (yellow), M-CSF, and either 1µM, 10µM (shown), or 50µM WNK463 for 1h. Cells were subsequently stained for CSF1R (cyan) and DAPI (magenta). Scale bar, 5µm. (*Right*) Quantitation of CSF1R internalization based on Manders' Overlap Coefficient (MCC%), with each dot representing the MCC% overlap per cell, with n=5 FOVs per group. Data representative of three independent experiments. Data are shown as mean ± SEM. Statistical significance was determined via one-way ANOVA. $^{**}p < .01$.

(e) Analysis of macropinocytosis-dependent WNK1 nuclear translocation in human myeloid progenitors. Human iPSC-derived myeloid progenitors were stimulated with M-CSF in the presence of vehicle or EIPA at the indicated concentrations for 1h. Shown is quantitation of WNK1 nuclear localization based on MCC% with DAPI. Each dot represents the WNK1/DAPI MCC% overlap per cell, with n=5 FOVs per group. Data are from five independent experiments. Data are shown as mean ± SEM. Statistical significance was determined via one-way ANOVA. $^{*}p < .05$, $^{**}p < .01$, $^{***}p < .001$.

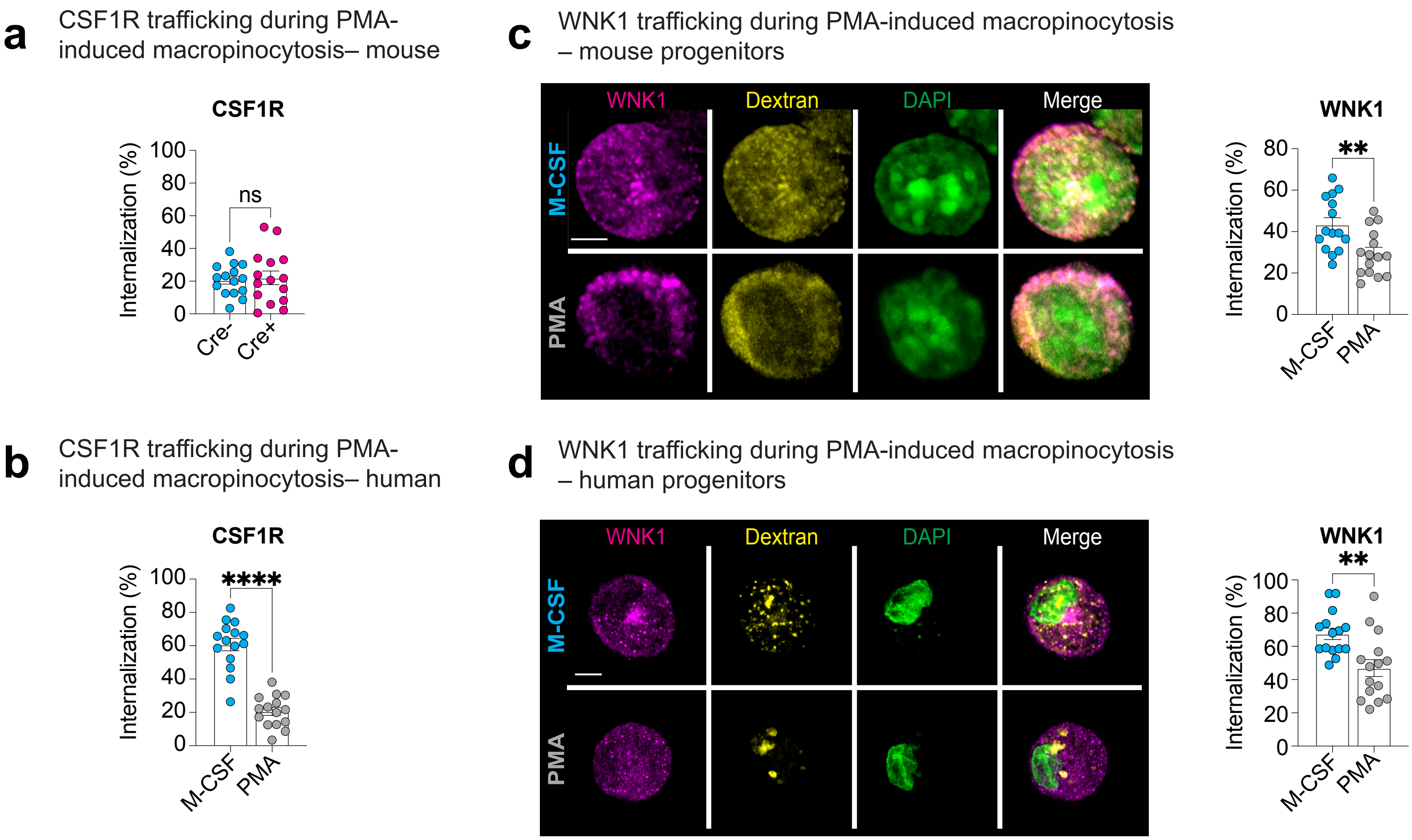

a
CSF1R trafficking during PMA-induced macropinocytosis– mouse
CSF1R
ns
Internalization (%)
100
80
60
40
20
0
Cre-
Cre+
b
CSF1R trafficking during PMA-induced macropinocytosis– human
CSF1R
****
Internalization (%)
M-CSF
PMA
c
WNK1 trafficking during PMA-induced macropinocytosis – mouse progenitors
WNK1
Dextran
DAPI
Merge
M-CSF
PMA
WNK1
**
Internalization (%)
d
WNK1 trafficking during PMA-induced macropinocytosis – human progenitors
WNK1
Dextran
DAPI
Merge
M-CSF
PMA
WNK1
**
Internalization (%)

Supplementary Fig. 18. Analysis of CSF1R internalization and WNK1 localization during PMA-induced macropinocytosis.

(**a**) Analysis of CSF1R internalization in PMA-treated mouse wildtype (Cre-) and WNK1-deficient (Cre+) myeloid progenitors. Analysis of experiments performed in Extended Data Fig. 16e. Quantitation of CSF1R internalization based on MCC% analyzed on 3D projections of each cell. Each dot represents the CSF1R MCC% overlap per cell, with n=15 FOVs per group. Data are from three independent experiments. Data are shown as mean ± SEM. Statistical significance was determined via independent samples *t*-test. ns = not significant.

(b) Analysis CSF1R internalization in PMA-treated human myeloid progenitors. Analysis of experiments performed in Extended Data Fig. 16f. Quantitation of CSF1R internalization based on MCC% analyzed on 3D projections of each cell. Each dot represents the CSF1R MCC% overlap per cell, with n=15 FOVs per group. Data are from three independent experiments. Data are shown as mean ± SEM. Statistical significance was determined via independent samples *t*-test. ****$p < .0001$.

(c) Analysis of WNK1 nuclear translocation in PMA-treated mouse myeloid progenitors. (*Left*) Representative fixed confocal microscopy images of mouse bone marrow myeloid progenitors cultured with 70kDa Dextran (yellow) and M-CSF or PMA for 1h. Cells were then stained with WNK1 (magenta) and DAPI (green). Scale bar, 5µm. (*Right*) Quantitation of WNK1 nuclear translocation based on MCC% with DAPI on 3D projections of each cell. Each dot represents the WNK1/DAPI MCC% overlap per cell, with n=15 FOVs per group. Data are from three independent experiments. Data are shown as mean ± SEM. Statistical significance was determined via independent samples *t*-test. **$p < .01$.

(d) Analysis of WNK1 nuclear translocation in PMA-treated human myeloid progenitors. (*Left*) Representative fixed confocal microscopy images of human iPSC-derived EMPs cultured with 70kDa Dextran (yellow) and PMA for 1h. Cells were then stained with WNK1 (magenta) and DAPI (green). Scale bar, 5µm. (*Right*) Quantitation of WNK1 nuclear translocation based on MCC% with DAPI on 3D projections of each cell. Each dot represents the WNK1/DAPI MCC% overlap per cell, with n=20 FOVs per group. Data are from three independent experiments. Data are shown as mean ± SEM. Statistical significance was determined via independent samples *t*-test. *$p < .05$.

a IRF8 expression during macropinocytosis inhibition – mRNA expression

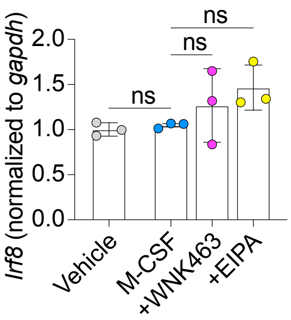

b IRF8 induction during WNK1 overexpression – mRNA expression

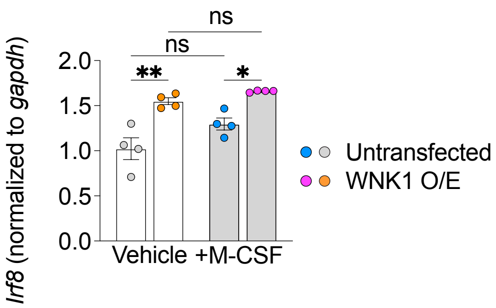

Supplementary Fig. 19. *Irf8* mRNA expression during WNK1 inhibition and overexpression.

(a) Quantitative PCR analysis of *Irf8* in M-CSF-treated mouse myeloid progenitors. Mouse bone marrow myeloid progenitors were stimulated in the presence of vehicle, M-CSF, or M-CSF with 10µM WNK463 or EIPA for 24h, then isolated and analyzed for *Irf8* expression via qPCR. Data representative of three independent experiments. Data are shown as mean ± SEM. Statistical significance was determined via independent samples *t*-test. ns = not significant. See also **Fig. 5f** for corresponding analysis of IRF8 protein.

(b) Quantitative PCR analysis of *Irf8* in mouse wildtype cells overexpressing functional WNK1-mRuby. Mouse bone marrow myeloid progenitors transfected with WNK1-mRuby were stimulated with M-CSF 24h, then isolated and analyzed for *Irf8* expression via qPCR. n=4 wildtype mice per group. Data are shown as mean ± SEM Statistical significance was determined via one-way ANOVA. *$p < .05$, **$p < .01$. ns = not significant. See also **Fig. 5h** for corresponding analysis of IRF8 protein.

Supplementary Table 1. Mouse FACS antibodies

Antibody	Clone	Fluorochrome	Company	Dilution
CD16/32	2.4G2	Unconjugated	BioXCell	1:50
CD45	30-F11	AF700	BioLegend	1:400
CD11b	M1/70	APC- e780, FITC	Invitrogen	1:800
SiglecF	S17007L	APC	BioLegend	1:400
F4/80	BM8	FITC	Invitrogen	1:200
F4/80	BM8	BV605, APC	BioLegend	1:200
Ly6C	HK1.4	PerCP-Cy5.5	BioLegend	1:400
Ly6C	AL-21	PE	BD Pharmingen	1:400
Ly6G	1A8	PE-Cy7	Tonbo Biosciences	1:200
Ly6G	1A8	PE	BioLegend	1:200
CD11c	N418	FITC	Invitrogen	1:800
CD11c	N418	PE-Cy7, BV785	BioLegend	1:800
MHCII	M5/114.15.2	BV421, BV711	BioLegend	1:400
CD206	C068C2	BV650, PE/Dazzle	BioLegend	1:200
CSF1R(CD115)	AFS98	PerCP-Cy5.5	BioLegend	1:400
CX_3CR1	SA011F11	BV785	BioLegend	1:400
CD45.1	A20	BV711	BioLegend	1:400
CD4	RM4-5	PE-594	BioLegend	1:400
CD8a	53-6.7	BV605	BioLegend	1:400
CD19	6D5	PerCP-Cy5.5, BV421	BioLegend	1:400
CD86	GL-1	APC-Fire	BioLegend	1:400
CD64	X54-5/7.1	APC, BV421, FITC	BioLegend	1:200
CD24	M1/69	BV711	BD Pharmingen	1:400
CD163	S15049F	PE	BioLegend	1:400
Lineage Cocktail	145-2C11, RB6-8C5, M1/70, RA3-6B2, Ter-119	FITC	BioLegend	1:200
c-Kit	2B8	BV421	BD Pharmingen	1:200
Sca-1	D7	PE	BioLegend	1:200
Flt3	A2F10	APC	BioLegend	1:200
CD150	PE-Cy7	TC15-12F12.2	BioLegend	1:200
CD48	HM48-1	BV605	BioLegend	1:200

Supplementary Table 2. Mouse immunofluorescence antibodies

Antibody	Clone	Company	Catalog #	Dilution
Iba1	Rabbit/Polyclonal	Wako Chemicals	019-19741	1:500
Ly6G-PE	Rat/1A8	BioLegend	127607	1:200
CSF1R	Rabbit/Polyclonal	Invitrogen	PA5-25974	1:500
Ms/Rat WNK1	Goat/Polyclonal	R&D Systems	AF2849	1:250
Langerin (CD207)	Mouse/4C7	Biolegend	144203	1:500

Supplementary Table 3. Human FACS antibodies

Antibody	Clone	Fluorochrome	Company	Dilution
FcR Block	Not listed	Unconjugated	Miltenyi	1:20
CD14	HCD14	FITC	BioLegend	1:200
CD11B	ICRF44	Pe-Cy7	BioLegend	1:400
CD66B	G10F5	AF700	BioLegend	1:200
CD86	IT2.2	PacBlue	BioLegend	1:200
CD206	15-2	BV711	BioLegend	1:200
CSF1R (CD115)	9-4D2-1E4	PE	BD Pharmingen	1:400
CD11C	3.9	BV605	BioLegend	1:400
HLA-DR	L243	PerCP-Cy5.5	BioLegend	1:200

Supplementary Table 4. Human immunofluorescence antibodies

Antibody	Clone	Company	Catalog #	Dilution
FcR Block	Not listed	Miltenyi	Miltenyi	1:50
CSF1R	Rabbit/Polyclonal	Invitrogen	PA5-25974	1:500
WNK1	Rabbit/Polyclonal	Invitrogen	711356	1:250

Supplementary Table 5. Mouse western blot antibodies

Antibody	Host/Clone	Company	Catalog #	Dilution
Ms/Rat WNK1	Goat/Polyclonal	R&D Systems	AF2849	1:250
IRF8 (ICSBP)	Mouse/E-9	Santa Cruz	sc-365042	1:250
β-actin	Mouse/C4	Santa Cruz	sc-47778	1:20000

Supplementary Table 6. Human western blot antibodies

Antibody	Host/Clone	Company	Catalog #	Dilution
WNK1	Rabbit/Oligoclonal	Invitrogen	711356	1:500
OSR1	Rabbit/Polyclonal	EMD Millipore	07-2264	1:500
SPAK	Rabbit/F7T1K	Cell Signaling	2281S	1:500
β-actin	Mouse/C4	Santa Cruz	sc-47778	1:20000
