# Supplementary material for: WNK1 mediates M-CSF-induced macropinocytosis to enforce macrophage lineage fidelity
Source: Nat Commun. 2025 May 28;16:4945. doi: 10.1038/s41467-025-59901-0 (PMC12120055; doi:10.1038/s41467-025-59901-0)
Supplement: Supplementary file 2 — Description of Additional Supplementary Files [file 41467_2025_59901_MOESM2_ESM.pdf]

Description of Additional Supplementary Files

Supplementary Data 1. Complete blood count (CBC), chemistry panel, and gross/histopathological analysis results comparing *Csf1r*$^{Cre+}$*; Wnk1*$^{fl/fl}$ (Cre+; n=3, two male, one female) vs. *Csf1*$^{Cre-}$*; Wnk1*$^{fl/fl}$ (Cre-; n=3, three female) mice. Analyses were performed and analyzed by the MSKCC Pathology Core.

Supplementary Data 2. List of genes making up indicated clusters in Figure 2f.
