# Supplementary material for: WNK1 mediates M-CSF-induced macropinocytosis to enforce macrophage lineage fidelity
Source: Nat Commun. 2025 May 28;16:4945. doi: 10.1038/s41467-025-59901-0 (PMC12120055; doi:10.1038/s41467-025-59901-0)
Supplement: Supplementary file 5 — Reporting Summary [file 41467_2025_59901_MOESM5_ESM.pdf]

Corresponding author(s): Justin S. A. Perry and Alissa J. Trzeciak
Last updated by author(s): Apr 4, 2025

Reporting Summary

Nature Portfolio wishes to improve the reproducibility of the work that we publish. This form provides structure for consistency and transparency in reporting. For further information on Nature Portfolio policies, see our Editorial Policies and the Editorial Policy Checklist.

Statistics

For all statistical analyses, confirm that the following items are present in the figure legend, table legend, main text, or Methods section.

n/a	Confirmed	
☐	☒	The exact sample size (*n*) for each experimental group/condition, given as a discrete number and unit of measurement
☐	☒	A statement on whether measurements were taken from distinct samples or whether the same sample was measured repeatedly
☐	☒	The statistical test(s) used AND whether they are one- or two-sided<br>*Only common tests should be described solely by name; describe more complex techniques in the Methods section.*
☒	☐	A description of all covariates tested
☐	☒	A description of any assumptions or corrections, such as tests of normality and adjustment for multiple comparisons
☐	☒	A full description of the statistical parameters including central tendency (e.g. means) or other basic estimates (e.g. regression coefficient) AND variation (e.g. standard deviation) or associated estimates of uncertainty (e.g. confidence intervals)
☐	☒	For null hypothesis testing, the test statistic (e.g. *F*, *t*, *r*) with confidence intervals, effect sizes, degrees of freedom and *P* value noted<br>*Give P values as exact values whenever suitable.*
☒	☐	For Bayesian analysis, information on the choice of priors and Markov chain Monte Carlo settings
☒	☐	For hierarchical and complex designs, identification of the appropriate level for tests and full reporting of outcomes
☒	☐	Estimates of effect sizes (e.g. Cohen's *d*, Pearson's *r*), indicating how they were calculated

Our web collection on statistics for biologists contains articles on many of the points above.

Software and code

Policy information about availability of computer code

Data collection	Flow cytometry data was collected using Attune NxT Software v3.2.1. Photographs were taken with an iPhone 14 Pro. Imaging was collected and analyzed using Zen Blue v3.0 or EVOS m5000 software v1.4. Cartoon diagrams were created with BioRender.com. Chemiluminescence was detected with Invitrogen iBright 1500.
Data analysis	We used Graphpad Prism 10 for all statistical and graphical analysis. We used Fiji to analyze microscopy images. We used Python v3.8 to generate scRNA-seq plots. The cell-gene count matrix was constructed using the Sequence Quality Control (SEQC) package91. Viable cells were identified based on library size and complexity, with ‘cells’ exhibiting >20% of transcripts derived from mitochondria excluded from analysis. Resulting matrix data were further processed and analyzed using R version 4.2.2 (R Core Team 2022) and the Seurat package version 4.3.094. Seurat objects were created using only genes appearing in at least three cells. Cells were further filtered to exclude those with <200 genes detected or >7,000 genes detected. Read counts were then normalized within each sample using the SCTransform function with the glmGamPoi method, percent mitochondrial reads as the vars.to.regress argument, and vst.flavor v2. The RunPCA function was then run with npcs set to 30. The graph representing cells with similar expression patterns was generated with the FindNeighbors function using the 30 largest principal components. Cell clusters were generated using the Louvain algorithm implemented by the FindClusters function with a resolution parameter equal to 0.6. Marker genes for each cluster were determined using the Wilcoxon test on the SCT normalized counts after running the PrepSCTFindMarkers function using the function FindAllMarkers and including only genes with log fold changes >0.25 and Bonferroni-corrected P values <0.01. Dimensionality reduction by Uniform Manifold Approximation and Projection was performed using the RunUMAP function with the 30 largest principal components. All visualizations of scRNA-seq data were generated using the Seurat94, scCustomize, and ggplot2 packages. All code is available upon request.

For manuscripts utilizing custom algorithms or software that are central to the research but not yet described in published literature, software must be made available to editors and reviewers. We strongly encourage code deposition in a community repository (e.g. GitHub). See the Nature Portfolio guidelines for submitting code & software for further information.

Data

Policy information about availability of data

All manuscripts must include a data availability statement. This statement should provide the following information, where applicable:

- Accession codes, unique identifiers, or web links for publicly available datasets
- A description of any restrictions on data availability
- For clinical datasets or third party data, please ensure that the statement adheres to our policy

Single cell RNA sequencing data for this experiment have been deposited at the Gene Expression Omnibus under accession number GSE196336. All data are included in the Supplementary Information or available from the authors, as are unique reagents used in this Article. The raw numbers for charts and graphs are available in the Source Data file whenever possible. All other data are available from the corresponding author upon reasonable request.

Field-specific reporting

Please select the one below that is the best fit for your research. If you are not sure, read the appropriate sections before making your selection.

[x] Life sciences [ ] Behavioural & social sciences [ ] Ecological, evolutionary & environmental sciences

For a reference copy of the document with all sections, see nature.com/documents/nr-reporting-summary-flat.pdf

Life sciences study design

All studies must disclose on these points even when the disclosure is negative.

Sample size	No statistical tests were used to determine in vitro sample size. In vivo sample size was determined based on power analysis using power of 0.8 and a Type I error rate (a) of 5%, to determine the number of mice needed per experiment to detect a 50% change assuming a 25% standard deviation.
Data exclusions	No data was excluded from this manuscript.
Replication	In vitro experiments were done in technical triplicates with at least three biological replicates. In vivo experiments were done at least twice, with a minimum of 3 biological replicates per experiment. Instances where experiments required pooling data points were done up to 5 times.
Randomization	Experimental mice, when possible, were randomized in all in vivo experiments, and littermate controls were used from the same cage as experimental mice.
Blinding	Injections of P2 was blinded to mouse genotype until P21. Microscopy pictures were numbered by one person and analyzed by another before graphing data into respective conditions or genetic backgrounds.

Reporting for specific materials, systems and methods

We require information from authors about some types of materials, experimental systems and methods used in many studies. Here, indicate whether each material, system or method listed is relevant to your study. If you are not sure if a list item applies to your research, read the appropriate section before selecting a response.

Materials & experimental systems

n/a	Involved in the study	
[ ]	[x]	Antibodies
[x]	[ ]	Eukaryotic cell lines
[x]	[ ]	Palaeontology and archaeology
[ ]	[x]	Animals and other organisms
[ ]	[x]	Human research participants
[x]	[ ]	Clinical data
[x]	[ ]	Dual use research of concern

Methods

n/a	Involved in the study	
[x]	[ ]	ChIP-seq
[ ]	[x]	Flow cytometry
[x]	[ ]	MRI-based neuroimaging

Antibodies

Antibodies used	Mouse FACS Antibodies:<br>CD16/32 2.4G2 Unconjugated BioXCell 1:50<br>CD45 30-F11 AF700 BioLegend 1:400<br>CD11b M1/70 APC- e780, FITC Invitrogen 1:800<br>SiglecF S17007L (APC BioLegend 1:400<br>F4/80 BM8 FITC Invitrogen 1:200

F4/80 BM8 BV605, APC BioLegend 1:200
Ly6C HK1.4 PerCP-Cy5.5 BioLegend 1:400
Ly6C AL-21 PE BD Pharmingen 1:400
Ly6G 1A8 PE-Cy7 Tonbo Biosciences 1:200
Ly6G 1A8 PE BioLegend 1:200
CD11c N418 FITC Invitrogen 1:800
CD11c N418 PE-Cy7, BV785 BioLegend 1:800
MHCII M5/114.15.2 BV421, BV711 BioLegend 1:400
CD206 C068C2 BV650, PE/Dazzle BioLegend 1:200
CSF1R(CD115) AFS98 PerCP-Cy5.5 BioLegend 1:400
CX3CR1 SA011F11 BV785 BioLegend 1:400
CD45.1 A20 BV711 BioLegend 1:400
CD4 RM4-5 PE-594 BioLegend 1:400
CD8a 53-6.7 BV605 BioLegend 1:400
CD19 6D5 PerCP-Cy5.5, BV421 BioLegend 1:400
CD86 GL-1 APC-Fire BioLegend 1:400
CD64 X54-5/7.1 APC, BV421, FITC BioLegend 1:200
CD24 M1/69 BV711 BD Pharmingen 1:400
CD163 S15049F PE BioLegend 1:400
Lineage Cocktail (145-2C11, RB6-8C5, M1/70, RA3-6B2, Ter-119) FITC BioLegend 1:200
c-Kit 2B8 BV421 BD Pharmingen 1:200
Sca-1 D7 PE BioLegend 1:200
Flt3 A2F10 APC BioLegend 1:200
CD150 PE-Cy7 TC15-12F12.2 BioLegend 1:200
CD48 HM48-1 BV605 BioLegend 1:200

Mouse Immunofluorescence Antibodies:
Iba1 Rabbit/Polyclonal Wako Chemicals 019-19741 1:500
Ly6G-PE Rat/1A8 BioLegend 127607 1:200
CSF1R Rabbit/Polyclonal Invitrogen PA5-25974 1:500
Ms/Rat WNK1 Goat/Polyclonal R&D Systems AF2849 1:250
Langerin (CD207) Mouse/4C7 Biolegend 144203 1:500
Human FACS Antibodies:
FcR Block Clone Not listed Unconjugated Miltenyi 1:20
CD14 HCD14 FITC BioLegend 1:200
CD11B ICRF44 Pe-Cy7 BioLegend 1:400
CD66B G10F5 AF700 BioLegend 1:200
CD86 IT2.2 PacBlue BioLegend 1:200
CD206 15-2 BV711 BioLegend 1:200
CSF1R (CD115) 9-4D2-1E4 PE BD Pharmingen 1:400
CD11C 3.9 BV605 BioLegend 1:400
HLA-DR L243 PerCP-Cy5.5 BioLegend 1:200

Human Immunofluorescence Antibodies:
FcR Block Not listed Miltenyi Miltenyi 1:50
CSF1R Rabbit/Polyclonal Invitrogen PA5-25974 1:500
WNK1 Rabbit/Polyclonal Invitrogen 711356 1:250

Mouse Western Blot Antibodies:
Antibody Host/Clone Company Catalog # Dilution
Ms/Rat WNK1 Goat/Polyclonal R&D Systems AF2849 1:250
IRF8 (ICSBP) Mouse/E-9 Santa Cruz sc-365042 1:250
β-actin Mouse/C4 Santa Cruz sc-47778 1:20000

Human Western Blot Antibodies:
WNK1 Rabbit/Oligoclonal Invitrogen 711356 1:500
OSR1 Rabbit/Polyclonal EMD Millipore 07-2264 1:500
SPAK Rabbit/F7T1K Cell Signaling 2281S 1:500
β-actin Mouse/C4 Santa Cruz sc-47778 1:20000

Validation

All antibody lots are routinely tested by the manufacturer, found at:
https://www.biolegend.com/reproducibility
https://www.bdbiosciences.com/en-us/products/reagents/flow-cytometry-reagents/research-reagents/quality-and-reproducibility
https://www.thermofisher.com/us/en/home/life-science/antibodies/invitrogen-antibody-validation.html

Animals and other organisms

Policy information about studies involving animals; ARRIVE guidelines recommended for reporting animal research

Laboratory animals	Animals were housed at the Memorial Sloan Kettering Cancer Center (MSKCC) animal facility under specific pathogen free (SPF) conditions on a 12-hour light/dark cycle under ambient conditions with free access to food and water. Csf1r-iCre and CD45.1 C57BL/6 mice were purchased from Jackson Laboratories. Wnk1 fl/fl animals were a donation from Jian Xie and Chou-Long Huang from the University of Iowa. Both males and females between 2-4 weeks of age were used for all experiments except monocyte adoptive transfer experiments, where CD45.1 mice were males and females 8-12 weeks old.
Wild animals	No wild animals were used in this study.
Field-collected samples	No field-collected samples were used in this study.
Ethics oversight	All animal procedures were performed according to the protocols provided by the Institutional Animal Care and Use Committee (IACUC) of Memorial Sloan Kettering Cancer Center.

Note that full information on the approval of the study protocol must also be provided in the manuscript.

Human research participants

Policy information about studies involving human research participants

Population characteristics	Whole blood was obtained from the New York Blood Center. No specific demographics were requested, and male and female donors were both used. All samples were de-identified.
Recruitment	Participants were blood donation volunteers. This study did not discriminate age, race, or gender when receiving PBMC samples.
Ethics oversight	This study's protocol was approved by the New York Blood Center.

Note that full information on the approval of the study protocol must also be provided in the manuscript.

Flow Cytometry

Plots

Confirm that:

- [x] The axis labels state the marker and fluorochrome used (e.g. CD4-FITC).
- [x] The axis scales are clearly visible. Include numbers along axes only for bottom left plot of group (a 'group' is an analysis of identical markers).
- [x] All plots are contour plots with outliers or pseudocolor plots.
- [x] A numerical value for number of cells or percentage (with statistics) is provided.

Methodology

Sample preparation	Reported under Methods in "FACS staining and analysis". Blood was obtained via cardiac puncture. Organs were obtained after perfusion with cold PBS with EDTA, minced, enzyme digested, homogenized, and separated via density gradient. Bone marrow cells were obtained by flushing femurs and tibias for bone marrow characterization, BMDM culture, and progenitor isolation. Cells were washed once and filtered before staining. Samples were kept and stained on ice after collection. Staining was protected from light throughout.
Instrument	Data were collected on an Attune NxT flow cytometer (ThermoFisher).
Software	Data were analyzed with FlowJo v10.8.1 (Treestar, Inc).
Cell population abundance	Purity of isolated samples was achieved by antibody staining and FACS. Live cells were greater than 80% in all experiments.
Gating strategy	Outlined in Extended Data Fig. 2. Standard leukocyte gating was applied and doublet exclusion was achieved via FSC-W vs FSC-H. All cells were gated on Aqua negative live populations. All myeloid cells were first gated on live CD45+ CD11c- CD11b+ (except for lung macrophages). Monocytes (Ly6C high Ly6G-), Neutrophils (Ly6C+ Ly6G+), and tissue-resident macrophages were stained as follows: microglia (Ly6C- Ly6G- F4/80+ CX3CR1 high), alveolar (CD11c high CD11b low SiglecF+), intraglomerular (Ly6C- Ly6G- F4/80+), Kupffer (Ly6C- Ly6G- F4/80+), and red pulp (Ly6C- Ly6G- F4/80+ CD163+). HSC compartments were stained on live, c-Kit+ Sca-1+, and further subgated into LT-HSC (CD48- CD150+), ST-HSC (CD48- CD150-), MPP2s (Flt3- CD48+ CD150+), MPP3s (Flt3- CD48+ CD150-), and MPP4s (Flt3+ CD48+ CD150-). For some in vitro experiments, singlets and live cells were similarly gated, then analyzed for DQ-Red BSA or MQAE MFIs. See Figs 1d, 2c,d, and extended data Fig. 2.

- [x] Tick this box to confirm that a figure exemplifying the gating strategy is provided in the Supplementary Information.

March 2021
